# DNA methylation-based classifier and gene expression signatures detect BRCAness in osteosarcoma

**DOI:** 10.1371/journal.pcbi.1009562

**Published:** 2021-11-11

**Authors:** Maxim Barenboim, Michal Kovac, Baptiste Ameline, David T. W. Jones, Olaf Witt, Stefan Bielack, Stefan Burdach, Daniel Baumhoer, Michaela Nathrath

**Affiliations:** 1 Department of Pediatrics and Children’s Cancer Research Center, Klinikum rechts der Isar, Technical University of Munich, School of Medicine, Munich, Germany; 2 University Hospital Basel and University of Basel, Bone Tumour Reference Centre at the Institute of Pathology, Basel, Switzerland; 3 Faculty of Informatics and Information Technologies, Slovak University of Technology, Bratislava, Slovakia; 4 Hopp Children’s Cancer Center Heidelberg (KiTZ), Heidelberg, Germany; 5 University Hospital Heidelberg, Hematology and Immunology at the Department of Pediatric Oncology, Heidelberg, Germany; 6 Klinikum Stuttgart–Olgahospital, Stuttgart Cancer Center, Pediatrics 5 (Oncology, Hematology, Immunology), Stuttgart, Germany; 7 CCC München—Comprehensive Cancer Center, DKTK German Cancer Consortium, Munich, Germany; 8 Klinikum Kassel, Department of Pediatric Oncology, Kassel, Germany; University at Buffalo - The State University of New York, UNITED STATES

## Abstract

Although osteosarcoma (OS) is a rare cancer, it is the most common primary malignant bone tumor in children and adolescents. BRCAness is a phenotypical trait in tumors with a defect in homologous recombination repair, resembling tumors with inactivation of BRCA1/2, rendering these tumors sensitive to poly (ADP)-ribose polymerase inhibitors (PARPi). Recently, OS was shown to exhibit molecular features of BRCAness. Our goal was to develop a method complementing existing genomic methods to aid clinical decision making on administering PARPi in OS patients. OS samples with DNA-methylation data were divided to BRCAness-positive and negative groups based on the degree of their genomic instability (n = 41). Methylation probes were ranked according to decreasing variance difference between two groups. The top 2000 probes were selected for training and cross-validation of the random forest algorithm. Two-thirds of available OS RNA-Seq samples (n = 17) from the top and bottom of the sample list ranked according to genome instability score were subjected to differential expression and, subsequently, to gene set enrichment analysis (GSEA). The combined accuracy of trained random forest was 85% and the average area under the ROC curve (AUC) was 0.95. There were 449 upregulated and 1,079 downregulated genes in the BRCAness-positive group (fdr < 0.05). GSEA of upregulated genes detected enrichment of DNA replication and mismatch repair and homologous recombination signatures (FWER < 0.05). Validation of the BRCAness classifier with an independent OS set (n = 20) collected later in the course of study showed AUC of 0.87 with an accuracy of 90%. GSEA signatures computed for this test set were matching the ones observed in the training set enrichment analysis. In conclusion, we developed a new classifier based on DNA-methylation patterns that detects BRCAness in OS samples with high accuracy. GSEA identified genome instability signatures. Machine-learning and gene expression approaches add new epigenomic and transcriptomic aspects to already established genomic methods for evaluation of BRCAness in osteosarcoma and can be extended to cancers characterized by genome instability.

## Author summary

Osteosarcoma (OS) is the most common primary malignant tumor of bone in children and young adults with poor prognosis for patients with refractory or metastatic disease. A common feature, so-called BRCAness, exists in multiple cancers including OS and is characterized by homologous recombination deficiency. Tumors exhibiting BRCAness have been shown to respond to therapy with PARP inhibitors. Currently, BRCAness is mostly assessed by the genomic instability score. This method based on the DNA sequencing requires normal tissue DNA as control and is vulnerable to subjective interpretation of "genomic scarring" events. In this study, we implemented a classifier based on DNA methylation patterns. It is capable of detecting BRCAness in OS samples and does not require control tissue DNA. Therefore, it has the potential to support clinical decision making on administering PARPi in OS patients. We further corroborated the presence of BRCAness in OS by detecting homologous recombination signatures through gene expression analysis.

This is a *PLOS Computational Biology* Methods paper.

## Introduction

Osteosarcoma (OS) is the most common primary malignant tumor of bone in children and young adults [1]. There is a critical need for highly effective treatment, since, despite efforts to improve outcomes, the three-year survival rate for patients with metastatic or recurrent disease is below 20% [2].

Genomic instability and mutagenesis are characteristics of human cancers that can arise from deficient DNA repair processes. One such process, homologous recombination (HR), leads to error-free repair of double strand breaks with homologous strand. BRCAness is a phenotypical trait in tumors resembling the phenotype of tumors with *BRCA1/2* mutations characterized by homologous recombination deficiency (HRD) [3]. The inability of the HR pathway to maintain the genome integrity leads to its instability. OS is characterized by complex genomic variations such as single nucleotide variations and large structural alterations [4].

Recently, we showed that a high percentage of OS displays molecular features resembling BRCAness, i.e. the majority of OS harbors defects in HR repair either due to loss of function in *BRCA1/2* or to mutations in other HR pathway genes [4,5].

Within the concept of synthetic lethality, PARP inhibitors (PARPi) trap PARP1/DNA nucleoprotein complexes and stall the replication fork advancement [6,7]. This induces a DNA damage response and HR repair, involving *BRCA1/2* and “BRCAness” proteins, as the optimal DNA repair process for repairing and restarting replication forks. However, in the absence of functional HR repair, cells use DNA non-homologous repair processes which potentially induce large-scale genomic rearrangements leading to tumor cell death [8]. Tumors exhibiting BRCAness (BRCAness-positive) were shown to be sensitive to PARPi. Development of cytotoxic therapies inducing programmed cell death is an important component of antitumor treatment.

There are several methods to assess BRCAness based on genomic large-scale rearrangements or transcriptional profiles [9–12]. The myChoice method is based on so called genomic scars, i.e. calculating the HRD score by summing up 3 different indicators of genomic instability, namely, large-scale state transition (LST), telomeric allelic imbalance (TAI) and loss of heterozygosity (LOH) [13–15]. Telli *et al*. evaluated the combined HRD score, defined as the unweighted numeric sum of LOH, TAI, and LST [9]. They also tested the predictive power of the HRD score threshold derived by analyzing HRD scores in a training cohort of breast and ovarian tumors with known *BRCA1/2* status. Tumors with HRD score ≥ 42 indicated HR deficiency, the patients were more likely to respond to platinum-containing therapy [9]. Another approach uses mutational signatures derived from single nucleotide variation patterns where a distinctive signature indicates BRCAness in the sample [16].

Several types of methods were developed to assess the real-time status of HRD. Several gene expression profiling (GEP) panels focusing not only on HR but also on cell cycle and DNA repair were developed. For example, a 93-gene GEP panel for predicting the response to platinum therapy in ovarian cancer was established [17]. None of the GEP panels were evaluated exclusively in the context of PARPi susceptibility. In fact, one GEP was evaluated with a chemotherapy drug, paclitaxel, with or without concurrent PARPi (veliparib) or cisplatin administration [18]. Another GEP was retrospectively evaluated on patients treated with platinum-based chemotherapy data where a molecular score was generated based on the expression of 23 genes involved in the platinum-induced DNA-damage repair pathways [17].

To obtain real-time HRD readout without defining the underlying cause of HRD and constructing a GEP panel, a functional *ex vivo* test was developed for breast and ovarian cancers utilizing the ability of crucial HR protein RAD51 to form detectable foci at the double strand break sites after cell irradiation of fresh tumor biopsies and primary cells [19–21]. Still, this test is cell-cycle dependent and requires fresh samples [19]. Thus, the best method to assess BRCAness and to predict response to PARPi in clinical setting has yet to be defined.

Predictive biomarkers will assist in stratification of patients most likely to benefit from PARPi and will channel patients with HR-proficient tumors into non-PARPi treatments, thus minimizing side effects.

In this study we developed a new BRCAness classifier based on DNA methylation patterns predicting BRCAness in OS. Gene expression analysis identified HR signatures corroborating the presence of BRCAness in OS and providing an opportunity to develop an OS GEP panel for predicting PARPi susceptibility. This approach can be extended to other cancers characterized by BRCAness with genome instability.

## Materials and methods

### Ethics statement

Ethics committee approval for conducting the study, use of consent forms and scientific evaluation of the data were obtained from Heidelberg University Hospital’s review board. The approval was granted by the named board (S-502/2013). All patients or their legal guardian signed a written informed consent.

### Tissue collection and DNA extraction

All next generation sequencing (NGS) and DNA-methylation array data were generated and obtained within the scope of the INdividualized Therapy FOr Relapsed Malignancies in Childhood (INFORM) project [22]. Here is a brief description of the INFORM methodology including OS, described in detail in [22].Only patients with relapsed or refractory osteosarcoma between the ages 2–30 were included in the study. Based on histological assessment the content of tumor in the tumor material must be higher than 40%. Fresh frozen samples of tumor material and of its matching control were quality assessed before their molecular analysis.

### DNA sequencing, quality control and read alignment

For the whole-exome sequencing (WES) the paired-end libraries were prepared with the Agilent SureSelectXT Human V5 kit. Low-coverage whole-genome sequencing (lcWGS) was prepared with the exclusion of the enrichment step in SureSelectXT Human V5 kit. RNA-Seq paired-end 100 bp from poly(A)+ RNA was obtained with Illumina TruSeq RNA Kit v2 on an Illumina HiSeq2500 only tumor reverse DNA library. The mean coverage WES was 165-fold for the tumor and 117-fold for the control, WGS coverage was 3.4-fold for the tumor and 2.5-fold for the control. Average number of RNA sequencing reads was 135 million reads.

Quality control, trimming and filtering of FASTQ-files was done with PRINSEQ (ver.0.20.4) [23].Reads were aligned in SAM format with the BWA-MEM algorithm (bwa ver. 0.7.16a) [24]. A full 1000genomes phase2 reference genome sequence *hs37d5* was used for alignment. Sam-files were converted to bam-files, sorted by coordinates and PCR duplicates were removed with the MarkDuplicate subprogram of the Picard software suite (ver. 2.16.0) [25].Bam-files were indexed with samtools [26]. For the final indel realignment, the Genome Analysis Toolkit module (ver.3.8) (GATK) was used [27].

### Genomic scars’ method

Based on the previous studies, NGS-based assay myChoiceHRD (Myriad Genetics) was developed to estimate the degree of so called genomic scar HRD score, i.e. a score combined of counts of specific genomic events such as loss of heterozigosity (LOH), telomeric allelic imbalance (TAI), large-scale transitions (LST) and *BRCA1/2* sequences. HRD-LOH score is represented by the number of LOH regions longer than 15 Mb [15].HRD-TAI is a number of allelic imbalance regions extending to subtelomeres not crossing the centromere and longer than 11 Mb [9,13]. The HRD-LST score was defined as the number of break points between regions longer than 10 Mb after filtering out regions shorter than 3 Mb [14]. Furthermore, the HRD-LST score cutoff between intact and deficient samples was modified based on the samples’ ploidy [28]. myChoiceHRD represents the sum of these three scores, i.e. HRD-LOH, HRD-TAI and HRD-LST. In order to estimate the HRD score in an initial set of 22 OS INFORM samples, genomic scarring events were counted manually, relying on human visualization via Nexus CN software (BioDiscovery, Inc) and the myChoice score was calculated by summing up HRD-LOH, HRD-TAI and HRD-LST events. XY chromosomes’ genomic scarring events were omitted.

### Automation of HRD score assessment with NexusCN and Control-FREEC/HRDtools

In order to automate and remove the human interpretation of genomic scarring events which might cause significant fluctuations in HRD scoring, we employed the NexusCN feature *Percent of Genome Changed* (PGC) which shows what percent of the genome has aberrations. The filter data options were set as follows: *remove gains* smaller than 10000 kb, *remove losses* smaller than 10000 kb, *remove all allelic imbalance calls*, *remove all LOH calls*. Since we use only lcWGS, TAI and LOH calls were not considered.

To calculate PGC with open source software we used Control-FREEC (version 11.4.3) and HRDtools (version 0.0.1) software [29]. Control-FREEC was developed as a tool for detection and enumeration of copy-number alterations (CNA), TAI and LOH using NGS data [30]. Briefly, from tumor-control bam-files we generated pileup-files using samtools mpileup [26]. Then, we ran Control-FREEC with pileup-files as an input. We post-process Control-FREEC output to make it compatible with HRDtools. We modified HRDtools so that it can closely follow myChoice HRD genomic size filtering (LOH = 15 Mb, LST = 10 Mb, TAI = 11 Mb).Modified HRDtools provided in the output the width of CNA, TAI and LOH. Dividing summed up width by the length of the haploid genome (3,234,834,689—bases GRCh37.p13) we obtained a PGC score.XY chromosomes were not considered for the overall PGC score.

### DNA-methylation arrays

The tumor DNA-methylation profile was assessed with Illumina HumanMethylation450 and Epic BeadChip (482,421/850,000 CpG sites, respectfully) according to the manufacturer’s instructions. There were 43 DNA methylation arrays of OS generated within the INFORM project. Analysis and processing of 450k and Epic arrays were done with an integrated analysis pipeline, R package ChAMP, which includes filtering low quality probes based on detection P-values, chromosomal location and presence of single nucleotide polymorphisms in the probe sequence. For filtering and processing the default values were kept [31]. In order to utilize both 450k and Epic arrays we used the function convertArray from R package minfi [32]. This function converts EPIC into 450k arrays by dropping probes that differ between two array types (M*odified champ*.*load function from package ChAMP with incorporated convertArray method in Supplement)*. After obtaining beta-values, defined as the ratio of a probe fluorescence intensity over the overall intensity, beta-values were further normalized with Beta MIxture Quantile dilation (BMIQ) adjusting the beta-values of type II design probes into a statistical distribution characteristic of type I probes. BMIQ was suggested as the preferred methods for case—control, tumor—normal or, in our case, BRCAness negative—positive dataset processing. BMIQ was shown to be more effective at removing unwanted technical bias than other normalization methods [33]. Methylation probes were annotated with HumanMethylation450 v1.2 manifest (Illumina).

### Setting the threshold for BRCAness positive samples

A tumor with a myChoice score above 42 was considered HRD/BRCAness-positive [9]. NexusCN PGC score above 32, FREEC-HRDtools PGC score above 28 were considered as BRCAness-positive after matching PGC scores to myChoice scores. Throughout this study we used the NexusCN PGC score, as the original calculation of the myChoice score for OS samples was done with NexusCN assistance. 43 OS INFORM samples were grouped to two classes of 26 and 17 samples, i.e. BRCAness-positive and BRCAness-negative group, respectively, based on the threshold of a NexusCN PGC score of 32 which corresponds to a myChoiceHRD score of 42.

### Feature selection and testing class structure

First, variances of each probe were calculated within the BRCA positive (var_pos) and negative (var_neg) training group. var_all was computed for each sample regardless of the grouping. It can be represented as Δ-variances = var_all–(var_pos + var_neg) [34]. Probes were ranked in descending order according to Δ-variances and the top 2,000 probes (features) were selected for further random forest (RF) training and validation.

Since the number of predictors was considerably larger than the number of samples and to avoid *importance* bias caused by multiple highly correlated probes [35], only one probe was retained from probes correlated above 0.8. As a result, only 2.7% (54 probes) of the original probe (feature) set of 2000 probes remained. We used 41 sample set and uncorrelated predictors further in the training and testing of the RF. Correlated probes were included in the biological functional analysis but excluded from the RF training and validation stages.

To test whether we found a real class structure in the data, the corresponding null distribution was estimated by permuting the labels in the data and by enumerating the number of positive Δ-variances. Our grouping based on PGC and HRD scoring had 3,685 positive Δ-variances. The labels were permuted 1000 times and every time the number of probes with positive Δ-variances was counted. In 30 out of 1000 runs only a single probe had positive Δ-variance, the rest of the runs had zero positive Δ-variance probes. This result supports our approach to OS sample grouping and our assumption that only probes with positive Δ-variances are informative.

### Random forest

To identify the predictive set of probes underlying BRCAness we utilized a machine learning approach, random forests, using the R randomForest package (version 4.6–14) in R [36–38]. For the most part, we followed a predictive modelling approach and the R script described in Valletta *et al*. with parameters modified for our dataset [39]. For RF training our unbalanced set of samples was stratified for class balancing. As input variables for our models we used the intensity of methylation probes. The response variable, i.e. the outcome to be predicted, was sample assignment either to BRCAness-positive (’Yes’) or to BRCAness-negative group (’No’) as it was defined by the NexusCN PGC score. The number of trees was set to 10000, 32 variables were tried at each node and the minimum node size was set to 3. The tree depth limit was not set. Uncorrelated predictors were run through a predictive modelling pipeline, where an additional statistical significance of selected features was computed with a modified mProbes/xRF feature selection method [39–41]. However, all uncorrelated probes showed high predictive power by having high statistical significance after introducing predictors with randomly shuffled values. All uncorrelated predictors were used in RF for training and testing.

### Accuracy evaluation

To assess model predictive accuracy, we used RF internal error estimation, i.e. out-of-bag (OOB) error and the receiver operating characteristic (ROC) curve [37].The feature selection algorithm was used internally in a five-fold cross-validation loop. The OOB error confusion matrix generated by RF has been used for accuracy estimation.The area under the ROC curve (AUC) was computed for each fold. The average AUC was computed with combined TP/FP events generated in each fold [39]. The 5-fold CV RF models were not kept. The final RF model was trained on the entire dataset of 41 samples. Accuracy was reported as (TP+TN) / (TP + TN + FP + FN) x 100. Combined accuracy was computed when TP, FP, TN and FN events obtained in each fold of CV were combined together, e.g. if TP1 is TP of fold 1 CV, then TP1 + TP2 + TP3 + TP4 + TP5 = TP_comb. The average (combined) AUC was computed with TP_comb and FP_comb events. The heatmaps and dendrograms were generated with unsupervised hierarchical clustering using Euclidean distance and Ward.D2 clustering method.All plots and statistical tests were performed with statistical software R [38].

### RNA-Seq

For alignment and generation of bam-files, a splice-aware RNA-Seq aligner, STAR (ver. 2.7.1a), has been used with default settings [42]. The number of reads mapping to genes (features) from bam-files were calculated with htseq-count which is a part of the HTSeq framework (ver. 0.9.1) [43].Non-unique reads, secondary, supplementary alignments and reads overlapping more than one feature were discarded. Gene reference GTF file was obtained from UCSC/hg19 annotation archive. Read counts were transformed using the DESeq2 framework with regularized logarithm (rlog). rlog transformation generates data on a log2 scale normalized to library size. DESeq2 was used to obtain set of DE genes [44]. Normally, several biological replicates are required for DESeq to identify genes differentially expressed above biological noise. Here, we treated BRCAness-positive and BRCAness-negative OS samples similarly to biological replicates for the purpose of capturing profoundly expressed genes between two classes. After all, most of the changes in gene expression between these two classes can be explained by the genetic heterogeneity of samples rather than random biological noise. Thus, BRCAness-positive and BRCAness-negative samples were grouped conditional on the presence or the absence of BRCAness characteristics. Gene differential expression was considered statistically significant with fdr < 0.05.

### Gene set enrichment analysis

A pre-ranked list of all genes based on logFC sign and 1/p-val was analyzed by the gene set enrichment analysis tool (GSEA ver. 4.0.2) [45,46]. The enrichment was evaluated using Molecular Signatures database (MSigDB) sets, namely Hallmark set, KEGG (MSigDB ver.7.0) [47–49]. Enrichment was considered significant if the gene set family wise error rate (FWER) was less than 0.05. GSEA particular settings were set to *classic* enrichment statistics, *no normalization* mode, *mean of probes*. Differentially expressed upregulated and downregulated sets of genes with fdr < 0.05 were uploaded to the gene set enrichment web-tool Enrichr [50]. Enrichment was considered significant if the gene expression signature had fdr < 0.05.

## Results

### HRD score evaluation with NexusCN and Control-FREEC/HRDtools

We simplified the computation of HRD scores with PGC in order to separate samples to BRCAness-positive and BRCAness-negative groups for further RF training and validation. We observed a strong correlation of PGC with canonical HRD (LST + TAI + LOH). Spearman rho correlation between the myChoice HRD score and the NexusCN PGC, and between the myChoice HRD score and the FREEC-HRDtools PGC were 0.92 (p-val = 1.3e-09) and 0.86 (p-val = 3.2e-07), respectively. Spearman rho correlation between the FREEC-HRDtools PGC and the Nexus CN PGC was 0.91 (p < 2.2e-16) (Fig A in S1 File). Since the correlation between the myChoice HRD score and the NexusCN PGC was higher and did not require human intervention, we continued with the NexusCN PGC as a BRCAness score replacing the myChoice HRD score except validating independent test set where we used the FREEC-HRDtools PGC, since the NexusCN software was no longer available. Overall, we showed that the PGC approach is suitable for separation of samples to two groups. However, using PGC as an alternative to the canonical HRD score requires testing it on a large number of tumor samples, such as ovarian cancer which was initially used to develop and test HRD scoring.

### Analysis and filtering of sample set

**Fig 1:**
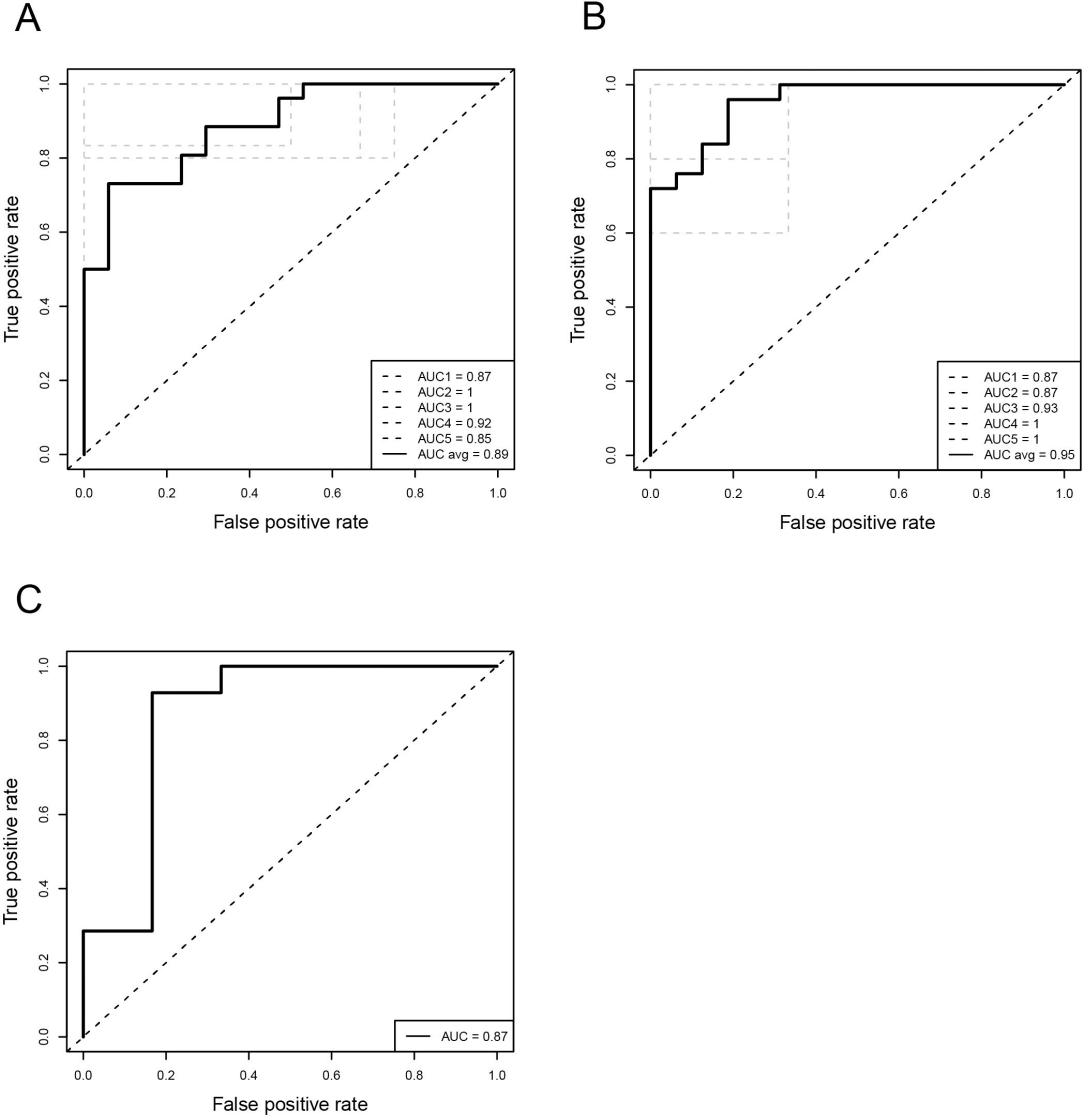
**ROC curves for each cross validation fold (dashed-black line) and the mean ROC curve (solid black line) for random forest models** fitted (A) to 43 samples and (B) to 41 samples where two samples, HD_00J and I047_005 were removed (the dashed diagonal line represent AUC = 0.5, i.e. classification at random). (C) ROC curve for independent validation test (AUC = 0.87) (n = 20).

First, we analyzed a full dataset of 43 samples where methylation probes were ranked in the order of decrease of Δ-variance and the top 2000 probes were selected for further training and testing of the RF machine learning algorithm in order to build a model discriminating between BRCAness-positive and BRCAness-negative classes of OS samples. The results of this model testing and training evaluated with ROC curves and confusion matrices are shown (Fig 1A and Fig B in S1 File and Table 1).

**Table 1:** Confusion matrices of each cross validation and average fold for random forest models.

		Fold 1		Fold 2		Fold 3		Fold 4		Fold 5		Fold Average	
		Predicted		Predicted		Predicted		Predicted		Predicted		Predicted	
RF models	Actual	No	Yes	No	Yes	No	Yes	No	Yes	No	Yes	No	Yes
^a^fitted to 41 samples	No	2	1	2	1	2	1	4	0	3	0	13 (81%)	3 (19%)
	Yes	1	4	2	3	0	5	0	5	0	5	3 (12%)	22 (88%)
^b^fitted to 43 samples	No	1	2	3	0	3	0	2	2	1	3	10 (59%)	7 (41%)
	Yes	0	5	1	4	1	4	1	5	0	5	3 (12%)	23 (88%)

^a^Accuracy = (22+13)/(22+13+3+3) x100 = 85% (samples I047_005 and HD_00J removed)

^b^Accuracy = (23+10)/(22+13+3+7) x100 = 77%

**Fig 2:**
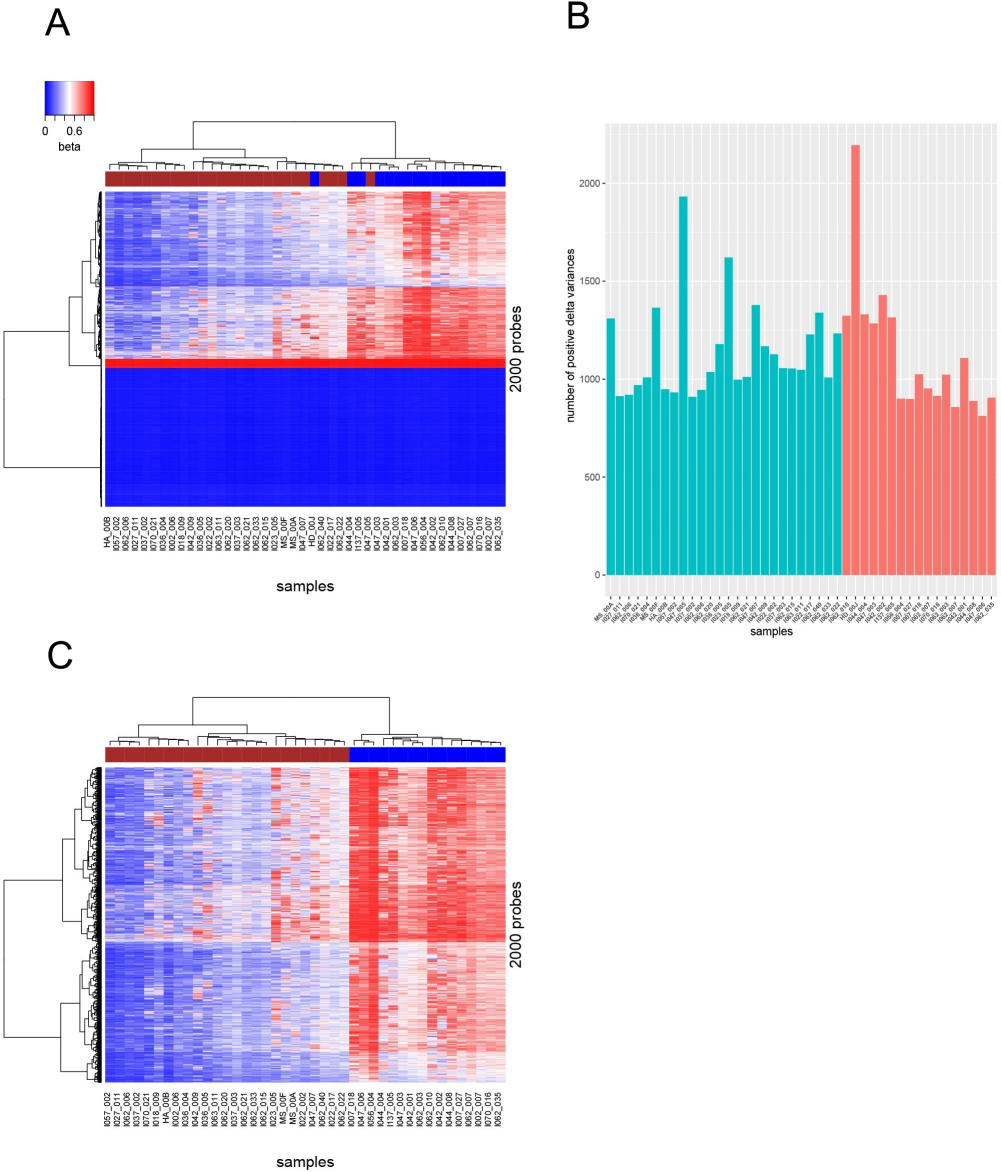
**Initial unsupervised hierarchical clustering of 2000 probes** (A) in all available 43 samples and (C) in 41 sample set after removal of two samples (HD_00J and I047_005). Note that these two samples were clustered incorrectly. Lower part in heatmap (A) shows probes with very low intensities across all samples yielding delta variances with negative values. Brown band: BRCAness-positive, blue band: BRCAness negative samples. (B) Barplot shows the number of positive Δ-variances re-computed after excluding each sample consequently from 43 sample set. Red—BRCAness negative, green—positive samples, Y-axe—number of positive Δ-variances. Subsequent removal of HD_00J and I047_005 gives the largest increase in the number of positive Δ-variances.

The inherited noisiness of the OS samples provided negative Δ-variances for the majority of probes. In the 43 sample set we have only 1,055 probes with positive Δ-variance (~0.25%) and 426,520 probes with negative Δ-variances. With this settings taking the top 2000 probes led to misclassification of two samples at unsupervised hierarchical clustering (Fig 2A).

In order to maximize the number of informative predictors with positive Δ-variances, without significantly reducing the number of samples, we computed the number of probes with positive Δ-variances by removing one sample at a time from the 43 sample set and re-calculating the number of positive variances (Fig 2B and Table A in S1 File). Subsequent removal of two samples, I047_005 and HD_00J, in BRCAness positive and in BRCAness negative group, correspondingly showed the largest number of probes with positive Δ-variances. These two samples were the ones misclassified at unsupervised hierarchical clustering (Fig 2A).

Therefore, we assumed that the negative Δ-variances decreased the accuracy of the prediction and only probes with positive Δ-variances were informative. After removal of samples I047_005 and HD_00J, unsupervised hierarchical clustering correctly grouped 41 samples and the number of probes with positive Δ-variances increased to 3685 (Fig 2C). The accuracy estimated by AUC and confusion matrices improved for the 41 OS sample set shown in Fig 1B and Fig B in S1 File and Table 1 as well as grouping in hierarchical clustering (Fig 2C).

The OOB error evaluates model performance on data not participating in the training of RF.However, due to selection bias the OOB error computed on a model fitted on the features selected just once might lead to overfitting and yield over-optimistic error estimation [51]. To avoid this bias, a Δ-variance feature selection algorithm was used internally in a five-fold cross-validation loop [39]. Within each fold, feature selection was conducted *de novo*. The model fitted using the selected features anew each time and was tested against the fold set aside.

**Fig 3:**
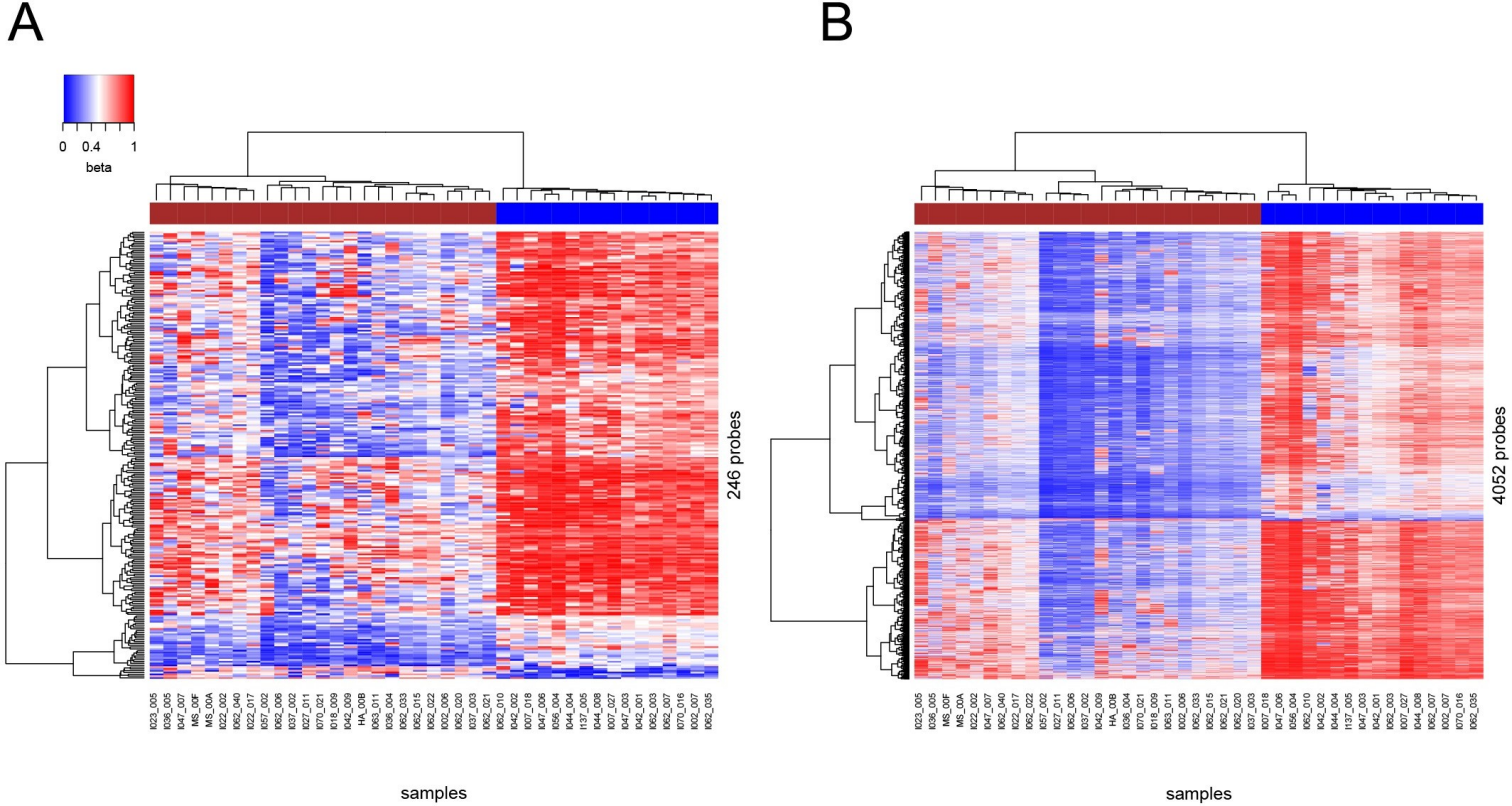
Unsupervised hierarchical clustering of samples and probes in 41 sample set. (A) 246 probes identified as important non-correlated predictors combined after 5-fold cross validation and (B) 4052 probes correlated to and including 246 probes discriminate between BRCAness-positive and BRCAness-negative samples. BRCAness-positive and BRCAness-negative samples indicated by brown and blue horizontal bars at the top, correspondingly. Please note, for the final RF model, used for validation with independent test set (n = 20), all 41 samples were used as one training set. This set yielded 54 uncorrelated probes out of 2000 probes.

During a five-fold cross-validation loop the feature selection, i.e. selection of 2000 top probes was performed independently according to the *filter* method as opposed to the *wrapper* method. The *filter* method picks up inherent properties of the features based on univariate statistics [52]. Since 1/5 of the samples were excluded from training and left for testing purposes, i.e. not participating in computing of Δ-variances, the set of 2000 probes was fluctuating after each iteration. The 41 sample set showed improved accuracy evaluated with AUC and confusion matrix (Fig 1B and Fig B in S1 File and Table 1). Unsupervised hierarchical clustering of uncorrelated 246 probes/predictors, extracted after each cross-validation loop in the course of the feature selection and, subsequently, merged into a non-redundant set, channeled OS samples to corresponding BRCAness-positive and BRCAness-negative groups (Fig 3A). Also, correct grouping was observed when 4052 probes correlated to and including these 246 probes (rho > 0.8), were merged (Fig 3B) and subjected to unsupervised hierarchical clustering. A pipeline for data processing, RF classifier training and validation is shown in Fig C in S1 File.

The ROC curve is a plot visualizing the true positive (TP) rate against the false positive (FP) rate at various threshold settings. The average of the area under the ROC curve (AUC) across all 5 CV folds with 43 samples was 0.88 (Fig 1A). However, after removing two samples, I047_005 and HD_00J, the average AUC increased to 0.95 (Fig 1B). Also, the combined accuracy after 5-fold CV increased from 77% to 85% in 41 sample set compared to 43 sample set (Table 1). Keeping in mind that maximum AUC is 1.0 when prediction is 100% accurate and, AUC is 0.5 with random prediction, the average AUC of the DNA-methylation based RF classifier 0.95 demonstrates accurate prediction of BRCAness in OS samples. Additionally, the RF classifier trained on 41 OS samples was validated with an independent OS test set (Fig 1C).

The arrangement of samples in order of increase of PGC, the measure of BRCAness obtained from genomic NGS data, shows a pattern of probe intensity decrease with increase of PGC (Fig D in S1 File). This suggests that gene expression is repressed through higher DNA-methylation in BRCAness negative samples and, conversely, in BRCAness positive samples expression of certain genes are upregulated. However, we cannot exclude the possibility that the reason for lower DNA methylation signal in BRCAness positive samples was simply because of massive genome alterations affecting DNA sites of methylation.

In line with a standard operating procedure for RF, we assigned a fraction of RF votes as a measure of DNA repair defect, where a fraction of RF votes > 0.5 classifies a sample as BRCAness positive.

### RNA-Seq sample selection and differential expression analysis

**Fig 4:**
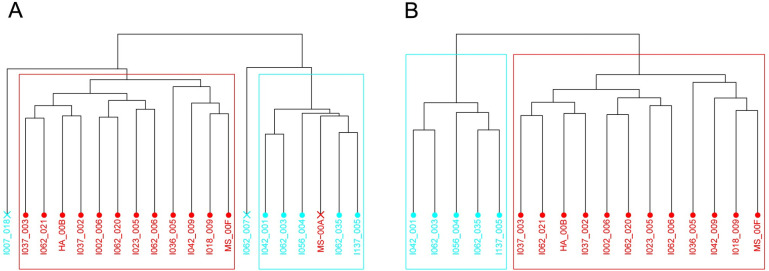
**Dendrogram showing the results of our hierarchical cluster analysis** of (A) 20 RNA-Seq samples selected from two thirds top and bottom of the OS sample list arranged according to PGC score; and (B) 17 remained RNA-Seq samples used for DE analysis and GSEA. BRCAness-positive samples are denoted by red color and BRCAness-negative by cyan color. Leaves representing excluded samples are denoted with ’x’. The demarcated boxes in red and cyan represent two groups obtained by cutting the dendrogram at even height and used further in DGE and GSEA. The height of a group is defined by Ward’s method.

In order to assess heterogeneity of RNA-Seq samples, Principle Component Analysis (PCA) was applied (Fig E in S1 File). The overlap of ellipses denoting the confidence interval showed poor separation of the two groups. As we expected, the two samples, HD_00J and I047_007 (already removed in RF), were far away from their corresponding clusters, and for this reason these RNA-Seq samples were excluded from subsequent consideration. Out of 41 OS samples used in methylation classifier, 31 samples had RNA-Seq data. To avoid samples in the "grey zone", i.e. around the border line separating BRCAness-negative group from BRCAness-positive group, we selected 2/3 of the top samples with the highest score from the BRCAness-positive group and 2/3 of the bottom samples with the lowest score from the BRCAness-negative group ranked according to Nexus PGC and used their corresponding RNA-Seq data if they were available. This approach excludes overlapping and border line samples. This approach yielded 13 BRCAness-positive and 7 BRCAness-negative RNA-Seq samples. To assess the heterogeneity of RNA-Seq samples, unsupervised hierarchical clustering was applied (Fig 4A). It shows each individual RNA-seq sample (leaf) joined by the branches. The height represents the distance value from the input Euclidean distance matrix. One can consider the first split in the dendrogram to be the two largest distinct groups, with more groups forming at each subsequent split down the dendrogram. In Fig 4, the demarcated boxes in red and cyan represent two groups obtained by cutting the dendrogram at even height. Moving top-down, the group in the red box and I007_018 are separated from the rest of the groups at the top level. Thus, it is expected to find most difference between sets of samples separated at the top level. Considering the right side of the first split, the outlier I007_018 is separated from the group of samples in the red box and hence it is not included in the final set of RNA-seq samples. A similar rationale was applied to I062_007, even though I062_007 belongs to the same class as the samples in the cyan box. BRCAness-positive RNA-Seq MS-00A was excluded from the stretch of BRCAness-negative samples. Unsupervised clustering and PCA of the remaining 17 RNA-Seq samples, 12 BRCAness-positive and 5 BRCAness-negative, showed that two clusters were well-separated (Fig 4B and Fig E in S1 File). Subsequently, these 17 RNA-Seq samples were used for differential expression (DE) and gene set enrichment analyses (GSEA).

There were 1,528 significantly DE genes consisting of 449 genes upregulated in BRCAness-positive group and 1,079 genes downregulated. Among the genes upregulated in BRCAness-positive versus BRCAness-negative OS tumors were genes important in DNA replication, DNA repair, cell cycle regulation such as *BLM*, *BRCA1*, *EME1*, *ERCC1*, *EYA1*, *FANCB*, *H2AX* (aka *H2AFX*), *HIST1H2BC*, *HIST1H2BD*, *HIST1H2BN*, *LIG3*, *POLD2*, *POLH*, *RPA3*, *SMUG1*, *TIPIN*, *TOP3A*, *TIMELESS*. Of note, the upregulated histone H2AX was extensively studied as a DNA damage response factor. The change between γH2AX dephosphorylation and H2AX phosphorylation marks the switch between cell repair and apoptotic responses, correspondingly [53]. γH2AX foci formation correlates with a double strand break induced by irradiation exposure [54].

The majority of the gene expression signatures downregulated in BRCAness-positive versus BRCAness-negative OS tumors were those associated with the immune response indicating the level of activity of the immune cells in the OS microenvironment. The genes related to adaptive immunity such as T cell receptor genes (CD3E, CD3D, CD3G) and B cell genes (CD79A, CD83) as well as genes of the major histocompatibility complex (MHC) such as *HLA-DOB*, *HLA-DQA1*, *HLA-DQB1* were significantly downregulated in BRCAness-positive OS tumors. The NF-kB, composed of a family of genes (NFKB1, NFKB2, RELB, REL), an important transcription factor related to T-cell activation and cytokines expression, was significantly downregulated or showed strong tendency towards downregulation in BRCAness-positive versus BRCAness-negative OS tumors. Expression of genes specific to cytotoxic activities mediated by T cells, such as CXCR3, PRF1, KLRF1, GZMH, GZMK was also significantly reduced in BRCAness-positive OS tumors as well as expression of the genes associated with immune response and proinflammatory cytokines (IL1A, IL18, CCL11, CCL22, CCL13) (Table A in S2 File).

### Gene set enrichment analysis

The Broad Institute GSEA tool reported gene sets with a positive enrichment score, i.e. gene sets that display enrichment at the top of the pre-ranked list, were *hallmark dna repair*, *kegg dna replication*, *kegg mismatch repair*, *kegg homologous recombination* (Table 2 and Fig F in S1 File). Genes contributing most to the enrichment results in gene sets related to DNA repair and damage tolerance are listed in Table B in S1 File. The second portion of the GSEA report showed results for gene sets with a negative enrichment score, i.e. gene sets that display enrichment at the bottom of the pre-ranked list. Those signatures included *hallmark allograft rejection*, *kegg asthma*, *kegg graft versus host disease*, *kegg allograft rejection* (Table 2 and Fig G in S1 File). Genes contributing most to the enrichment results in gene sets related to immune system disease category are listed in Table C in S1 File. *Asthma*, *allograft rejection*, and *graft versus host disease* signatures reflect downregulation of MHC classes I and II which in turn reduces responsiveness of anti-tumor immune cells and is typical tumor evasion of the immune response [55,56] (GSEA results in the supplement).

**Table 2:** Enrichment results for gene sets.

GSEA	NAME	SIZE	ES	NES	NOM p-val	FDR q-val	FWER p-val
Positive enrichment score	KEGG_DNA_REPLICATION	30	0.639	0.639	0.000	0.000	0.000
	KEGG_MISMATCH_REPAIR	22	0.490	0.490	0.000	0.001	0.003
	KEGG_HOMOLOGOUS_RECOMBINATION	26	0.482	0.482	0.000	0.001	0.004
	KEGG_ONE_CARBON_POOL_BY_FOLATE	17	0.427	0.427	0.001	0.004	0.023
	KEGG_RNA_POLYMERASE	28	0.426	0.426	0.000	0.003	0.023
Negative enrichment score	KEGG_ASTHMA	23	0.705	-0.705	0.000	0.000	0.000
	KEGG_GRAFT_VERSUS_HOST_DISEASE	36	0.674	-0.674	0.000	0.000	0.000
	KEGG_ALLOGRAFT_REJECTION	33	0.669	-0.669	0.000	0.000	0.000
	KEGG_TYPE_I_DIABETES_MELLITUS	39	0.602	-0.602	0.000	0.000	0.000
	KEGG_AUTOIMMUNE_THYROID_DISEASE	33	0.578	-0.578	0.000	0.000	0.000
	KEGG_HEMATOPOIETIC_CELL_LINEAGE	80	0.549	-0.549	0.000	0.000	0.000
	KEGG_RENIN_ANGIOTENSIN_SYSTEM	16	0.547	-0.547	0.000	0.000	0.000
	KEGG_INTESTINAL_IMMUNE_NETWORK_FO	43	0.492	-0.492	0.000	0.001	0.003
	KEGG_COMPLEMENT_AND_COAGULATION_	61	0.487	-0.487	0.000	0.001	0.004
	KEGG_CELL_ADHESION_MOLECULES_CAMS	122	0.468	-0.468	0.000	0.001	0.005
	KEGG_PRIMARY_IMMUNODEFICIENCY	34	0.453	-0.453	0.000	0.001	0.009
	KEGG_LINOLEIC_ACID_METABOLISM	22	0.428	-0.428	0.001	0.002	0.021
	KEGG_LEISHMANIA_INFECTION	69	0.427	-0.427	0.000	0.002	0.023

As additional GSEA we applied the web-tool Enrichr (https://amp.pharm.mssm.edu/Enrichr/) where statistically significant upregulated and downregulated genes were uploaded as separate gene sets. Enrichr with upregulated genes and KEGG database reported Fanconi anemia pathway and HR, both pathways share most of genes such as *BRCA1*, *POLD2*.Enrichr with the Reactome database reported multiple signatures related to HR and DNA-repair such as homology directed repair, DNA double-strand break repair. For the set of downregulated genes Enrichr reported MHC II genes in graft-versus-host-disease, allograft rejection and in other signatures (Enrichr results in the supplement).

### Validation of classifier with independent test set

Already after training BRCAness classifier, we obtained OS samples (n = 20) through INFORM ongoing collection of samples with relapsed or refractory osteosarcoma. This test set was used for validation of BRCAness classifier. Samples were separated to BRCAness-positive (n = 14) and BRCAness-negative (n = 6) groups based on FREEC-HRDtools PGC score. New methylation data arrays were normalized together with training set. Subsequently, trained model was applied to test set for classification. Only single sample in each group was misclassified (Table 3). The validation with independent OS set resulted in AUC of 0.87, accuracy of 0.9, sensitivity and specificity of 0.93 and 0.83, correspondingly (Fig 1C). Differential expression analysis was performed with the RNA-Seq test set where all samples classified as BRCAness-positive (n = 14) and BRCAness-negative (n = 6) were grouped conditional on the presence or the absence of BRCAness characteristics, tested for DE, ranked, and subsequently analyzed for pathway enrichment. For the set of upregulated genes Reactome database reported multiple signatures related to HR and DNA-repair such as *homologous DNA-pairing and strand exchange*, *homology directed repair through homologous recombination*, *DNA-double strand break repair* and *homology directed repair* (Table D in S1 File). KEGG database reported *cell cycle*, *dna replication* signatures as well as signatures related to DNA-repair, specifically, *mismatch repair* (fdr < 0.05), *homologous recombination repair* (fdr < 0.05) for the set of upregulated genes. For the set of downregulated genes KEGG reported MHC II genes in graft-versus-host-disease, allograft rejection and in other signatures including apoptosis (Table E in S1 File). Matching signatures were observed in training set GSEA.

**Table 3:** Test set confusion matrix.

^a^ Test set	Actual	Predicted	
		No	Yes
n = 20	No	5(83%)	1(17%)
	Yes	1(7%)	13(93%)

^a^Accuracy = (5+13)/20 x100 = 90%; Sensitivity = 13/(13+1)x100 = 93%; Specificity = 5/(5+1)x100 = 83%.

## Discussion

As OS is the most common primary malignant bone tumor in adolescence with a low rate survival for recurrent and metastatic disease in patients there must be efficient treatment of relapses in place and, ideally, a reliable approach to predict if the treatment will be successful [1,2]. OS shows complex patterns of genomic variations with a high amount of inter- and intratumoral heterogeneity, probably responsible for treatment failure in a subgroup of patients [57]. The structural variations in OS affect BRCA1/2 and their binding partners which are involved in HR repair [4]. Mutations of these binding partners can be functionally equivalent to BRCA1/2 mutations and also result in HRD. Thus, loss of function in genes involved in HR repair can lead to BRCAness resulting in increased vulnerability to PARP1 inhibition [3]. Earlier, ’BRCAness’ tumors, i.e. tumors with traits resembling BRCA mutant tumors were identified in breast and ovarian cancers [58,59]. These tumors were shown to respond to therapy with PARPi due to HRD [60,61]. It was demonstrated by Kovac *et al*. that the majority of OS fulfills the criteria for BRCAness and that OS cell lines showing these characteristics respond to PARPi [4].

In order to measure BRCAness, several genomic tests have been developed using breast and ovarian cancer datasets [9,10]. The myChoice HRD composite score utilized the genomic scars’ method based on large genomic rearrangements, namely HRD-LOH, HRD-LST and HRD-TAI [15]. There is also a mutational signature method built on single nucleotide variation patterns [16].The weakness of the genomic scars’ method is that it does not necessarily reflect the actual BRCAness status of the tumor, nevertheless it is widely accepted in clinical practice. Also, the HRDetect method based on high coverage whole genome sequencing employs both mutational signatures and genomic alterations [29,62].

Several approaches for detecting the real-time status of HRD were developed. Transcriptional profiles with GEP panels focused on HR were evaluated in ovarian and breast cancer for sensitivity to platinum therapy, but not specifically in relation to PARPi susceptibility [17]. In breast cancer, the GEP panel profile included genes found not only in signatures of DNA replication, repair but also in signatures of cell cycle, cellular assembly and maintenance as well as metabolic signatures [18].

In another approach, primary cancer cells were subjected to ionizing radiation-induced damage and the HR response was accessed by the number of RAD51 nuclear foci. Though these *ex vivo* tests for ovarian and breast cancers can determine real-time HR status, they are highly cell-cycle dependent and require fresh samples [19,20].

This study reports a DNA methylation-based classifier trained on samples classified with the genomic scars/myChoice method.This classifier detects BRCAness in OS samples with high accuracy and assigns a BRCAness score to the sample. To the best of our knowledge, DNA-methylation data was used for BRCAness detection and BRCAness scoring for the first time.

There are DNA methylation-based RF classifiers developed for different purposes such as classification of cancers, which can be misclassified through histological analysis. Wu et al. developed a genome-wide DNA methylation-based classifier to differentiate between osteosarcoma, Ewing sarcoma, and synovial sarcoma [34]. The other work was aimed at developing a DNA methylation-based classification tool for categorization of soft tissue and bone sarcomas representing a broad range of subtypes and age groups [63]. Another DNA-methylation classifier was implemented to assist in diagnosing brain neoplasms by discriminating between primary and metastatic brain tumors, identifying the tissue of origin of the brain metastases, and discriminating their subtypes [64].

In order to develop the classifier, we used the Random Forest (RF) algorithm. Briefly, the RF classifier belongs to the ensemble-based learning methods. There are training and classification stages in the RF algorithm. In the training stage, multiple decision trees are generated and a bagging algorithm is applied to each tree in the forest. Bagging constantly picks random samples with a replacement from the training set and fits trees to these samples.Each tree is grown without pruning. The classification stage consists of gathering the majority vote across decision trees, i.e. combining predictions from all models. In other words, RF combines the predictions of many decision trees into a single model. We chose RF among others for the following reasons: 1) RF is computationally inexpensive and fast. It was already widely used in various domains of data science; 2) RF is resistant to overfitting because of its voting strategy mentioned above. In contrast, a decision tree classifier might become fitted to the training data together with any associated noise. This would prevent such a model from predicting the independent test data; 3) in RF we can still trace how each feature was contributing to the prediction model. This characteristic of RF can be important for clinical acceptance which often requires a high degree of transparency in terms of how a specific decision is made. This is an advantage of RF over “black-box” decision making such as a neural network algorithm.

Using methylation array probes as predictors has several advantages over using NGS. Microarray platforms have been in use for a longer time than NGS and the methods are more standardized in terms of technology, allowing rapid genome-wide methylation analysis. Other advantages include less complicated and labor-intensive sample preparation and the ability to process a high number of samples. NGS data used as an input for myChoice HRD and PGC scoring is more suitable for discovery purposes. Its analysis requires both tumor and control (normal tissue) data. Obtaining control sample might be problematic for certain cancers. In samples with the low tumor cell content, coverage might be too low to capture genomic rearrangements. Turnaround time can be too long for diagnostic purposes and costs associated with data handling and storage, bioinformatics analysis can be too high. With appropriate equipment, methylation arrays can be processed locally, in the lab, without sending sample to large sequencing center.Usually, clinicians are more comfortable with stable, proven technology, straightforward data analysis and ease of regulatory approval.

Importantly, in this study we demonstrated that 54 probes, i.e. approximately 0.01% of all probes on a 450K DNA-methylation array, were sufficient to predict BRCAness with high accuracy in the test OS set. This shows that the predominant majority of the approximately 450,000 probes are uninformative or redundant for the discrimination of BRCAness in OS. This notion allows scaling down DNA-methylation BRCAness test adjusting it to different lower throughput platforms without deteriorating test accuracy. The scaled-down test of 54 CpG sites does not require array equipment and could be a solution for small diagnostic laboratories providing a much shorter turnaround time [65]. The NGS testing of a small number of CpG sites could offer higher multiplexing and better sequencing depth providing better resolution for some problematic CpG sites. There are several lower throughput technologies that can be used for interrogation of tens to hundreds of CpG sites simultaneously, such as methylation-iPLEX assay which can produce DNA methylation data from a DNA sample in a single workday [66]. With quantitative methylation-specific PCR method one would expect a lower cost per sample per CpG site [67]. Also, droplet digital PCR is considered to be a valuable technology for accurate methylation detection in clinical samples with its higher precision, greater accuracy, and technical simplicity over conventional quantitative PCR [68].

Our results show that the DNA-methylation RF classifier can identify BRCAness in a manner that is not only accurate and time effective but also cost-effective, since processing of methylation arrays for the RF classifier does not require sequencing of control tissue DNA. Although BRCAness in OS was previously shown on the genomic level with HRD score and mutational signatures as well as OS susceptibility to PARPi [4,69], on the gene expression level genome instability signatures including homologous recombination were identified for the first time in OS. The low frequency of OS tumors truly deficient in BRCA1/2, in contrast to breast and ovarian cancers, implies that the increased presence of BRCAness in OS arises from alterations in other genes of the HR pathway, each of which contributes to HRD [4]. Susceptibility of OS cell lines to PARPi broadly correlated with their level of genomic instability, and cells exhibiting high sensitivity to PARPi scored higher in the HRD score, i.e. in a measure of genomic instability [69]. Taking this into account we expect that the degree of DNA-repair defect obtained with the DNA-methylation classifier can be used as a measure of PARPi response.

Currently, our study has several limitations. There is a potential to improve the accuracy of prediction by increasing the number of OS samples and by re-training the RF model using only Illumina Epic arrays with >850,000 CpG probes. In this study, we converted all Epic array probes to Illumina 450K probes. This array has only 450,000 CpG sites throughout the genome because we used both types of arrays. Such a reduction in the number of probes decreases the predictive accuracy of the classifier. Furthermore, this classifier was trained and validated on samples segregated based on the genomic scars’ method, i.e. on large scale genomic rearrangements accumulated over certain time. In this regard genomic scars might exhibit HRD status which does not reflect the current state of BRCAness in tumor. However, the relation between the dynamics of the scars’ accumulation and real-time HRD status was not explored to the best of our knowledge, yet. Nevertheless, the genomic scar method is the most common method of HRD detection commercialized by Myriad Genetics and approved by the U.S. Food and Drug Administration for clinical use. Clearly, the prediction accuracy does not exceed those of the myChoice HRD / NexusCN PGC method since the classifier was trained and evaluated on OS samples separated with the aforementioned method to BRCAness-positive/-negative subsets. Having more advanced methods in hand for categorizing OS samples, e.g. a functional ex vivo test with enough samples for training and evaluation, we could use methylation arrays of corresponding samples for the classifier which offers a straightforward framework. Another possible limitation is that the classifier was trained and validated only with INFORM samples which are relapsed or refractory OS samples. Hence, it is likely that for BRCAness detection in primary OS tumors this classifier should be re-trained and re-validated.

Though, preliminary data on the antitumor activity of DNA-damaging drugs and PARPi in combination showed encouraging results in osteosarcoma [70] as well as in other ongoing clinical studies with PARPi (e.g. NCT03155620, NCT02813135), in the past the response to PARPi was not systematically studied in OS patients. Hence, an unambiguous cut-off score in OS for defining BRCAness was not available. Thus, reproducing the HRD test with the BRCAness threshold of ≥ 42 determined for ovarian cancer seemed to be a reasonable approach to group OS samples to BRCAness-positive and negative ones [9].

We want to emphasize that the degree of BRCAness obtained with our RF classifier should not be considered as an explicit indication to administer PARPi in OS. Nevertheless, the only method so far approved by the U.S. Food and Drug Administration for clinical use is the Myriad Genetics method of HRD detection, i.e. the genomic scar method. In particular, the FDA approved PARPi, niraparib, for patients with advanced ovarian, fallopian tube, or primary peritoneal cancer treated with at least three chemotherapy regimens and whose cancer is associated with HRD-positive status determined by the Myriad’s myChoice HRD scoring method. With the increase of the OS PARPi sensitivity clinical data volume we will be able to establish the threshold of PARPi susceptibility in OS with our RF classifier.

In GSEA we expected that homologous recombination and other related signatures would be detected in downregulated genes of BRCAness samples, i.e. in gene sets with a negative enrichment score. Contrary to our expectations, these signatures were detected in upregulated genes. This could be a sign of genetic compensation in HR repair pathway, i.e. changes in RNA level in an attempt to functionally compensate for the loss of a function of a certain "BRCAness" gene [71]. Since we compared a set of unrelated OS samples, where practically any HR gene can be impaired, the resulting compensation in the form of overexpression can cover the whole spectrum of HR genes in its ineffective attempt to restore a functional HR pathway. Indeed, Pitroda *et al*. found that the degree of genomic instability in carcinoma cells determined by sensitivity to a specific chemotherapy agent, topotecan, was positively correlated with the overexpression of HR-related genes such as RAD51, BLM, BRCA1, RAD51AP1, RAD54B, BRCA2, RAD51C [72]. In HR-proficient settings RAD51 and other RAD51-associated genes are thought to promote HR. Nevertheless, RAD51 overexpression has been previously reported in BRCA1/2 mutant cancer cells exhibiting HRD phenotype [73]. Also, the overexpression of additional genes known to promote various mechanisms required for HR, including CtIP participating in ssDNA-end resection, PLK1 contributing to 53BP1 and RAD51 phosphorylation, and XRCC2, XRCC3, PALB2 participating in RAD51 filament assembly was reported in carcinoma cells with increased genomic instability. These findings indicated that BRCA-defective cells responded to HR-deficiency by increasing the expression of a number of HR-related genes [72]. In another study, the upregulation of Polθ and its positive correlation with expression of many DNA repair genes including TOPBP1, BLM, RAD54L, FANCI, BRCA1, FANCD2, ATAD5 was reported in HR-deficient epithelial ovarian cancer cells [74]. In our OS BRCAness-positive gene expression data the majority of the aforementioned genes were significantly overexpressed or showed a tendency towards upregulation contributing to HR and HR-related signatures obtained in GSEA.

Notably, our GSEA results showed that BRCAness-positive OS tumors exhibited immune signatures in downregulated genes with a reduction of immune-mediated cytotoxicity and inflammation. These findings were consistent with a pan-cancer analysis which found that aneuploidy, i.e. the presence of abnormal number of chromosomes, and a higher prevalence of large somatic copy number alterations (SCNA), a trait of HRD, were associated with lower immune cytotoxicity, possibly due to the genomic events disrupting cellular machinery associated with the recruitment of immune cells [75]. Among the gene sets upregulated in high versus low aneuploidy tumors were those implicated in DNA replication, cell cycle regulation, mitosis, and chromosome maintenance. At the same time, this pan-cancer data analysis showed that the level of aneuploidy and SCNA inversely correlated with cytotoxic immune signatures [75]. Davoli *et al*. postulated that aneuploidy may weaken some aspects of antigen presentation in MHC, important for tumor detection by the immune system. In another study, when BRCA1/2 mutant breast cancers were dichotomized by median HRD score, higher HRD scores were also associated with lower immunogenicity [76]. This study showed that the HRD-low subset of breast cancer tumors in a cohort was the most highly immunogenic with the strongest evidence for CD8-driven T-cell responses [76]. Our findings on these signatures suggest that estimation of BRCAness level might be useful for stratifying OS patients in clinical trials most likely responding not only to PARPi therapy but also to therapy based on immune checkpoint blockade [77].

In conclusion, we developed and validated an accurate DNA-methylation based classifier which can rapidly compute a degree of BRCAness in OS samples without the need for high computational power or high capacity data storage usually required for NGS genomic data processing. The finding of a HR expression signature is another proof of BRCAness in OS and the validity of the genomic scars’ method. Also, it can be used to develop a GEP panel for predicting PARPi susceptibility in OS. Our results demonstrate similar HR and HR-related gene expression signatures both in the training set categorized by the genomic scars’ method and in the independent test set categorized by the RF classifier. This serves as a proof of soundness of our methylation classifier implementation. A set of differentially expressed genes can be included in the advanced version of the classifier as an additional feature together with the mutation status of certain genes, e.g. CDK2/4/6, cyclin E1/D3, RB1, CDKN2A [78,79], moving towards multi-omics integration, which could not only assist clinicians in making a decision on PARPi administration, but also improve our understanding of the BRCAness molecular mechanism. We believe that both the DNA-methylation based classifier and gene expression signatures could provide a useful aid in decision making on administering PARPi to OS patients. This approach can be extended to other cancers having the characteristics of genome instability.

About 50% of OS patients have HR-deficient tumors. Yet, in a recent PRIMA clinical trial with PARPi olaparib as a first-line treatment of ovarian cancer patients, it was shown that with current HRD testing 30% of patients may be given unsuccessful PARPi treatment [80]. At the moment, health care costs are ever-increasing and new treatments are only justified if they are effective and well targeted. Therefore, the cost of PARPi treatment might influence survival chances. We envision that a DNA-methylation BRCAness classifier will contribute to the reduction in PARPi treatment cost by improving the identification of HRD tumors. This will make PARPi treatment much more personalized, cost-effective, will reduce economic burden on society and, above all, will decrease the unnecessary toxic and mutagenic burden on patients.

## Supporting information

S1 File Supporting Info: Document containing supplemental figures and tables. (PDF)

S2 File Tables: all OS samples used, DE genes, GSEA results. (ZIP)

S3 File R-scripts to reproduce random forest training, validation and to generate trained RF model. (ZIP)
