# Supplementary material for: DNA methylation-based classifier and gene expression signatures detect BRCAness in osteosarcoma
Source: PLoS Comput Biol. 2021 Nov 11;17(11):e1009562. doi: 10.1371/journal.pcbi.1009562 (PMC8584788; doi:10.1371/journal.pcbi.1009562)
Supplement: S1 File — (PDF) [file pcbi.1009562.s001.pdf]

Supplement figures

A

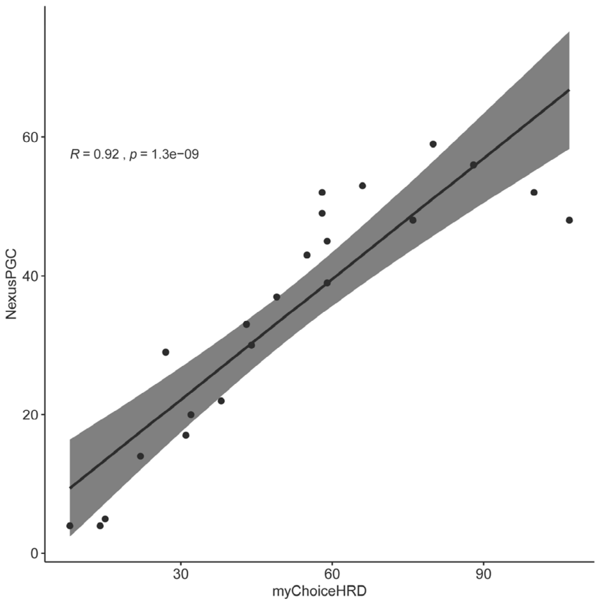

B

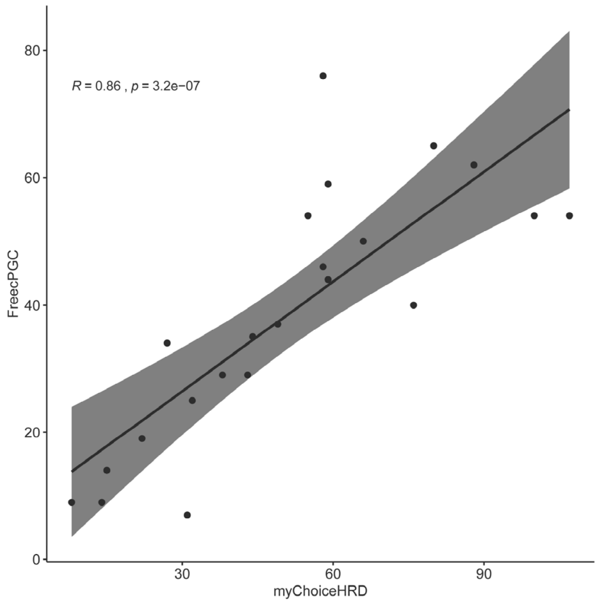

C

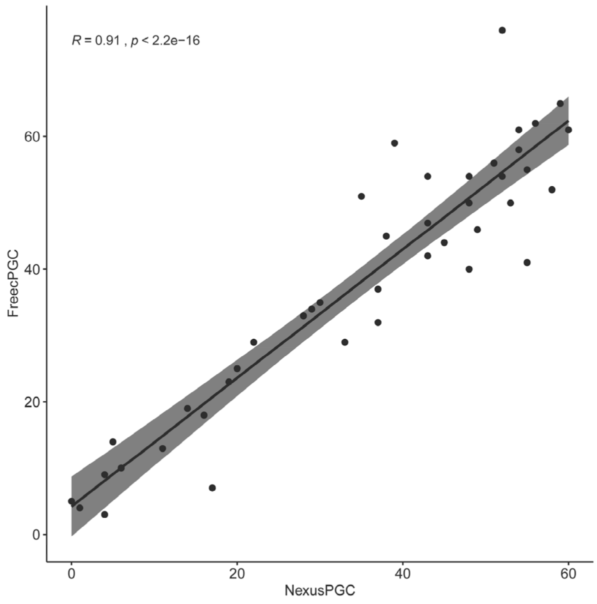

Fig A. Scatter plots (A) between MyChoice HRD score and Nexus PGC; (B) between MyChoice HRD score and FREEC-HRDtools PGC; (C) between Nexus PGC and Control-FREEC PGC. Spearman correlation is shown on the plot together with p-value. A grey area shows 95% confidence interval.

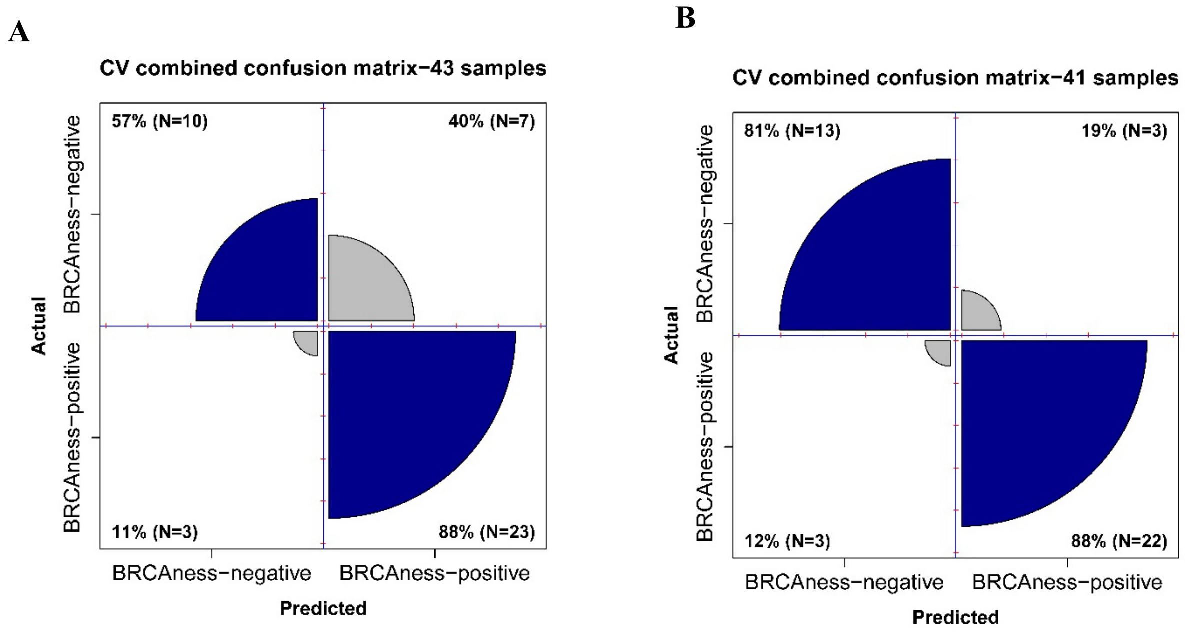

Fig B. Visual confusion matrices derived from models built on (A) 43 and (B) 41 sample sets. The sizes of blue slices correspond to the true positive and true negative rates while the sizes of the grey slices correspond to the false positive and false negative rates.

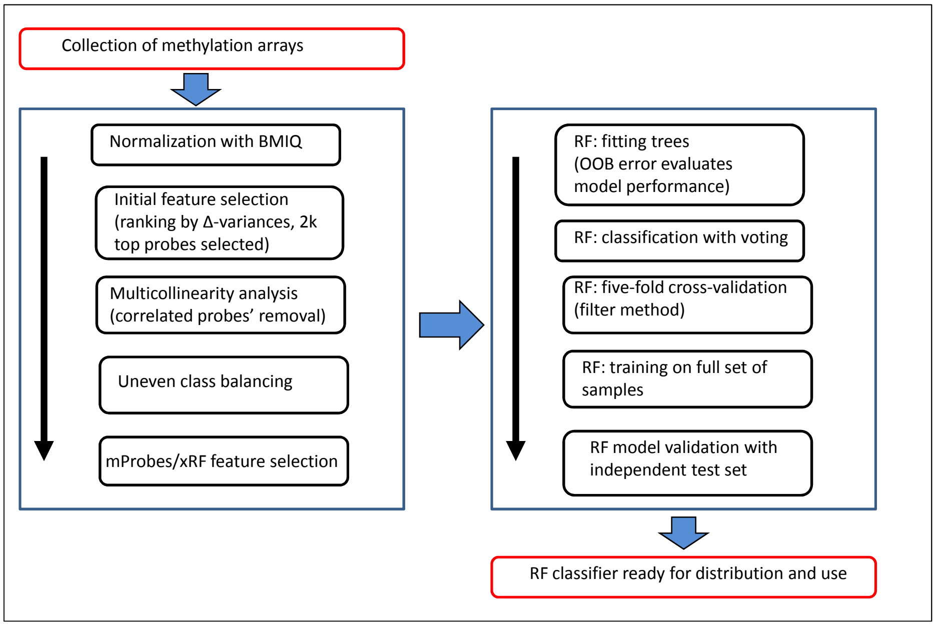

Fig C. Pipeline for data processing, RF classifier training and validation.

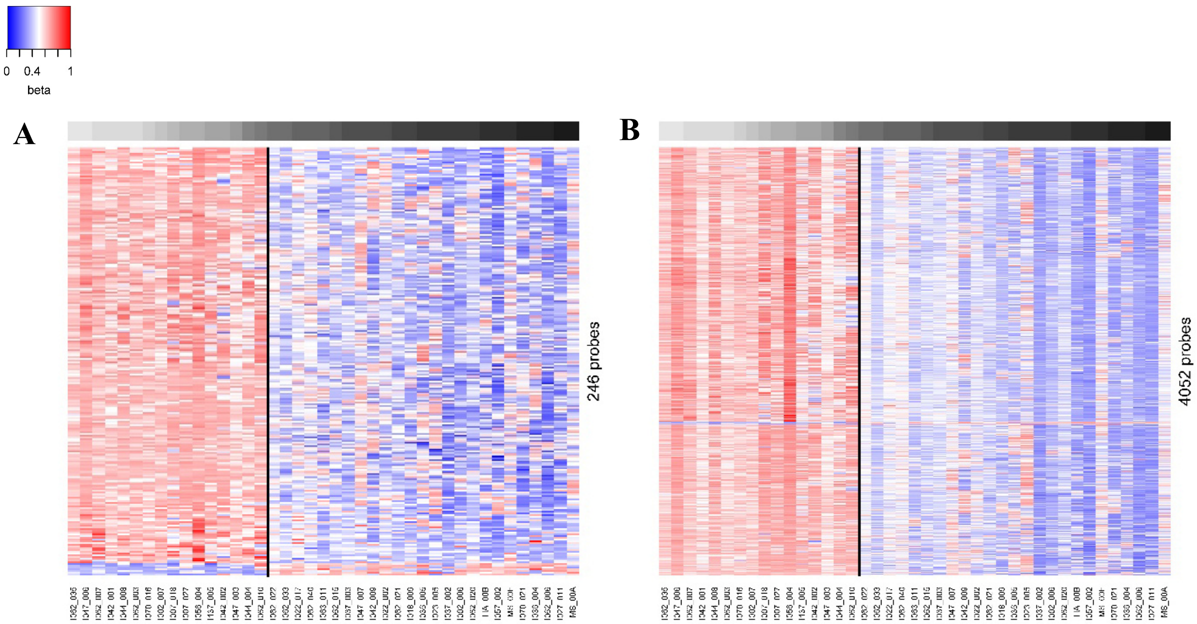

Fig D. Heatmaps of samples arranged by Nexus PGC. (A) 246 probes identified as important non-correlated predictors combined after 5-fold cross validation and (B) 4052 probes correlated to and including 246 probes show decrease of intensity as Nexus PGC increases (indicated by the bar at the top). The columns are probe intensities in each sample ordered by increase in Nexus PGC (low to high, from left to right) and separated by black vertical line into BRCAness negative and BRCAness-positive samples on the left and right, correspondingly. Please note, for the final RF model, used for validation with independent test set (n=20), all 41 samples were used as one training set. This set yielded 54 uncorrelated probes out of 2000 probes.

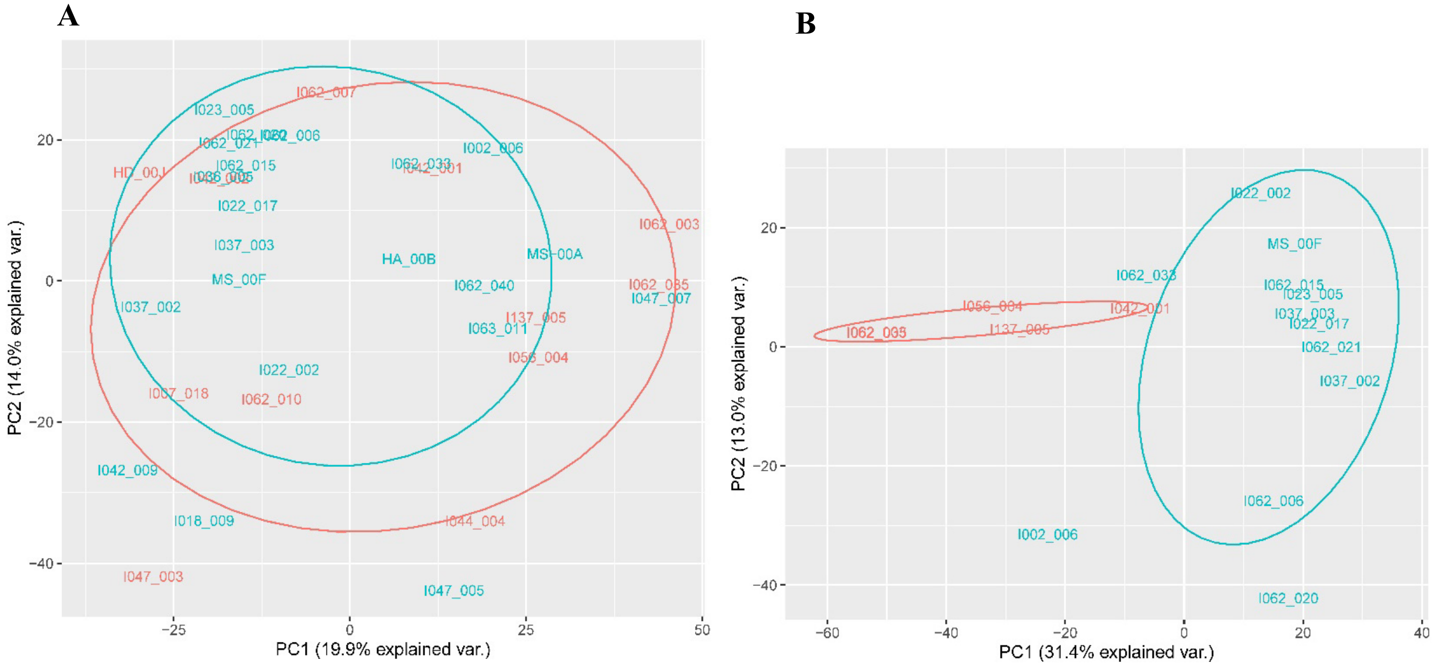

Fig E. PCA 1 and 2 of (A) all 33 and (B) 17 RNA-Seq samples selected from two most homogenous clusters. BRCAness-positive samples denoted by cyan color and BRCAness-negative by red color. Ellipses show a confidence region for normal data.

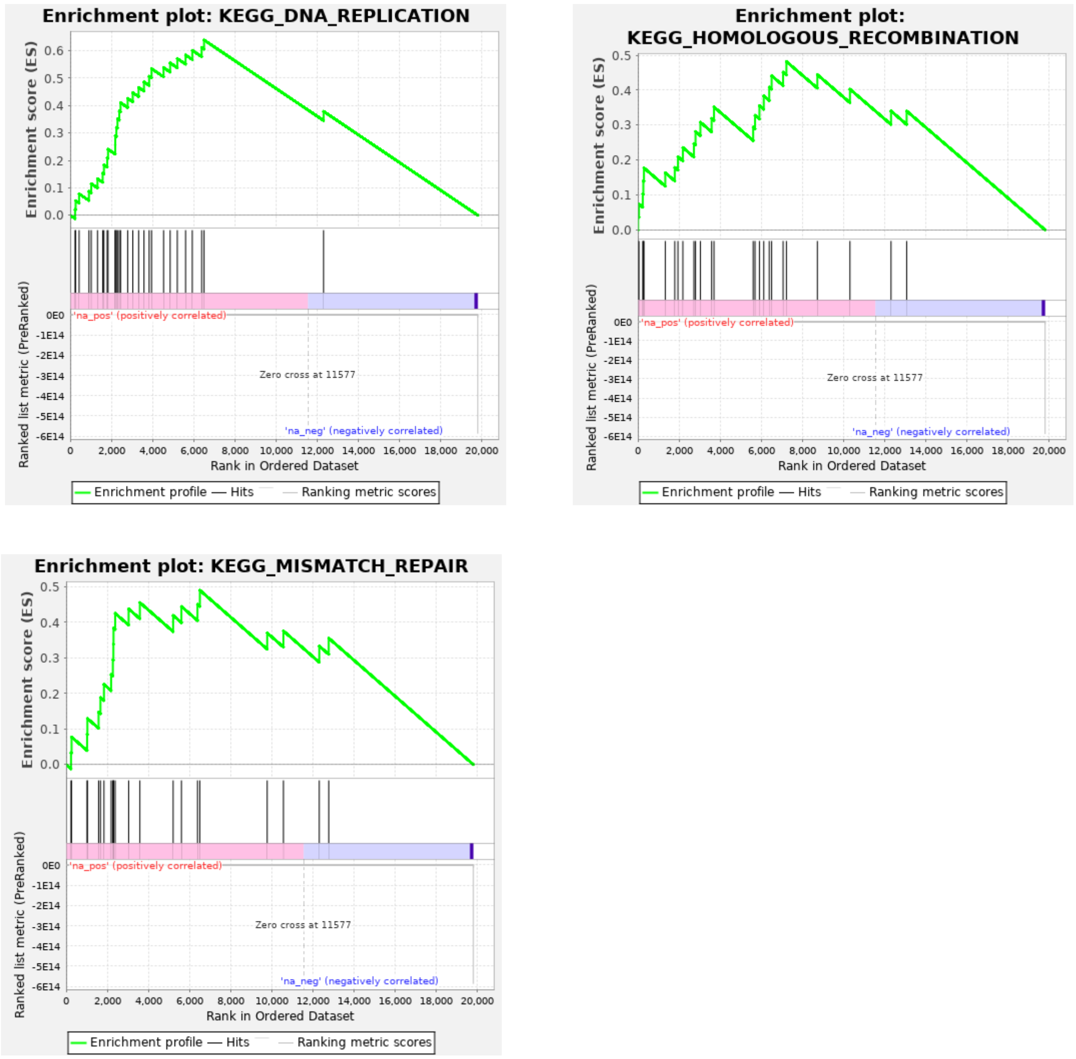

Fig F. GSEA enrichment plots for gene sets with a positive enrichment score.

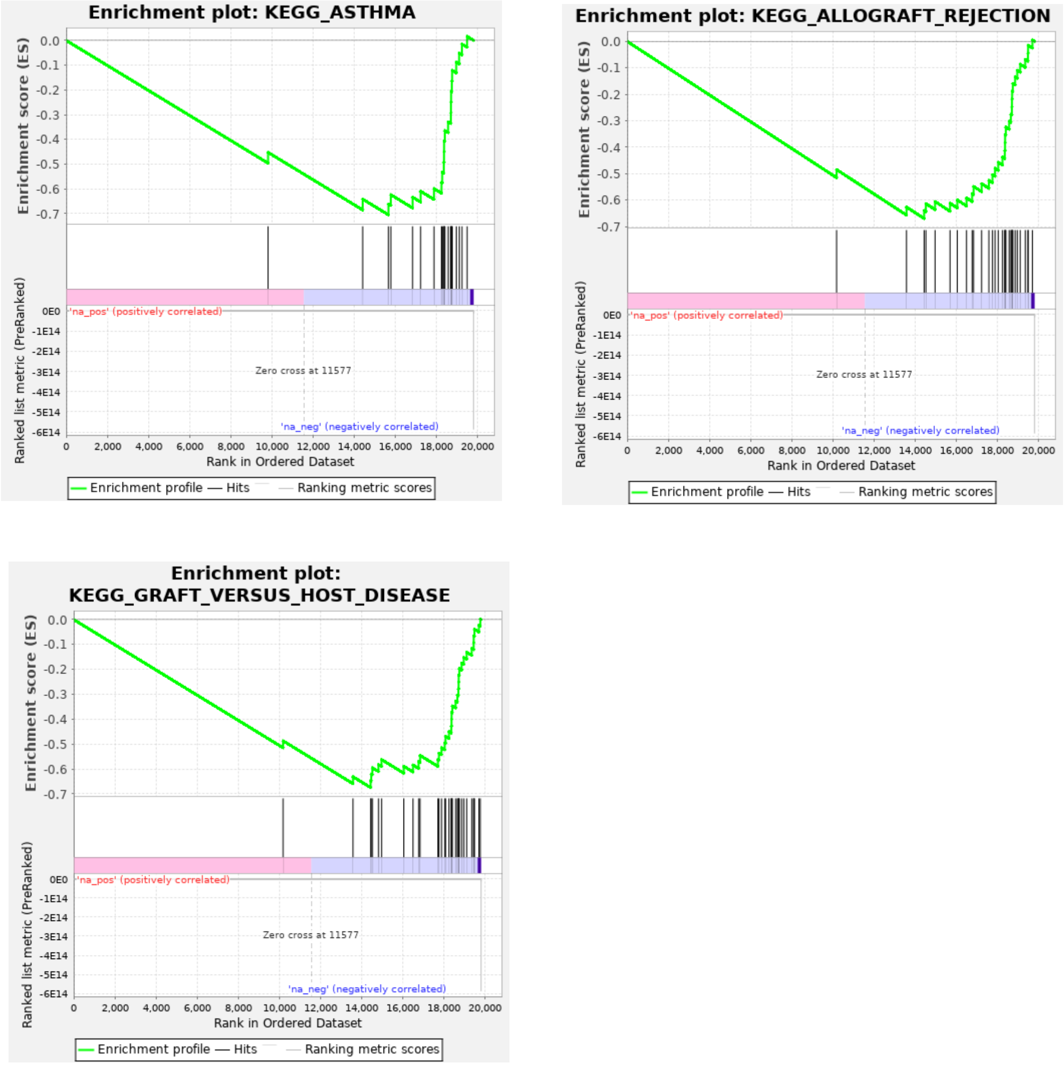

Fig G. GSEA enrichment plots for gene sets with a negative enrichment score.

Supplement tables

Table A. Count of positive Δ-variances after removing samples consecutively in 43 sample set. Third column shows whether this sample is BRCAness positive according to Nexus PGC

sample ID	count delta Var > 0	BRCAness positive group
MS_00A	1310	yes
I027_011	913	yes
I062_006	920	yes
I070_021	969	yes
I036_004	1009	yes
MS_00F	1365	yes
HA_00B	948	yes
I057_002	932	yes
I047_005	1932	yes
I037_002	910	yes
I002_006	945	yes
I062_020	1036	yes
I036_005	1179	yes
I023_005	1621	yes
I018_009	997	yes
I062_021	1011	yes
I047_007	1378	yes
I042_009	1168	yes
I022_002	1127	yes
I037_003	1056	yes
I062_015	1054	yes
I063_011	1047	yes
I022_017	1228	yes
I062_040	1339	yes
I062_033	1008	yes
I062_022	1233	yes
I062_010	1324	no
HD_00J	2195	no
I044_004	1331	no
I047_003	1284	no
I042_002	1429	no
I137_005	1315	no
I056_004	900	no
I007_027	898	no
I007_018	1025	no
I002_007	952	no
I070_016	914	no
I062_003	1023	no
I062_007	857	no
I042_001	1108	no
I044_008	888	no
I047_006	812	no
I062_035	905	no

Table B. Selected gene sets with a positive enrichment score. Genes with Yes value contribute most to the enrichment results

**KEGG DNA Replication**	
GENE SYMBOL	CORE ENRICHMENT
RPA3	Yes
POLD2	Yes
PRIM1	Yes
POLE2	Yes
RFC5	Yes
POLA1	Yes
RFC2	Yes
FEN1	Yes
MCM3	Yes
RFC3	Yes
POLD1	Yes
MCM2	Yes
POLE	Yes
LIG1	Yes
RFC4	Yes
POLA2	Yes
MCM6	Yes
POLD3	Yes
PRIM2	Yes
RPA2	Yes
POLE4	Yes
MCM4	Yes
MCM7	Yes
POLE3	Yes
RFC1	Yes
RPA4	Yes
MCM5	Yes
SSBP1	Yes
RPA1	Yes
POLD4	No

**KEGG Homologous Recombination**	
GENE SYMBOL	CORE ENRICHMENT
EME1	Yes
TOP3A	Yes
RPA3	Yes
POLD2	Yes
BLM	Yes
RAD51	Yes
RAD54L	Yes
RAD51D	Yes
POLD1	Yes
RAD54B	Yes
RAD51B	Yes
POLD3	Yes
RPA2	Yes
RAD52	Yes
RPA4	Yes
XRCC3	Yes
BRCA2	Yes
XRCC2	Yes
SSBP1	Yes
RPA1	Yes
MUS81	Yes
RAD51C	Yes
NBN	No
TOP3B	No
POLD4	No
RAD50	No

**KEGG Mismatch Repair**	
GENE SYMBOL	CORE ENRICHMENT
RPA3	Yes
POLD2	Yes
RFC5	Yes
EXO1	Yes
RFC2	Yes
PMS2	Yes
RFC3	Yes
POLD1	Yes
MSH6	Yes
LIG1	Yes
MSH2	Yes
RFC4	Yes
POLD3	Yes
RPA2	Yes
RFC1	Yes
RPA4	Yes
SSBP1	Yes
RPA1	Yes
MLH1	No
MLH3	No
POLD4	No
MSH3	No

Table C. Selected gene sets with a negative enrichment score. Genes with Yes value contribute most to the enrichment results

**KEGG Asthma**	
GENE SYMBOL	CORE ENRICHMENT
EPX	No
PRG2	No
FCER1G	Yes
IL13	Yes
TNF	Yes
CD40	Yes
HLA-DQA2	Yes
HLA-DPB1	Yes
FCER1A	Yes
HLA-DPA1	Yes
HLA-DMA	Yes
HLA-DRB1	Yes
HLA-DOA	Yes
HLA-DMB	Yes
HLA-DRA	Yes
IL10	Yes
HLA-DRB5	Yes
HLA-DOB	Yes
CCL11	Yes
HLA-DQA1	Yes
HLA-DQB1	Yes
MS4A2	Yes
CD40LG	Yes

**KEGG Allograft Rejection**	
GENE SYMBOL	CORE ENRICHMENT
HLA-C	No
HLA-A	No
CD86	Yes
IL2	Yes
HLA-B	Yes
IL12A	Yes
HLA-F	Yes
CD28	Yes
HLA-G	Yes
TNF	Yes
CD40	Yes
IL12B	Yes
GZMB	Yes
HLA-DQA2	Yes
FAS	Yes
HLA-DPB1	Yes
HLA-DPA1	Yes
HLA-DMA	Yes
HLA-DRB1	Yes
HLA-DOA	Yes
HLA-DMB	Yes
HLA-E	Yes
HLA-DRA	Yes
IL10	Yes
HLA-DRB5	Yes
HLA-DOB	Yes
CD80	Yes
HLA-DQA1	Yes
HLA-DQB1	Yes
FASLG	Yes
PRF1	Yes
CD40LG	Yes
IFNG	Yes

**KEGG Graft Versus Host Disease**	
GENE SYMBOL	CORE ENRICHMENT
HLA-C	No
HLA-A	No
CD86	Yes
IL1B	Yes
IL2	Yes
IL6	Yes
HLA-B	Yes
HLA-F	Yes
CD28	Yes
HLA-G	Yes
TNF	Yes
KIR2DL3	Yes
GZMB	Yes
HLA-DQA2	Yes
FAS	Yes
KIR3DL1	Yes
HLA-DPB1	Yes
HLA-DPA1	Yes
HLA-DMA	Yes
HLA-DRB1	Yes
HLA-DOA	Yes
HLA-DMB	Yes
HLA-E	Yes
HLA-DRA	Yes
HLA-DRB5	Yes
KIR3DL2	Yes
HLA-DOB	Yes
CD80	Yes
HLA-DQA1	Yes
HLA-DQB1	Yes
FASLG	Yes
KLRC1	Yes
IL1A	Yes
PRF1	Yes
IFNG	Yes
KLRD1	Yes

Table D. Test set Reactome enrichment results (positive enrichment score only)

GSEA	NAME	SIZE	ES	NES	NOM p-val	FDR q-val	FWER p-val
Positive enrichment score	REACTOME_CELL_CYCLE_MITOTIC	466	0.210	5.211	0	0	0
	REACTOME_MITOTIC_PROMETAPHASE	191	0.274	4.441	0	0	0
	REACTOME_CELL_CYCLE_CHECKPOINTS	234	0.237	4.210	0	0	0
	REACTOME_M_PHASE	329	0.196	4.167	0	0	0
	REACTOME_RESOLUTION_OF_SISTER_CHROMATID_COHESION	116	0.330	4.164	0	0	0
	REACTOME_MITOTIC_SPINDLE_CHECKPOINT	97	0.351	4.078	0	0	0
	REACTOME_ACTIVATION_OF_ATR_IN_RESPONSE_TO_REPLICATION_STRESS	37	0.481	3.478	0	0	0
	REACTOME_RHO_GTPASES_ACTIVATE_FORMINS	130	0.260	3.458	0	0	0
	REACTOME_MITOTIC_METAPHASE_AND_ANAPHASE	213	0.203	3.440	0	0	0
	REACTOME_DNA_STRAND_ELONGATION	30	0.514	3.340	0	0	0
	REACTOME_RHO_GTPASE_EFFECTORS	247	0.177	3.269	0	0	0
	REACTOME_CILIUM_ASSEMBLY	194	0.200	3.230	0	0	0
	REACTOME_HOMOLOGOUS_DNA_PAIRING_AND_STRAND_EXCHANGE	40	0.422	3.176	0	0	0
	REACTOME_SEPARATION_OF_SISTER_CHROMATIDS	170	0.207	3.151	0	0	0
	REACTOME_ACTIVATION_OF_THE_PRE_REPLICATIVE_COMPLEX	33	0.458	3.105	0	2E-05	3E-04
	REACTOME_HDR_THROUGH_HOMOLOGOUS_RECOMBINATION_HRR_	62	0.327	3.009	0	5E-05	7E-04
	REACTOME_ORGANELLE_BIOGENESIS_AND_MAINTENANCE	264	0.155	2.950	0	1E-04	0.002
	REACTOME_HDR_THROUGH_SINGLE_STRAND_ANNEALING_SSA_	35	0.419	2.942	0	1E-04	0.002
	REACTOME_E2F_MEDIATED_REGULATION_OF_DNA_REPLICATION	22	0.501	2.829	0	3E-04	0.005
	REACTOME_S_PHASE	143	0.205	2.819	0	3E-04	0.005
	REACTOME_DEPOSITION_OF_NEW_CENPA_CONTAINING_NUCLEOSOMES	25	0.460	2.777	0	5E-04	0.008
	REACTOME_RESOLUTION_OF_D_LOOP_STRUCTURES	30	0.426	2.769	0	5E-04	0.009
	REACTOME_RESOLUTION_OF_D_LOOP_STRUCTURES_THROUGH_SYNTHESIS	24	0.457	2.672	0	0.001	0.020
	REACTOME_DNA_DOUBLE_STRAND_BREAK_REPAIR	120	0.207	2.636	0	0.001	0.027
	REACTOME_TELOMERE_C_STRAND_LAGGING_STRAND_SYNTHESIS	30	0.404	2.626	7E-04	0.001	0.029
	REACTOME_INSULIN_PROCESSING	23	0.453	2.623	0	0.001	0.030
	REACTOME_COPI_DEPENDENT_GOLGI_TO_ER_RETROGRADE_TRAFFIC	90	0.236	2.611	0	0.001	0.033
	REACTOME_HOMOLOGY_DIRECTED_REPAIR	94	0.233	2.611	0	0.001	0.033
	REACTOME_SUMOYLATION	164	0.174	2.588	0	0.002	0.039
	REACTOME_G2_M_CHECKPOINTS	127	0.196	2.579	7E-04	0.002	0.042
	REACTOME_INTRA_GOLGI_AND_RETROGRADE_GOLGI_TO_ER_TRAFFIC	188	0.162	2.561	0	0.002	0.049

Table E. Test set KEGG enrichment results

GSEA	NAME	SIZE	ES	NES	NOM p-val	FDR q-val	FWER p-val
Positive enrichment score	KEGG_CELL_CYCLE	110	0.275	3.363	0.000	0.000	0.000
	KEGG_DNA_REPLICATION	30	0.402	2.626	0.000	0.002	0.004
	KEGG_OOCYTE_MEIOSIS	94	0.226	2.553	0.000	0.002	0.007
	KEGG_PROGESTERONE_MEDIATED_OOCYTE	67	0.240	2.344	0.001	0.009	0.041
	KEGG_MISMATCH_REPAIR	22	0.386	2.149	0.001	0.030	0.157
	KEGG_HOMOLOGOUS_RECOMBINATION	26	0.337	2.064	0.004	0.042	0.261
	KEGG_GLYCOSYLPHOSPHATIDYLINOSITOL_GPI	24	0.348	2.026	0.005	0.046	0.316
Negative enrichment score	KEGG_RIBOSOME	86	-0.374	-4.092	0.000	0.000	0.000
	KEGG_CYTOKINE_CYTOKINE_RECEPTOR_INTERACTION	202	-0.248	-4.060	0.000	0.000	0.000
	KEGG_GRAFT_VERSUS_HOST_DISEASE	29	-0.480	-3.073	0.000	0.000	0.000
	KEGG_ARACHIDONIC_ACID_METABOLISM	46	-0.386	-3.067	0.000	0.000	0.000
	KEGG_INTESTINAL_IMMUNE_NETWORK_FOR_IGA_	40	-0.402	-3.006	0.000	0.000	0.000
	KEGG_ALLOGRAFT_REJECTION	30	-0.424	-2.766	0.000	0.000	0.001
	KEGG_AUTOIMMUNE_THYROID_DISEASE	30	-0.400	-2.634	0.000	0.001	0.006
	KEGG_LEISHMANIA_INFECTION	64	-0.277	-2.599	0.000	0.001	0.007
	KEGG_TYPE_I_DIABETES_MELLITUS	34	-0.371	-2.539	0.000	0.002	0.011
	KEGG_ASTHMA	21	-0.443	-2.425	0.000	0.003	0.027
	KEGG_NOD_LIKE_RECEPTOR_SIGNALING_PATHWAY	55	-0.267	-2.327	0.000	0.005	0.050
	KEGG_CELL_ADHESION_MOLECULES_CAMS	109	-0.191	-2.329	0.001	0.005	0.049
	KEGG_APOPTOSIS	81	-0.219	-2.340	0.001	0.005	0.045
	KEGG_HEMATOPOIETIC_CELL_LINEAGE	74	-0.220	-2.227	0.001	0.009	0.102
	KEGG_PARKINSONS_DISEASE	96	-0.177	-2.039	0.007	0.027	0.302
	KEGG_PRIMARY_IMMUNODEFICIENCY	33	-0.293	-2.012	0.001	0.030	0.343
	KEGG_CHEMOKINE_SIGNALING_PATHWAY	167	-0.134	-1.988	0.006	0.032	0.379
