# Supplementary material for: DNA methylation-based classifier and gene expression signatures detect BRCAness in osteosarcoma
Source: PLoS Comput Biol. 2021 Nov 11;17(11):e1009562. doi: 10.1371/journal.pcbi.1009562 (PMC8584788; doi:10.1371/journal.pcbi.1009562)
Supplement: S2 File — (ZIP) [file pcbi.1009562.s002.zip › S2_File/my_analysis_Kegg.GseaPreranked.1581692187239/gsea_report_for_na_neg_1581692187239.html]

Report for na\_neg 1581692187239 [GSEA]

| GS  follow link to MSigDB | GS DETAILS | SIZE | ES | NES | NOM p-val | FDR q-val | FWER p-val | RANK AT MAX | LEADING EDGE || 1 | KEGG\_ASTHMA | Details ... | 23 | -0.70 | -0.70 | 0.000 | 0.000 | 0.000 | 4145 | tags=91%, list=21%, signal=115% |
| 2 | KEGG\_GRAFT\_VERSUS\_HOST\_DISEASE | Details ... | 36 | -0.67 | -0.67 | 0.000 | 0.000 | 0.000 | 5378 | tags=94%, list=27%, signal=129% |
| 3 | KEGG\_ALLOGRAFT\_REJECTION | Details ... | 33 | -0.67 | -0.67 | 0.000 | 0.000 | 0.000 | 5378 | tags=94%, list=27%, signal=129% |
| 4 | KEGG\_TYPE\_I\_DIABETES\_MELLITUS | Details ... | 39 | -0.60 | -0.60 | 0.000 | 0.000 | 0.000 | 5378 | tags=87%, list=27%, signal=119% |
| 5 | KEGG\_AUTOIMMUNE\_THYROID\_DISEASE | Details ... | 33 | -0.58 | -0.58 | 0.000 | 0.000 | 0.000 | 5378 | tags=85%, list=27%, signal=116% |
| 6 | KEGG\_HEMATOPOIETIC\_CELL\_LINEAGE | Details ... | 80 | -0.55 | -0.55 | 0.000 | 0.000 | 0.000 | 5521 | tags=83%, list=28%, signal=114% |
| 7 | KEGG\_RENIN\_ANGIOTENSIN\_SYSTEM | Details ... | 16 | -0.55 | -0.55 | 0.000 | 0.000 | 0.000 | 6511 | tags=88%, list=33%, signal=130% |
| 8 | KEGG\_INTESTINAL\_IMMUNE\_NETWORK\_FOR\_IGA\_PRODUCTION | Details ... | 43 | -0.49 | -0.49 | 0.000 | 0.001 | 0.003 | 5488 | tags=77%, list=28%, signal=106% |
| 9 | KEGG\_COMPLEMENT\_AND\_COAGULATION\_CASCADES | Details ... | 61 | -0.49 | -0.49 | 0.000 | 0.001 | 0.004 | 4356 | tags=70%, list=22%, signal=90% |
| 10 | KEGG\_CELL\_ADHESION\_MOLECULES\_CAMS | Details ... | 122 | -0.47 | -0.47 | 0.000 | 0.001 | 0.005 | 5563 | tags=75%, list=28%, signal=103% |
| 11 | KEGG\_PRIMARY\_IMMUNODEFICIENCY | Details ... | 34 | -0.45 | -0.45 | 0.000 | 0.001 | 0.009 | 2689 | tags=59%, list=14%, signal=68% |
| 12 | KEGG\_LINOLEIC\_ACID\_METABOLISM | Details ... | 22 | -0.43 | -0.43 | 0.001 | 0.002 | 0.021 | 6838 | tags=77%, list=34%, signal=118% |
| 13 | KEGG\_LEISHMANIA\_INFECTION | Details ... | 69 | -0.43 | -0.43 | 0.000 | 0.002 | 0.023 | 7373 | tags=80%, list=37%, signal=126% |
| 14 | KEGG\_CYTOKINE\_CYTOKINE\_RECEPTOR\_INTERACTION | Details ... | 226 | -0.39 | -0.39 | 0.000 | 0.006 | 0.062 | 5716 | tags=68%, list=29%, signal=94% |
| 15 | KEGG\_ANTIGEN\_PROCESSING\_AND\_PRESENTATION | Details ... | 64 | -0.39 | -0.39 | 0.000 | 0.007 | 0.074 | 5039 | tags=64%, list=25%, signal=86% |
| 16 | KEGG\_GLYCOSPHINGOLIPID\_BIOSYNTHESIS\_LACTO\_AND\_NEOLACTO\_SERIES | Details ... | 26 | -0.37 | -0.37 | 0.000 | 0.012 | 0.137 | 4942 | tags=62%, list=25%, signal=82% |
| 17 | KEGG\_LYSOSOME | Details ... | 113 | -0.34 | -0.34 | 0.000 | 0.020 | 0.231 | 8642 | tags=78%, list=44%, signal=137% |
| 18 | KEGG\_DRUG\_METABOLISM\_CYTOCHROME\_P450 | Details ... | 54 | -0.34 | -0.34 | 0.000 | 0.020 | 0.236 | 4944 | tags=59%, list=25%, signal=79% |
| 19 | KEGG\_EPITHELIAL\_CELL\_SIGNALING\_IN\_HELICOBACTER\_PYLORI\_INFECTION | Details ... | 67 | -0.34 | -0.34 | 0.000 | 0.019 | 0.237 | 7410 | tags=72%, list=37%, signal=114% |
| 20 | KEGG\_NATURAL\_KILLER\_CELL\_MEDIATED\_CYTOTOXICITY | Details ... | 114 | -0.33 | -0.33 | 0.000 | 0.029 | 0.362 | 7307 | tags=69%, list=37%, signal=109% |
| 21 | KEGG\_METABOLISM\_OF\_XENOBIOTICS\_BY\_CYTOCHROME\_P450 |  | 53 | -0.32 | -0.32 | 0.000 | 0.034 | 0.433 | 4944 | tags=57%, list=25%, signal=75% |
| 22 | KEGG\_NOD\_LIKE\_RECEPTOR\_SIGNALING\_PATHWAY |  | 59 | -0.32 | -0.32 | 0.001 | 0.033 | 0.435 | 7173 | tags=68%, list=36%, signal=106% |
| 23 | KEGG\_ETHER\_LIPID\_METABOLISM |  | 25 | -0.32 | -0.32 | 0.010 | 0.032 | 0.441 | 11185 | tags=88%, list=56%, signal=202% |
| 24 | KEGG\_ARACHIDONIC\_ACID\_METABOLISM |  | 48 | -0.32 | -0.32 | 0.001 | 0.031 | 0.443 | 4901 | tags=56%, list=25%, signal=75% |
| 25 | KEGG\_GLYCOSAMINOGLYCAN\_DEGRADATION |  | 19 | -0.31 | -0.31 | 0.034 | 0.032 | 0.465 | 8405 | tags=74%, list=42%, signal=128% |
| 26 | KEGG\_LEUKOCYTE\_TRANSENDOTHELIAL\_MIGRATION |  | 103 | -0.31 | -0.31 | 0.000 | 0.032 | 0.477 | 7523 | tags=69%, list=38%, signal=111% |
| 27 | KEGG\_STEROID\_BIOSYNTHESIS |  | 17 | -0.31 | -0.31 | 0.058 | 0.035 | 0.519 | 9082 | tags=76%, list=46%, signal=141% |
| 28 | KEGG\_PHENYLALANINE\_METABOLISM |  | 15 | -0.31 | -0.31 | 0.100 | 0.034 | 0.519 | 12427 | tags=93%, list=63%, signal=250% |
| 29 | KEGG\_JAK\_STAT\_SIGNALING\_PATHWAY |  | 124 | -0.30 | -0.30 | 0.000 | 0.039 | 0.580 | 6559 | tags=63%, list=33%, signal=93% |
| 30 | KEGG\_ALDOSTERONE\_REGULATED\_SODIUM\_REABSORPTION |  | 41 | -0.30 | -0.30 | 0.000 | 0.039 | 0.597 | 3778 | tags=49%, list=19%, signal=60% |
| 31 | KEGG\_TOLL\_LIKE\_RECEPTOR\_SIGNALING\_PATHWAY |  | 87 | -0.30 | -0.30 | 0.000 | 0.039 | 0.609 | 7140 | tags=66%, list=36%, signal=102% |
| 32 | KEGG\_SPHINGOLIPID\_METABOLISM |  | 35 | -0.30 | -0.30 | 0.001 | 0.039 | 0.616 | 10574 | tags=83%, list=53%, signal=177% |
| 33 | KEGG\_VIRAL\_MYOCARDITIS |  | 66 | -0.30 | -0.30 | 0.000 | 0.039 | 0.621 | 7385 | tags=67%, list=37%, signal=106% |
| 34 | KEGG\_CHEMOKINE\_SIGNALING\_PATHWAY |  | 174 | -0.29 | -0.29 | 0.000 | 0.047 | 0.712 | 7150 | tags=64%, list=36%, signal=100% |
| 35 | KEGG\_TYROSINE\_METABOLISM |  | 36 | -0.28 | -0.28 | 0.010 | 0.053 | 0.761 | 5476 | tags=56%, list=28%, signal=77% |
| 36 | KEGG\_B\_CELL\_RECEPTOR\_SIGNALING\_PATHWAY |  | 75 | -0.28 | -0.28 | 0.000 | 0.052 | 0.768 | 8236 | tags=69%, list=42%, signal=118% |
| 37 | KEGG\_T\_CELL\_RECEPTOR\_SIGNALING\_PATHWAY |  | 105 | -0.28 | -0.28 | 0.000 | 0.055 | 0.794 | 10042 | tags=78%, list=51%, signal=157% |
| 38 | KEGG\_OTHER\_GLYCAN\_DEGRADATION |  | 16 | -0.27 | -0.27 | 0.166 | 0.063 | 0.843 | 10771 | tags=81%, list=54%, signal=178% |
| 39 | KEGG\_TRYPTOPHAN\_METABOLISM |  | 37 | -0.27 | -0.27 | 0.008 | 0.063 | 0.854 | 3267 | tags=43%, list=16%, signal=52% |
| 40 | KEGG\_RETINOL\_METABOLISM |  | 49 | -0.27 | -0.27 | 0.002 | 0.065 | 0.869 | 4864 | tags=51%, list=25%, signal=67% |
| 41 | KEGG\_FC\_EPSILON\_RI\_SIGNALING\_PATHWAY |  | 71 | -0.27 | -0.27 | 0.000 | 0.063 | 0.869 | 4250 | tags=48%, list=21%, signal=61% |
| 42 | KEGG\_PANTOTHENATE\_AND\_COA\_BIOSYNTHESIS |  | 16 | -0.24 | -0.24 | 0.274 | 0.117 | 0.982 | 6452 | tags=56%, list=33%, signal=83% |
| 43 | KEGG\_STEROID\_HORMONE\_BIOSYNTHESIS |  | 40 | -0.24 | -0.24 | 0.021 | 0.116 | 0.984 | 3748 | tags=43%, list=19%, signal=52% |
| 44 | KEGG\_ARRHYTHMOGENIC\_RIGHT\_VENTRICULAR\_CARDIOMYOPATHY\_ARVC |  | 59 | -0.24 | -0.24 | 0.005 | 0.116 | 0.985 | 9123 | tags=69%, list=46%, signal=128% |
| 45 | KEGG\_APOPTOSIS |  | 86 | -0.23 | -0.23 | 0.000 | 0.130 | 0.991 | 9067 | tags=69%, list=46%, signal=126% |
| 46 | KEGG\_HYPERTROPHIC\_CARDIOMYOPATHY\_HCM |  | 70 | -0.23 | -0.23 | 0.003 | 0.137 | 0.993 | 9123 | tags=69%, list=46%, signal=127% |
| 47 | KEGG\_ECM\_RECEPTOR\_INTERACTION |  | 79 | -0.23 | -0.23 | 0.001 | 0.135 | 0.994 | 6832 | tags=57%, list=34%, signal=87% |
| 48 | KEGG\_MATURITY\_ONSET\_DIABETES\_OF\_THE\_YOUNG |  | 18 | -0.22 | -0.22 | 0.332 | 0.163 | 0.997 | 3420 | tags=39%, list=17%, signal=47% |
| 49 | KEGG\_CYTOSOLIC\_DNA\_SENSING\_PATHWAY |  | 41 | -0.21 | -0.21 | 0.033 | 0.173 | 0.998 | 6913 | tags=56%, list=35%, signal=86% |
| 50 | KEGG\_GLYCEROPHOSPHOLIPID\_METABOLISM |  | 66 | -0.21 | -0.21 | 0.003 | 0.171 | 0.999 | 6918 | tags=56%, list=35%, signal=86% |
| 51 | KEGG\_FC\_GAMMA\_R\_MEDIATED\_PHAGOCYTOSIS |  | 91 | -0.21 | -0.21 | 0.000 | 0.170 | 0.999 | 7150 | tags=57%, list=36%, signal=89% |
| 52 | KEGG\_RIG\_I\_LIKE\_RECEPTOR\_SIGNALING\_PATHWAY |  | 57 | -0.21 | -0.21 | 0.010 | 0.169 | 0.999 | 14258 | tags=93%, list=72%, signal=330% |
| 53 | KEGG\_TIGHT\_JUNCTION |  | 117 | -0.21 | -0.21 | 0.000 | 0.177 | 1.000 | 7418 | tags=58%, list=37%, signal=92% |
| 54 | KEGG\_ABC\_TRANSPORTERS |  | 44 | -0.21 | -0.21 | 0.035 | 0.185 | 1.000 | 9909 | tags=70%, list=50%, signal=141% |
| 55 | KEGG\_DORSO\_VENTRAL\_AXIS\_FORMATION |  | 23 | -0.20 | -0.20 | 0.259 | 0.184 | 1.000 | 11462 | tags=78%, list=58%, signal=185% |
| 56 | KEGG\_ADHERENS\_JUNCTION |  | 63 | -0.20 | -0.20 | 0.009 | 0.190 | 1.000 | 9848 | tags=70%, list=50%, signal=138% |
| 57 | KEGG\_VIBRIO\_CHOLERAE\_INFECTION |  | 52 | -0.20 | -0.20 | 0.023 | 0.193 | 1.000 | 7089 | tags=56%, list=36%, signal=87% |
| 58 | KEGG\_PRION\_DISEASES |  | 35 | -0.20 | -0.20 | 0.089 | 0.191 | 1.000 | 9067 | tags=66%, list=46%, signal=121% |
| 59 | KEGG\_O\_GLYCAN\_BIOSYNTHESIS |  | 27 | -0.20 | -0.20 | 0.212 | 0.194 | 1.000 | 6344 | tags=52%, list=32%, signal=76% |
| 60 | KEGG\_BIOSYNTHESIS\_OF\_UNSATURATED\_FATTY\_ACIDS |  | 22 | -0.19 | -0.19 | 0.355 | 0.221 | 1.000 | 16030 | tags=100%, list=81%, signal=522% |
| 61 | KEGG\_PHOSPHATIDYLINOSITOL\_SIGNALING\_SYSTEM |  | 72 | -0.19 | -0.19 | 0.007 | 0.223 | 1.000 | 7249 | tags=56%, list=37%, signal=87% |
| 62 | KEGG\_TYPE\_II\_DIABETES\_MELLITUS |  | 38 | -0.19 | -0.19 | 0.124 | 0.227 | 1.000 | 3046 | tags=34%, list=15%, signal=40% |
| 63 | KEGG\_DILATED\_CARDIOMYOPATHY |  | 75 | -0.19 | -0.19 | 0.013 | 0.230 | 1.000 | 9251 | tags=65%, list=47%, signal=122% |
| 64 | KEGG\_PROXIMAL\_TUBULE\_BICARBONATE\_RECLAMATION |  | 23 | -0.19 | -0.19 | 0.350 | 0.232 | 1.000 | 4068 | tags=39%, list=21%, signal=49% |
| 65 | KEGG\_AXON\_GUIDANCE |  | 114 | -0.19 | -0.19 | 0.000 | 0.232 | 1.000 | 10083 | tags=69%, list=51%, signal=140% |
| 66 | KEGG\_LONG\_TERM\_DEPRESSION |  | 49 | -0.18 | -0.18 | 0.076 | 0.245 | 1.000 | 3273 | tags=35%, list=17%, signal=41% |
| 67 | KEGG\_MAPK\_SIGNALING\_PATHWAY |  | 234 | -0.18 | -0.18 | 0.000 | 0.251 | 1.000 | 10195 | tags=69%, list=51%, signal=141% |
| 68 | KEGG\_VEGF\_SIGNALING\_PATHWAY |  | 71 | -0.18 | -0.18 | 0.017 | 0.260 | 1.000 | 10170 | tags=69%, list=51%, signal=141% |
| 69 | KEGG\_FATTY\_ACID\_METABOLISM |  | 41 | -0.18 | -0.18 | 0.155 | 0.264 | 1.000 | 12467 | tags=80%, list=63%, signal=216% |
| 70 | KEGG\_CALCIUM\_SIGNALING\_PATHWAY |  | 150 | -0.17 | -0.17 | 0.000 | 0.269 | 1.000 | 8324 | tags=59%, list=42%, signal=101% |
| 71 | KEGG\_VASCULAR\_SMOOTH\_MUSCLE\_CONTRACTION |  | 96 | -0.17 | -0.17 | 0.006 | 0.276 | 1.000 | 7743 | tags=56%, list=39%, signal=92% |
| 72 | KEGG\_FOCAL\_ADHESION |  | 189 | -0.17 | -0.17 | 0.000 | 0.297 | 1.000 | 10333 | tags=69%, list=52%, signal=142% |
| 73 | KEGG\_BETA\_ALANINE\_METABOLISM |  | 22 | -0.17 | -0.17 | 0.545 | 0.310 | 1.000 | 12947 | tags=82%, list=65%, signal=236% |
| 74 | KEGG\_HISTIDINE\_METABOLISM |  | 27 | -0.17 | -0.17 | 0.422 | 0.309 | 1.000 | 5546 | tags=44%, list=28%, signal=62% |
| 75 | KEGG\_PATHOGENIC\_ESCHERICHIA\_COLI\_INFECTION |  | 53 | -0.16 | -0.16 | 0.112 | 0.319 | 1.000 | 11001 | tags=72%, list=55%, signal=161% |
| 76 | KEGG\_ARGININE\_AND\_PROLINE\_METABOLISM |  | 49 | -0.16 | -0.16 | 0.153 | 0.339 | 1.000 | 10615 | tags=69%, list=54%, signal=149% |
| 77 | KEGG\_GLYCEROLIPID\_METABOLISM |  | 40 | -0.15 | -0.15 | 0.260 | 0.361 | 1.000 | 12300 | tags=78%, list=62%, signal=204% |
| 78 | KEGG\_ADIPOCYTOKINE\_SIGNALING\_PATHWAY |  | 67 | -0.15 | -0.15 | 0.090 | 0.383 | 1.000 | 15067 | tags=91%, list=76%, signal=378% |
| 79 | KEGG\_PPAR\_SIGNALING\_PATHWAY |  | 63 | -0.15 | -0.15 | 0.109 | 0.388 | 1.000 | 10259 | tags=67%, list=52%, signal=138% |
| 80 | KEGG\_ASCORBATE\_AND\_ALDARATE\_METABOLISM |  | 16 | -0.15 | -0.15 | 0.840 | 0.399 | 1.000 | 795 | tags=19%, list=4%, signal=20% |
| 81 | KEGG\_OXIDATIVE\_PHOSPHORYLATION |  | 100 | -0.15 | -0.15 | 0.030 | 0.394 | 1.000 | 14142 | tags=86%, list=71%, signal=298% |
| 82 | KEGG\_CHRONIC\_MYELOID\_LEUKEMIA |  | 72 | -0.15 | -0.15 | 0.083 | 0.390 | 1.000 | 12786 | tags=79%, list=64%, signal=222% |
| 83 | KEGG\_PORPHYRIN\_AND\_CHLOROPHYLL\_METABOLISM |  | 31 | -0.15 | -0.15 | 0.477 | 0.388 | 1.000 | 16918 | tags=100%, list=85%, signal=681% |
| 84 | KEGG\_SMALL\_CELL\_LUNG\_CANCER |  | 84 | -0.14 | -0.14 | 0.060 | 0.415 | 1.000 | 10637 | tags=68%, list=54%, signal=146% |
| 85 | KEGG\_HEDGEHOG\_SIGNALING\_PATHWAY |  | 54 | -0.14 | -0.14 | 0.208 | 0.416 | 1.000 | 11149 | tags=70%, list=56%, signal=160% |
| 86 | KEGG\_PROPANOATE\_METABOLISM |  | 31 | -0.14 | -0.14 | 0.518 | 0.421 | 1.000 | 13206 | tags=81%, list=67%, signal=241% |
| 87 | KEGG\_TGF\_BETA\_SIGNALING\_PATHWAY |  | 84 | -0.14 | -0.14 | 0.084 | 0.455 | 1.000 | 13374 | tags=81%, list=67%, signal=248% |
| 88 | KEGG\_PENTOSE\_AND\_GLUCURONATE\_INTERCONVERSIONS |  | 19 | -0.13 | -0.13 | 0.853 | 0.459 | 1.000 | 17163 | tags=100%, list=87%, signal=744% |
| 89 | KEGG\_AMYOTROPHIC\_LATERAL\_SCLEROSIS\_ALS |  | 52 | -0.13 | -0.13 | 0.285 | 0.461 | 1.000 | 9179 | tags=60%, list=46%, signal=111% |
| 90 | KEGG\_INOSITOL\_PHOSPHATE\_METABOLISM |  | 51 | -0.13 | -0.13 | 0.299 | 0.469 | 1.000 | 16440 | tags=96%, list=83%, signal=561% |
| 91 | KEGG\_NEUROACTIVE\_LIGAND\_RECEPTOR\_INTERACTION |  | 225 | -0.13 | -0.13 | 0.001 | 0.477 | 1.000 | 6256 | tags=44%, list=32%, signal=64% |
| 92 | KEGG\_REGULATION\_OF\_ACTIN\_CYTOSKELETON |  | 195 | -0.12 | -0.12 | 0.005 | 0.532 | 1.000 | 11001 | tags=68%, list=55%, signal=151% |
| 93 | KEGG\_ENDOCYTOSIS |  | 174 | -0.12 | -0.12 | 0.008 | 0.538 | 1.000 | 12191 | tags=74%, list=61%, signal=189% |
| 94 | KEGG\_NITROGEN\_METABOLISM |  | 21 | -0.12 | -0.12 | 0.895 | 0.539 | 1.000 | 2323 | tags=24%, list=12%, signal=27% |
| 95 | KEGG\_ACUTE\_MYELOID\_LEUKEMIA |  | 56 | -0.12 | -0.12 | 0.339 | 0.539 | 1.000 | 14615 | tags=86%, list=74%, signal=325% |
| 96 | KEGG\_NEUROTROPHIN\_SIGNALING\_PATHWAY |  | 122 | -0.12 | -0.12 | 0.048 | 0.542 | 1.000 | 10161 | tags=63%, list=51%, signal=129% |
| 97 | KEGG\_P53\_SIGNALING\_PATHWAY |  | 64 | -0.12 | -0.12 | 0.293 | 0.537 | 1.000 | 7554 | tags=50%, list=38%, signal=81% |
| 98 | KEGG\_VASOPRESSIN\_REGULATED\_WATER\_REABSORPTION |  | 41 | -0.12 | -0.12 | 0.571 | 0.539 | 1.000 | 16515 | tags=95%, list=83%, signal=568% |
| 99 | KEGG\_PATHWAYS\_IN\_CANCER |  | 308 | -0.12 | -0.12 | 0.000 | 0.538 | 1.000 | 10637 | tags=65%, list=54%, signal=139% |
| 100 | KEGG\_DRUG\_METABOLISM\_OTHER\_ENZYMES |  | 35 | -0.12 | -0.12 | 0.693 | 0.548 | 1.000 | 3932 | tags=31%, list=20%, signal=39% |
| 101 | KEGG\_NICOTINATE\_AND\_NICOTINAMIDE\_METABOLISM |  | 18 | -0.12 | -0.12 | 0.942 | 0.547 | 1.000 | 6520 | tags=44%, list=33%, signal=66% |
| 102 | KEGG\_MELANOGENESIS |  | 89 | -0.11 | -0.11 | 0.190 | 0.549 | 1.000 | 7089 | tags=47%, list=36%, signal=73% |
| 103 | KEGG\_BASAL\_CELL\_CARCINOMA |  | 53 | -0.11 | -0.11 | 0.519 | 0.592 | 1.000 | 6440 | tags=43%, list=32%, signal=64% |
| 104 | KEGG\_WNT\_SIGNALING\_PATHWAY |  | 146 | -0.11 | -0.11 | 0.063 | 0.591 | 1.000 | 11302 | tags=68%, list=57%, signal=157% |
| 105 | KEGG\_NON\_SMALL\_CELL\_LUNG\_CANCER |  | 54 | -0.11 | -0.11 | 0.542 | 0.620 | 1.000 | 12242 | tags=72%, list=62%, signal=188% |
| 106 | KEGG\_PANCREATIC\_CANCER |  | 69 | -0.10 | -0.10 | 0.416 | 0.616 | 1.000 | 14883 | tags=86%, list=75%, signal=342% |
| 107 | KEGG\_MELANOMA |  | 64 | -0.10 | -0.10 | 0.473 | 0.619 | 1.000 | 4144 | tags=31%, list=21%, signal=39% |
| 108 | KEGG\_NOTCH\_SIGNALING\_PATHWAY |  | 45 | -0.10 | -0.10 | 0.706 | 0.621 | 1.000 | 12064 | tags=71%, list=61%, signal=181% |
| 109 | KEGG\_GNRH\_SIGNALING\_PATHWAY |  | 84 | -0.10 | -0.10 | 0.316 | 0.631 | 1.000 | 16649 | tags=94%, list=84%, signal=584% |
| 110 | KEGG\_ERBB\_SIGNALING\_PATHWAY |  | 86 | -0.10 | -0.10 | 0.389 | 0.655 | 1.000 | 10290 | tags=62%, list=52%, signal=128% |
| 111 | KEGG\_COLORECTAL\_CANCER |  | 61 | -0.09 | -0.09 | 0.707 | 0.745 | 1.000 | 7060 | tags=44%, list=36%, signal=69% |
| 112 | KEGG\_GLIOMA |  | 62 | -0.08 | -0.08 | 0.749 | 0.767 | 1.000 | 11462 | tags=66%, list=58%, signal=156% |
| 113 | KEGG\_LONG\_TERM\_POTENTIATION |  | 63 | -0.06 | -0.06 | 0.950 | 0.921 | 1.000 | 17029 | tags=92%, list=86%, signal=650% |
| 114 | KEGG\_INSULIN\_SIGNALING\_PATHWAY |  | 133 | -0.05 | -0.05 | 0.822 | 0.953 | 1.000 | 15036 | tags=81%, list=76%, signal=334% |
Table: Gene sets enriched in phenotype **na**[plain text format]****

  
