# Supplementary material for: DNA methylation-based classifier and gene expression signatures detect BRCAness in osteosarcoma
Source: PLoS Comput Biol. 2021 Nov 11;17(11):e1009562. doi: 10.1371/journal.pcbi.1009562 (PMC8584788; doi:10.1371/journal.pcbi.1009562)
Supplement: S2 File — (ZIP) [file pcbi.1009562.s002.zip › S2_File/my_analysis_Kegg.GseaPreranked.1581692187239/gsea_report_for_na_pos_1581692187239.html]

Report for na\_pos 1581692187239 [GSEA]

| GS  follow link to MSigDB | GS DETAILS | SIZE | ES | NES | NOM p-val | FDR q-val | FWER p-val | RANK AT MAX | LEADING EDGE || 1 | KEGG\_DNA\_REPLICATION | Details ... | 30 | 0.64 | 0.64 | 0.000 | 0.000 | 0.000 | 6513 | tags=97%, list=33%, signal=144% |
| 2 | KEGG\_MISMATCH\_REPAIR | Details ... | 22 | 0.49 | 0.49 | 0.000 | 0.001 | 0.003 | 6513 | tags=82%, list=33%, signal=122% |
| 3 | KEGG\_HOMOLOGOUS\_RECOMBINATION | Details ... | 26 | 0.48 | 0.48 | 0.000 | 0.001 | 0.004 | 7233 | tags=85%, list=36%, signal=133% |
| 4 | KEGG\_ONE\_CARBON\_POOL\_BY\_FOLATE | Details ... | 17 | 0.43 | 0.43 | 0.001 | 0.004 | 0.023 | 10201 | tags=94%, list=51%, signal=194% |
| 5 | KEGG\_RNA\_POLYMERASE | Details ... | 28 | 0.43 | 0.43 | 0.000 | 0.003 | 0.023 | 7850 | tags=82%, list=40%, signal=136% |
| 6 | KEGG\_NUCLEOTIDE\_EXCISION\_REPAIR | Details ... | 42 | 0.38 | 0.38 | 0.000 | 0.009 | 0.078 | 7018 | tags=74%, list=35%, signal=114% |
| 7 | KEGG\_GLYCOSYLPHOSPHATIDYLINOSITOL\_GPI\_ANCHOR\_BIOSYNTHESIS | Details ... | 24 | 0.37 | 0.37 | 0.001 | 0.012 | 0.114 | 9148 | tags=83%, list=46%, signal=155% |
| 8 | KEGG\_PROTEASOME | Details ... | 43 | 0.36 | 0.36 | 0.000 | 0.015 | 0.162 | 9503 | tags=84%, list=48%, signal=160% |
| 9 | KEGG\_RIBOSOME | Details ... | 84 | 0.36 | 0.36 | 0.000 | 0.015 | 0.179 | 10444 | tags=88%, list=53%, signal=185% |
| 10 | KEGG\_AMINOACYL\_TRNA\_BIOSYNTHESIS | Details ... | 38 | 0.34 | 0.34 | 0.000 | 0.018 | 0.234 | 11436 | tags=92%, list=58%, signal=217% |
| 11 | KEGG\_BASE\_EXCISION\_REPAIR | Details ... | 32 | 0.34 | 0.34 | 0.001 | 0.019 | 0.262 | 6296 | tags=66%, list=32%, signal=96% |
| 12 | KEGG\_PROTEIN\_EXPORT | Details ... | 22 | 0.34 | 0.34 | 0.008 | 0.018 | 0.269 | 12229 | tags=95%, list=62%, signal=249% |
| 13 | KEGG\_BASAL\_TRANSCRIPTION\_FACTORS | Details ... | 32 | 0.33 | 0.33 | 0.001 | 0.022 | 0.342 | 8385 | tags=75%, list=42%, signal=130% |
| 14 | KEGG\_SPLICEOSOME | Details ... | 123 | 0.31 | 0.31 | 0.000 | 0.031 | 0.473 | 8703 | tags=75%, list=44%, signal=132% |
| 15 | KEGG\_N\_GLYCAN\_BIOSYNTHESIS | Details ... | 45 | 0.30 | 0.30 | 0.000 | 0.038 | 0.561 | 10803 | tags=84%, list=54%, signal=185% |
| 16 | KEGG\_CELL\_CYCLE | Details ... | 110 | 0.29 | 0.29 | 0.000 | 0.045 | 0.652 | 11762 | tags=88%, list=59%, signal=216% |
| 17 | KEGG\_FRUCTOSE\_AND\_MANNOSE\_METABOLISM | Details ... | 33 | 0.26 | 0.26 | 0.016 | 0.080 | 0.863 | 4975 | tags=52%, list=25%, signal=69% |
| 18 | KEGG\_PYRIMIDINE\_METABOLISM | Details ... | 97 | 0.26 | 0.26 | 0.000 | 0.082 | 0.884 | 8952 | tags=71%, list=45%, signal=129% |
| 19 | KEGG\_GLYCOSAMINOGLYCAN\_BIOSYNTHESIS\_CHONDROITIN\_SULFATE | Details ... | 20 | 0.25 | 0.25 | 0.121 | 0.095 | 0.929 | 14830 | tags=100%, list=75%, signal=396% |
| 20 | KEGG\_RNA\_DEGRADATION | Details ... | 53 | 0.24 | 0.24 | 0.003 | 0.127 | 0.977 | 13634 | tags=92%, list=69%, signal=295% |
| 21 | KEGG\_PENTOSE\_PHOSPHATE\_PATHWAY |  | 26 | 0.23 | 0.23 | 0.098 | 0.158 | 0.992 | 11548 | tags=81%, list=58%, signal=193% |
| 22 | KEGG\_TERPENOID\_BACKBONE\_BIOSYNTHESIS |  | 15 | 0.22 | 0.22 | 0.419 | 0.167 | 0.995 | 12808 | tags=87%, list=65%, signal=245% |
| 23 | KEGG\_LYSINE\_DEGRADATION |  | 38 | 0.21 | 0.21 | 0.055 | 0.187 | 0.999 | 10902 | tags=76%, list=55%, signal=169% |
| 24 | KEGG\_SELENOAMINO\_ACID\_METABOLISM |  | 25 | 0.19 | 0.19 | 0.278 | 0.281 | 1.000 | 6496 | tags=52%, list=33%, signal=77% |
| 25 | KEGG\_CITRATE\_CYCLE\_TCA\_CYCLE |  | 29 | 0.19 | 0.19 | 0.232 | 0.291 | 1.000 | 11985 | tags=79%, list=60%, signal=200% |
| 26 | KEGG\_GLYCOSAMINOGLYCAN\_BIOSYNTHESIS\_KERATAN\_SULFATE |  | 15 | 0.19 | 0.19 | 0.609 | 0.282 | 1.000 | 10802 | tags=73%, list=54%, signal=161% |
| 27 | KEGG\_BUTANOATE\_METABOLISM |  | 32 | 0.18 | 0.18 | 0.226 | 0.321 | 1.000 | 10676 | tags=72%, list=54%, signal=155% |
| 28 | KEGG\_HUNTINGTONS\_DISEASE |  | 148 | 0.18 | 0.18 | 0.000 | 0.328 | 1.000 | 14185 | tags=89%, list=72%, signal=311% |
| 29 | KEGG\_REGULATION\_OF\_AUTOPHAGY |  | 21 | 0.17 | 0.17 | 0.512 | 0.351 | 1.000 | 14524 | tags=90%, list=73%, signal=338% |
| 30 | KEGG\_GLYCINE\_SERINE\_AND\_THREONINE\_METABOLISM |  | 30 | 0.17 | 0.17 | 0.300 | 0.340 | 1.000 | 4520 | tags=40%, list=23%, signal=52% |
| 31 | KEGG\_SNARE\_INTERACTIONS\_IN\_VESICULAR\_TRANSPORT |  | 33 | 0.17 | 0.17 | 0.232 | 0.340 | 1.000 | 825 | tags=21%, list=4%, signal=22% |
| 32 | KEGG\_GLYCOSAMINOGLYCAN\_BIOSYNTHESIS\_HEPARAN\_SULFATE |  | 25 | 0.17 | 0.17 | 0.415 | 0.337 | 1.000 | 2986 | tags=32%, list=15%, signal=38% |
| 33 | KEGG\_PARKINSONS\_DISEASE |  | 99 | 0.17 | 0.17 | 0.007 | 0.345 | 1.000 | 11929 | tags=77%, list=60%, signal=192% |
| 34 | KEGG\_TASTE\_TRANSDUCTION |  | 35 | 0.16 | 0.16 | 0.292 | 0.351 | 1.000 | 9773 | tags=66%, list=49%, signal=129% |
| 35 | KEGG\_PURINE\_METABOLISM |  | 148 | 0.16 | 0.16 | 0.000 | 0.401 | 1.000 | 7646 | tags=54%, list=39%, signal=87% |
| 36 | KEGG\_OLFACTORY\_TRANSDUCTION |  | 111 | 0.15 | 0.15 | 0.013 | 0.430 | 1.000 | 10602 | tags=68%, list=53%, signal=146% |
| 37 | KEGG\_GALACTOSE\_METABOLISM |  | 25 | 0.15 | 0.15 | 0.560 | 0.427 | 1.000 | 12104 | tags=76%, list=61%, signal=195% |
| 38 | KEGG\_CYSTEINE\_AND\_METHIONINE\_METABOLISM |  | 33 | 0.14 | 0.14 | 0.444 | 0.468 | 1.000 | 15787 | tags=94%, list=80%, signal=460% |
| 39 | KEGG\_GLUTATHIONE\_METABOLISM |  | 46 | 0.13 | 0.13 | 0.347 | 0.533 | 1.000 | 5092 | tags=39%, list=26%, signal=53% |
| 40 | KEGG\_OOCYTE\_MEIOSIS |  | 98 | 0.13 | 0.13 | 0.059 | 0.528 | 1.000 | 6260 | tags=45%, list=32%, signal=65% |
| 41 | KEGG\_MTOR\_SIGNALING\_PATHWAY |  | 49 | 0.13 | 0.13 | 0.359 | 0.536 | 1.000 | 13988 | tags=84%, list=71%, signal=283% |
| 42 | KEGG\_CARDIAC\_MUSCLE\_CONTRACTION |  | 62 | 0.13 | 0.13 | 0.230 | 0.531 | 1.000 | 14686 | tags=87%, list=74%, signal=335% |
| 43 | KEGG\_VALINE\_LEUCINE\_AND\_ISOLEUCINE\_DEGRADATION |  | 43 | 0.13 | 0.13 | 0.415 | 0.520 | 1.000 | 10790 | tags=67%, list=54%, signal=148% |
| 44 | KEGG\_SYSTEMIC\_LUPUS\_ERYTHEMATOSUS |  | 114 | 0.13 | 0.13 | 0.047 | 0.513 | 1.000 | 1265 | tags=19%, list=6%, signal=20% |
| 45 | KEGG\_PYRUVATE\_METABOLISM |  | 39 | 0.13 | 0.13 | 0.475 | 0.503 | 1.000 | 15734 | tags=92%, list=79%, signal=446% |
| 46 | KEGG\_THYROID\_CANCER |  | 29 | 0.13 | 0.13 | 0.695 | 0.516 | 1.000 | 16630 | tags=97%, list=84%, signal=598% |
| 47 | KEGG\_GLYCOLYSIS\_GLUCONEOGENESIS |  | 60 | 0.12 | 0.12 | 0.292 | 0.522 | 1.000 | 3147 | tags=28%, list=16%, signal=34% |
| 48 | KEGG\_ALANINE\_ASPARTATE\_AND\_GLUTAMATE\_METABOLISM |  | 31 | 0.12 | 0.12 | 0.722 | 0.568 | 1.000 | 14282 | tags=84%, list=72%, signal=299% |
| 49 | KEGG\_PROGESTERONE\_MEDIATED\_OOCYTE\_MATURATION |  | 70 | 0.12 | 0.12 | 0.264 | 0.566 | 1.000 | 6175 | tags=43%, list=31%, signal=62% |
| 50 | KEGG\_AMINO\_SUGAR\_AND\_NUCLEOTIDE\_SUGAR\_METABOLISM |  | 39 | 0.11 | 0.11 | 0.716 | 0.641 | 1.000 | 4975 | tags=36%, list=25%, signal=48% |
| 51 | KEGG\_BLADDER\_CANCER |  | 40 | 0.11 | 0.11 | 0.723 | 0.647 | 1.000 | 16237 | tags=93%, list=82%, signal=510% |
| 52 | KEGG\_STARCH\_AND\_SUCROSE\_METABOLISM |  | 37 | 0.10 | 0.10 | 0.782 | 0.655 | 1.000 | 11336 | tags=68%, list=57%, signal=157% |
| 53 | KEGG\_UBIQUITIN\_MEDIATED\_PROTEOLYSIS |  | 120 | 0.10 | 0.10 | 0.159 | 0.650 | 1.000 | 12835 | tags=75%, list=65%, signal=211% |
| 54 | KEGG\_PROSTATE\_CANCER |  | 86 | 0.10 | 0.10 | 0.322 | 0.662 | 1.000 | 13458 | tags=78%, list=68%, signal=241% |
| 55 | KEGG\_PEROXISOME |  | 76 | 0.10 | 0.10 | 0.441 | 0.667 | 1.000 | 13700 | tags=79%, list=69%, signal=254% |
| 56 | KEGG\_ALZHEIMERS\_DISEASE |  | 137 | 0.10 | 0.10 | 0.140 | 0.658 | 1.000 | 14559 | tags=83%, list=73%, signal=311% |
| 57 | KEGG\_ENDOMETRIAL\_CANCER |  | 49 | 0.09 | 0.09 | 0.766 | 0.687 | 1.000 | 13922 | tags=80%, list=70%, signal=267% |
| 58 | KEGG\_GAP\_JUNCTION |  | 77 | 0.07 | 0.07 | 0.827 | 0.881 | 1.000 | 5845 | tags=36%, list=29%, signal=51% |
| 59 | KEGG\_RENAL\_CELL\_CARCINOMA |  | 65 | 0.06 | 0.06 | 0.939 | 0.901 | 1.000 | 13988 | tags=77%, list=71%, signal=260% |
Table: Gene sets enriched in phenotype **na**[plain text format]****

  
