# Supplementary material for: DNA methylation-based classifier and gene expression signatures detect BRCAness in osteosarcoma
Source: PLoS Comput Biol. 2021 Nov 11;17(11):e1009562. doi: 10.1371/journal.pcbi.1009562 (PMC8584788; doi:10.1371/journal.pcbi.1009562)
Supplement: S2 File — (ZIP) [file pcbi.1009562.s002.zip › S2_File/my_analysis_Kegg.GseaPreranked.1581692187239/index.html]

Index for xtools.gsea.GseaPreranked my\_analysis\_Kegg.GseaPreranked.1581692187239

### GSEA Report for Dataset DEG3\_two3dTopBottom

#### Enrichment in phenotype: **na**

- 59 / 173 gene sets are upregulated in phenotype **na\_pos**- 23 gene sets are significant at FDR < 25%- 21 gene sets are significantly enriched at nominal pvalue < 1%- 24 gene sets are significantly enriched at nominal pvalue < 5%- Snapshot of enrichment results- Detailed enrichment results in html format- Detailed enrichment results in excel format (tab delimited text)- Guide to interpret results

#### Enrichment in phenotype: **na**

- 114 / 173 gene sets are upregulated in phenotype **na\_neg**- 66 gene sets are significantly enriched at FDR < 25%- 55 gene sets are significantly enriched at nominal pvalue < 1%- 65 gene sets are significantly enriched at nominal pvalue < 5%- Snapshot of enrichment results- Detailed enrichment results in html format- Detailed enrichment results in excel format (tab delimited text)- Guide to interpret results

#### Dataset details

- The dataset has 19827 features (genes)- No probe set => gene symbol collapsing was requested, so all 19827 features were used

#### Gene set details

- Gene set size filters (min=15, max=500) resulted in filtering out 13 / 186 gene sets- The remaining 173 gene sets were used in the analysis- List of gene sets used and their sizes (restricted to features in the specified dataset)

#### Gene markers for the **na\_pos** *versus* **na\_neg** comparison

- The dataset has 19827 features (genes)- Detailed rank ordered gene list for all features in the dataset

#### Global statistics and plots

- Plot of p-values *vs.* NES- Global ES histogram

#### Other

- Parameters used for this analysis

#### Comments

- Timestamp used as the random seed: 1581692189263

---

Report: my\_analysis\_Kegg.GseaPreranked.1581692187239.rpt   by user: maxim

xtools.gsea.GseaPreranked [Fri, Feb 14, '20 3 PM 56]

Website: www.gsea-msigdb.org/gsea
Questions & Suggestions: Contact page
