# Supplementary material for: DNA methylation-based classifier and gene expression signatures detect BRCAness in osteosarcoma
Source: PLoS Comput Biol. 2021 Nov 11;17(11):e1009562. doi: 10.1371/journal.pcbi.1009562 (PMC8584788; doi:10.1371/journal.pcbi.1009562)
Supplement: S2 File — (ZIP) [file pcbi.1009562.s002.zip › S2_File/my_analysis_Kegg.GseaPreranked.1581692187239/KEGG_ALLOGRAFT_REJECTION.html]

Details for gene set KEGG\_ALLOGRAFT\_REJECTION[GSEA]

|  || Dataset | DEG3\_two3dTopBottom |
| Phenotype | NoPhenotypeAvailable |
| Upregulated in class | na\_neg |
| GeneSet | KEGG\_ALLOGRAFT\_REJECTION |
| Enrichment Score (ES) | -0.6693121 |
| Normalized Enrichment Score (NES) | -0.6693121 |
| Nominal p-value | 0.0 |
| FDR q-value | 0.0 |
| FWER p-Value | 0.0 |
Table: GSEA Results Summary

  

Fig 1: Enrichment plot: KEGG\_ALLOGRAFT\_REJECTION      
 Profile of the Running ES Score & Positions of GeneSet Members on the Rank Ordered List

  

| PROBE | GENE SYMBOL | GENE\_TITLE | RANK IN GENE LIST | RANK METRIC SCORE | RUNNING ES | CORE ENRICHMENT || 1 | HLA-C |  |  | 10192 | 1.248 | -0.4846 | No |
| 2 | HLA-A |  |  | 13593 | -1.494 | -0.6261 | No |
| 3 | CD86 |  |  | 14450 | -1.967 | -0.6390 | Yes |
| 4 | IL2 |  |  | 14536 | -2.032 | -0.6130 | Yes |
| 5 | HLA-B |  |  | 14983 | -2.404 | -0.6052 | Yes |
| 6 | IL12A |  |  | 15711 | -3.497 | -0.6117 | Yes |
| 7 | HLA-F |  |  | 16065 | -4.220 | -0.5992 | Yes |
| 8 | CD28 |  |  | 16514 | -5.798 | -0.5915 | Yes |
| 9 | HLA-G |  |  | 16786 | -7.343 | -0.5749 | Yes |
| 10 | TNF |  |  | 16858 | -7.878 | -0.5482 | Yes |
| 11 | CD40 |  |  | 17248 | -11.890 | -0.5375 | Yes |
| 12 | IL12B |  |  | 17602 | -18.740 | -0.5251 | Yes |
| 13 | GZMB |  |  | 17776 | -25.560 | -0.5035 | Yes |
| 14 | HLA-DQA2 |  |  | 17900 | -31.160 | -0.4794 | Yes |
| 15 | FAS |  |  | 18057 | -41.750 | -0.4570 | Yes |
| 16 | HLA-DPB1 |  |  | 18263 | -63.760 | -0.4370 | Yes |
| 17 | HLA-DPA1 |  |  | 18376 | -82.260 | -0.4124 | Yes |
| 18 | HLA-DMA |  |  | 18388 | -85.320 | -0.3827 | Yes |
| 19 | HLA-DRB1 |  |  | 18392 | -86.860 | -0.3525 | Yes |
| 20 | HLA-DOA |  |  | 18426 | -97.930 | -0.3239 | Yes |
| 21 | HLA-DMB |  |  | 18587 | -159.700 | -0.3016 | Yes |
| 22 | HLA-E |  |  | 18671 | -203.600 | -0.2755 | Yes |
| 23 | HLA-DRA |  |  | 18720 | -232.200 | -0.2477 | Yes |
| 24 | IL10 |  |  | 18725 | -237.700 | -0.2176 | Yes |
| 25 | HLA-DRB5 |  |  | 18733 | -243.500 | -0.1876 | Yes |
| 26 | HLA-DOB |  |  | 18766 | -268.900 | -0.1589 | Yes |
| 27 | CD80 |  |  | 18879 | -411.400 | -0.1343 | Yes |
| 28 | HLA-DQA1 |  |  | 18983 | -673.000 | -0.1092 | Yes |
| 29 | HLA-DQB1 |  |  | 19125 | -1584.000 | -0.0860 | Yes |
| 30 | FASLG |  |  | 19371 | -10130.000 | -0.0681 | Yes |
| 31 | PRF1 |  |  | 19503 | -62340.000 | -0.0444 | Yes |
| 32 | CD40LG |  |  | 19510 | -66050.000 | -0.0144 | Yes |
| 33 | IFNG |  |  | 19720 | -4827000.000 | 0.0054 | Yes |
Table: GSEA details [plain text format]

  

Fig 2: KEGG\_ALLOGRAFT\_REJECTION: Random ES distribution      
 Gene set null distribution of ES for **KEGG\_ALLOGRAFT\_REJECTION**

  
