# Supplementary material for: DNA methylation-based classifier and gene expression signatures detect BRCAness in osteosarcoma
Source: PLoS Comput Biol. 2021 Nov 11;17(11):e1009562. doi: 10.1371/journal.pcbi.1009562 (PMC8584788; doi:10.1371/journal.pcbi.1009562)
Supplement: S2 File — (ZIP) [file pcbi.1009562.s002.zip › S2_File/my_analysis_Kegg.GseaPreranked.1581692187239/KEGG_AMINOACYL_TRNA_BIOSYNTHESIS.html]

Details for gene set KEGG\_AMINOACYL\_TRNA\_BIOSYNTHESIS[GSEA]

|  || Dataset | DEG3\_two3dTopBottom |
| Phenotype | NoPhenotypeAvailable |
| Upregulated in class | na\_pos |
| GeneSet | KEGG\_AMINOACYL\_TRNA\_BIOSYNTHESIS |
| Enrichment Score (ES) | 0.34487396 |
| Normalized Enrichment Score (NES) | 0.34487396 |
| Nominal p-value | 0.0 |
| FDR q-value | 0.01824286 |
| FWER p-Value | 0.23366667 |
Table: GSEA Results Summary

  

Fig 1: Enrichment plot: KEGG\_AMINOACYL\_TRNA\_BIOSYNTHESIS      
 Profile of the Running ES Score & Positions of GeneSet Members on the Rank Ordered List

  

| PROBE | GENE SYMBOL | GENE\_TITLE | RANK IN GENE LIST | RANK METRIC SCORE | RUNNING ES | CORE ENRICHMENT || 1 | AARS2 |  |  | 89 | 6286.000 | 0.0218 | Yes |
| 2 | SARS |  |  | 566 | 157.300 | 0.0241 | Yes |
| 3 | YARS2 |  |  | 1154 | 43.130 | 0.0207 | Yes |
| 4 | TARS |  |  | 1179 | 42.360 | 0.0458 | Yes |
| 5 | PARS2 |  |  | 1593 | 24.900 | 0.0513 | Yes |
| 6 | GARS |  |  | 1837 | 19.850 | 0.0653 | Yes |
| 7 | FARSA |  |  | 1998 | 17.680 | 0.0835 | Yes |
| 8 | CARS2 |  |  | 2079 | 16.720 | 0.1058 | Yes |
| 9 | CARS |  |  | 2656 | 11.340 | 0.1030 | Yes |
| 10 | TARSL2 |  |  | 2699 | 11.020 | 0.1272 | Yes |
| 11 | EARS2 |  |  | 2910 | 9.939 | 0.1429 | Yes |
| 12 | VARS |  |  | 3322 | 7.941 | 0.1485 | Yes |
| 13 | RARS |  |  | 3408 | 7.596 | 0.1705 | Yes |
| 14 | FARSB |  |  | 3513 | 7.304 | 0.1916 | Yes |
| 15 | KARS |  |  | 4005 | 5.950 | 0.1931 | Yes |
| 16 | DARS |  |  | 4181 | 5.589 | 0.2105 | Yes |
| 17 | SEPSECS |  |  | 4199 | 5.558 | 0.2360 | Yes |
| 18 | WARS2 |  |  | 4336 | 5.279 | 0.2554 | Yes |
| 19 | MTFMT |  |  | 4525 | 4.939 | 0.2722 | Yes |
| 20 | EPRS |  |  | 4959 | 4.257 | 0.2767 | Yes |
| 21 | IARS |  |  | 4963 | 4.246 | 0.3028 | Yes |
| 22 | FARS2 |  |  | 5137 | 4.038 | 0.3204 | Yes |
| 23 | HARS |  |  | 5489 | 3.593 | 0.3290 | Yes |
| 24 | LARS |  |  | 6704 | 2.590 | 0.2940 | Yes |
| 25 | AARS |  |  | 7244 | 2.269 | 0.2930 | Yes |
| 26 | VARS2 |  |  | 7247 | 2.269 | 0.3193 | Yes |
| 27 | YARS |  |  | 7440 | 2.180 | 0.3359 | Yes |
| 28 | QARS |  |  | 7927 | 1.959 | 0.3376 | Yes |
| 29 | SARS2 |  |  | 9040 | 1.542 | 0.3078 | Yes |
| 30 | MARS2 |  |  | 9627 | 1.379 | 0.3045 | Yes |
| 31 | MARS |  |  | 9908 | 1.313 | 0.3166 | Yes |
| 32 | DARS2 |  |  | 9964 | 1.298 | 0.3402 | Yes |
| 33 | IARS2 |  |  | 10772 | 1.141 | 0.3257 | Yes |
| 34 | HARS2 |  |  | 11402 | 1.027 | 0.3202 | Yes |
| 35 | PSTK |  |  | 11436 | 1.022 | 0.3449 | Yes |
| 36 | RARS2 |  |  | 14367 | -1.909 | 0.2231 | No |
| 37 | LARS2 |  |  | 15010 | -2.423 | 0.2170 | No |
| 38 | WARS |  |  | 18036 | -39.720 | 0.0905 | No |
Table: GSEA details [plain text format]

  

Fig 2: KEGG\_AMINOACYL\_TRNA\_BIOSYNTHESIS: Random ES distribution      
 Gene set null distribution of ES for **KEGG\_AMINOACYL\_TRNA\_BIOSYNTHESIS**

  
