# Supplementary material for: DNA methylation-based classifier and gene expression signatures detect BRCAness in osteosarcoma
Source: PLoS Comput Biol. 2021 Nov 11;17(11):e1009562. doi: 10.1371/journal.pcbi.1009562 (PMC8584788; doi:10.1371/journal.pcbi.1009562)
Supplement: S2 File — (ZIP) [file pcbi.1009562.s002.zip › S2_File/my_analysis_Kegg.GseaPreranked.1581692187239/KEGG_ANTIGEN_PROCESSING_AND_PRESENTATION.html]

Details for gene set KEGG\_ANTIGEN\_PROCESSING\_AND\_PRESENTATION[GSEA]

|  || Dataset | DEG3\_two3dTopBottom |
| Phenotype | NoPhenotypeAvailable |
| Upregulated in class | na\_neg |
| GeneSet | KEGG\_ANTIGEN\_PROCESSING\_AND\_PRESENTATION |
| Enrichment Score (ES) | -0.3877788 |
| Normalized Enrichment Score (NES) | -0.3877788 |
| Nominal p-value | 0.0 |
| FDR q-value | 0.006855575 |
| FWER p-Value | 0.07433333 |
Table: GSEA Results Summary

  

Fig 1: Enrichment plot: KEGG\_ANTIGEN\_PROCESSING\_AND\_PRESENTATION      
 Profile of the Running ES Score & Positions of GeneSet Members on the Rank Ordered List

  

| PROBE | GENE SYMBOL | GENE\_TITLE | RANK IN GENE LIST | RANK METRIC SCORE | RUNNING ES | CORE ENRICHMENT || 1 | HSP90AB1 |  |  | 218 | 1057.000 | 0.0046 | No |
| 2 | NFYA |  |  | 1770 | 21.050 | -0.0583 | No |
| 3 | PSME3 |  |  | 2457 | 12.960 | -0.0773 | No |
| 4 | PSME2 |  |  | 5078 | 4.111 | -0.1943 | No |
| 5 | RFXANK |  |  | 5150 | 4.021 | -0.1823 | No |
| 6 | KLRC2 |  |  | 5239 | 3.885 | -0.1711 | No |
| 7 | HSPA5 |  |  | 5389 | 3.722 | -0.1630 | No |
| 8 | CANX |  |  | 6099 | 3.014 | -0.1833 | No |
| 9 | HSPA4 |  |  | 6218 | 2.922 | -0.1736 | No |
| 10 | CALR |  |  | 7324 | 2.231 | -0.2139 | No |
| 11 | RFXAP |  |  | 7562 | 2.118 | -0.2103 | No |
| 12 | HSP90AA1 |  |  | 7983 | 1.934 | -0.2159 | No |
| 13 | PDIA3 |  |  | 8203 | 1.829 | -0.2113 | No |
| 14 | PSME1 |  |  | 8874 | 1.596 | -0.2296 | No |
| 15 | KLRC3 |  |  | 9936 | 1.307 | -0.2677 | No |
| 16 | HLA-C |  |  | 10192 | 1.248 | -0.2650 | No |
| 17 | HSPA8 |  |  | 10247 | 1.235 | -0.2521 | No |
| 18 | NFYC |  |  | 12026 | -1.074 | -0.3264 | No |
| 19 | NFYB |  |  | 12148 | -1.096 | -0.3169 | No |
| 20 | CREB1 |  |  | 13077 | -1.315 | -0.3482 | No |
| 21 | RFX5 |  |  | 13260 | -1.366 | -0.3418 | No |
| 22 | CTSL |  |  | 13440 | -1.432 | -0.3352 | No |
| 23 | HLA-A |  |  | 13593 | -1.494 | -0.3273 | No |
| 24 | TAP1 |  |  | 14789 | -2.214 | -0.3722 | Yes |
| 25 | HLA-B |  |  | 14983 | -2.404 | -0.3663 | Yes |
| 26 | TAPBP |  |  | 15251 | -2.742 | -0.3642 | Yes |
| 27 | TAP2 |  |  | 15612 | -3.293 | -0.3668 | Yes |
| 28 | KLRC4 |  |  | 15800 | -3.655 | -0.3606 | Yes |
| 29 | HLA-F |  |  | 16065 | -4.220 | -0.3583 | Yes |
| 30 | CTSB |  |  | 16611 | -6.223 | -0.3703 | Yes |
| 31 | HLA-G |  |  | 16786 | -7.343 | -0.3635 | Yes |
| 32 | B2M |  |  | 16830 | -7.668 | -0.3500 | Yes |
| 33 | LGMN |  |  | 17038 | -9.523 | -0.3449 | Yes |
| 34 | CD74 |  |  | 17341 | -13.480 | -0.3445 | Yes |
| 35 | HSPA6 |  |  | 17604 | -18.840 | -0.3422 | Yes |
| 36 | LTA |  |  | 17687 | -21.740 | -0.3307 | Yes |
| 37 | KIR2DL3 |  |  | 17725 | -23.460 | -0.3169 | Yes |
| 38 | HLA-DQA2 |  |  | 17900 | -31.160 | -0.3101 | Yes |
| 39 | IFI30 |  |  | 18000 | -37.010 | -0.2995 | Yes |
| 40 | HSPA1A |  |  | 18005 | -37.190 | -0.2841 | Yes |
| 41 | HSPA1B |  |  | 18058 | -41.810 | -0.2711 | Yes |
| 42 | HSPA1L |  |  | 18060 | -41.890 | -0.2555 | Yes |
| 43 | KIR3DL1 |  |  | 18098 | -44.440 | -0.2418 | Yes |
| 44 | CIITA |  |  | 18136 | -48.460 | -0.2280 | Yes |
| 45 | HLA-DPB1 |  |  | 18263 | -63.760 | -0.2187 | Yes |
| 46 | HLA-DPA1 |  |  | 18376 | -82.260 | -0.2088 | Yes |
| 47 | HLA-DMA |  |  | 18388 | -85.320 | -0.1937 | Yes |
| 48 | HLA-DRB1 |  |  | 18392 | -86.860 | -0.1782 | Yes |
| 49 | HLA-DOA |  |  | 18426 | -97.930 | -0.1643 | Yes |
| 50 | HLA-DMB |  |  | 18587 | -159.700 | -0.1568 | Yes |
| 51 | HLA-E |  |  | 18671 | -203.600 | -0.1453 | Yes |
| 52 | HLA-DRA |  |  | 18720 | -232.200 | -0.1321 | Yes |
| 53 | HLA-DRB5 |  |  | 18733 | -243.500 | -0.1171 | Yes |
| 54 | KIR3DL2 |  |  | 18738 | -246.800 | -0.1017 | Yes |
| 55 | HLA-DOB |  |  | 18766 | -268.900 | -0.0874 | Yes |
| 56 | CD4 |  |  | 18780 | -283.000 | -0.0725 | Yes |
| 57 | CD8B |  |  | 18793 | -289.900 | -0.0575 | Yes |
| 58 | KIR2DS4 |  |  | 18884 | -424.000 | -0.0464 | Yes |
| 59 | HLA-DQA1 |  |  | 18983 | -673.000 | -0.0357 | Yes |
| 60 | HLA-DQB1 |  |  | 19125 | -1584.000 | -0.0272 | Yes |
| 61 | CTSS |  |  | 19389 | -12090.000 | -0.0249 | Yes |
| 62 | KLRC1 |  |  | 19464 | -31380.000 | -0.0130 | Yes |
| 63 | CD8A |  |  | 19544 | -106100.000 | -0.0014 | Yes |
| 64 | KLRD1 |  |  | 19787 | -179400000.000 | 0.0020 | Yes |
Table: GSEA details [plain text format]

  

Fig 2: KEGG\_ANTIGEN\_PROCESSING\_AND\_PRESENTATION: Random ES distribution      
 Gene set null distribution of ES for **KEGG\_ANTIGEN\_PROCESSING\_AND\_PRESENTATION**

  
