# Supplementary material for: DNA methylation-based classifier and gene expression signatures detect BRCAness in osteosarcoma
Source: PLoS Comput Biol. 2021 Nov 11;17(11):e1009562. doi: 10.1371/journal.pcbi.1009562 (PMC8584788; doi:10.1371/journal.pcbi.1009562)
Supplement: S2 File — (ZIP) [file pcbi.1009562.s002.zip › S2_File/my_analysis_Kegg.GseaPreranked.1581692187239/KEGG_ASTHMA.html]

Details for gene set KEGG\_ASTHMA[GSEA]

|  || Dataset | DEG3\_two3dTopBottom |
| Phenotype | NoPhenotypeAvailable |
| Upregulated in class | na\_neg |
| GeneSet | KEGG\_ASTHMA |
| Enrichment Score (ES) | -0.70485324 |
| Normalized Enrichment Score (NES) | -0.70485324 |
| Nominal p-value | 0.0 |
| FDR q-value | 0.0 |
| FWER p-Value | 0.0 |
Table: GSEA Results Summary

  

Fig 1: Enrichment plot: KEGG\_ASTHMA      
 Profile of the Running ES Score & Positions of GeneSet Members on the Rank Ordered List

  

| PROBE | GENE SYMBOL | GENE\_TITLE | RANK IN GENE LIST | RANK METRIC SCORE | RUNNING ES | CORE ENRICHMENT || 1 | EPX |  |  | 9826 | 1.332 | -0.4527 | No |
| 2 | PRG2 |  |  | 14430 | -1.954 | -0.6416 | No |
| 3 | FCER1G |  |  | 15683 | -3.439 | -0.6614 | Yes |
| 4 | IL13 |  |  | 15799 | -3.655 | -0.6237 | Yes |
| 5 | TNF |  |  | 16858 | -7.878 | -0.6336 | Yes |
| 6 | CD40 |  |  | 17248 | -11.890 | -0.6098 | Yes |
| 7 | HLA-DQA2 |  |  | 17900 | -31.160 | -0.5992 | Yes |
| 8 | HLA-DPB1 |  |  | 18263 | -63.760 | -0.5740 | Yes |
| 9 | FCER1A |  |  | 18300 | -68.590 | -0.5323 | Yes |
| 10 | HLA-DPA1 |  |  | 18376 | -82.260 | -0.4927 | Yes |
| 11 | HLA-DMA |  |  | 18388 | -85.320 | -0.4497 | Yes |
| 12 | HLA-DRB1 |  |  | 18392 | -86.860 | -0.4064 | Yes |
| 13 | HLA-DOA |  |  | 18426 | -97.930 | -0.3646 | Yes |
| 14 | HLA-DMB |  |  | 18587 | -159.700 | -0.3292 | Yes |
| 15 | HLA-DRA |  |  | 18720 | -232.200 | -0.2924 | Yes |
| 16 | IL10 |  |  | 18725 | -237.700 | -0.2491 | Yes |
| 17 | HLA-DRB5 |  |  | 18733 | -243.500 | -0.2060 | Yes |
| 18 | HLA-DOB |  |  | 18766 | -268.900 | -0.1641 | Yes |
| 19 | CCL11 |  |  | 18783 | -284.800 | -0.1214 | Yes |
| 20 | HLA-DQA1 |  |  | 18983 | -673.000 | -0.0880 | Yes |
| 21 | HLA-DQB1 |  |  | 19125 | -1584.000 | -0.0517 | Yes |
| 22 | MS4A2 |  |  | 19259 | -3784.000 | -0.0149 | Yes |
| 23 | CD40LG |  |  | 19510 | -66050.000 | 0.0160 | Yes |
Table: GSEA details [plain text format]

  

Fig 2: KEGG\_ASTHMA: Random ES distribution      
 Gene set null distribution of ES for **KEGG\_ASTHMA**

  
