# Supplementary material for: DNA methylation-based classifier and gene expression signatures detect BRCAness in osteosarcoma
Source: PLoS Comput Biol. 2021 Nov 11;17(11):e1009562. doi: 10.1371/journal.pcbi.1009562 (PMC8584788; doi:10.1371/journal.pcbi.1009562)
Supplement: S2 File — (ZIP) [file pcbi.1009562.s002.zip › S2_File/my_analysis_Kegg.GseaPreranked.1581692187239/KEGG_BASAL_TRANSCRIPTION_FACTORS.html]

Details for gene set KEGG\_BASAL\_TRANSCRIPTION\_FACTORS[GSEA]

|  || Dataset | DEG3\_two3dTopBottom |
| Phenotype | NoPhenotypeAvailable |
| Upregulated in class | na\_pos |
| GeneSet | KEGG\_BASAL\_TRANSCRIPTION\_FACTORS |
| Enrichment Score (ES) | 0.3275701 |
| Normalized Enrichment Score (NES) | 0.3275701 |
| Nominal p-value | 6.5832783E-4 |
| FDR q-value | 0.02202664 |
| FWER p-Value | 0.342 |
Table: GSEA Results Summary

  

Fig 1: Enrichment plot: KEGG\_BASAL\_TRANSCRIPTION\_FACTORS      
 Profile of the Running ES Score & Positions of GeneSet Members on the Rank Ordered List

  

| PROBE | GENE SYMBOL | GENE\_TITLE | RANK IN GENE LIST | RANK METRIC SCORE | RUNNING ES | CORE ENRICHMENT || 1 | TAF1 |  |  | 256 | 757.600 | 0.0183 | Yes |
| 2 | STON1 |  |  | 749 | 93.560 | 0.0247 | Yes |
| 3 | TAF13 |  |  | 1117 | 44.960 | 0.0374 | Yes |
| 4 | GTF2IRD1 |  |  | 1930 | 18.510 | 0.0277 | Yes |
| 5 | TAF9B |  |  | 2368 | 13.620 | 0.0368 | Yes |
| 6 | TAF4B |  |  | 2378 | 13.540 | 0.0676 | Yes |
| 7 | TAF11 |  |  | 2568 | 11.930 | 0.0893 | Yes |
| 8 | TAF12 |  |  | 3316 | 7.971 | 0.0828 | Yes |
| 9 | GTF2I |  |  | 3344 | 7.848 | 0.1127 | Yes |
| 10 | GTF2F1 |  |  | 3936 | 6.086 | 0.1141 | Yes |
| 11 | TAF7 |  |  | 4031 | 5.899 | 0.1406 | Yes |
| 12 | GTF2H3 |  |  | 4447 | 5.072 | 0.1509 | Yes |
| 13 | TAF4 |  |  | 4493 | 4.992 | 0.1799 | Yes |
| 14 | TAF6L |  |  | 4653 | 4.721 | 0.2031 | Yes |
| 15 | TAF10 |  |  | 4840 | 4.415 | 0.2250 | Yes |
| 16 | GTF2B |  |  | 4875 | 4.353 | 0.2545 | Yes |
| 17 | TAF1L |  |  | 4995 | 4.205 | 0.2797 | Yes |
| 18 | GTF2H4 |  |  | 5247 | 3.878 | 0.2983 | Yes |
| 19 | TAF6 |  |  | 5711 | 3.373 | 0.3062 | Yes |
| 20 | GTF2H2 |  |  | 7018 | 2.398 | 0.2714 | Yes |
| 21 | GTF2A2 |  |  | 7325 | 2.231 | 0.2872 | Yes |
| 22 | TAF9 |  |  | 7553 | 2.122 | 0.3070 | Yes |
| 23 | GTF2H1 |  |  | 8216 | 1.825 | 0.3048 | Yes |
| 24 | TAF5L |  |  | 8385 | 1.763 | 0.3276 | Yes |
| 25 | TBP |  |  | 9177 | 1.503 | 0.3189 | No |
| 26 | GTF2A1 |  |  | 9750 | 1.348 | 0.3212 | No |
| 27 | TAF7L |  |  | 10554 | 1.177 | 0.3119 | No |
| 28 | GTF2F2 |  |  | 11835 | -1.040 | 0.2785 | No |
| 29 | GTF2E1 |  |  | 11939 | -1.057 | 0.3045 | No |
| 30 | TBPL1 |  |  | 12115 | -1.092 | 0.3269 | No |
| 31 | TAF5 |  |  | 12916 | -1.263 | 0.3178 | No |
| 32 | GTF2E2 |  |  | 14319 | -1.879 | 0.2782 | No |
Table: GSEA details [plain text format]

  

Fig 2: KEGG\_BASAL\_TRANSCRIPTION\_FACTORS: Random ES distribution      
 Gene set null distribution of ES for **KEGG\_BASAL\_TRANSCRIPTION\_FACTORS**

  
