# Supplementary material for: DNA methylation-based classifier and gene expression signatures detect BRCAness in osteosarcoma
Source: PLoS Comput Biol. 2021 Nov 11;17(11):e1009562. doi: 10.1371/journal.pcbi.1009562 (PMC8584788; doi:10.1371/journal.pcbi.1009562)
Supplement: S2 File — (ZIP) [file pcbi.1009562.s002.zip › S2_File/my_analysis_Kegg.GseaPreranked.1581692187239/KEGG_BASE_EXCISION_REPAIR.html]

Details for gene set KEGG\_BASE\_EXCISION\_REPAIR[GSEA]

|  || Dataset | DEG3\_two3dTopBottom |
| Phenotype | NoPhenotypeAvailable |
| Upregulated in class | na\_pos |
| GeneSet | KEGG\_BASE\_EXCISION\_REPAIR |
| Enrichment Score (ES) | 0.33920026 |
| Normalized Enrichment Score (NES) | 0.33920026 |
| Nominal p-value | 6.644518E-4 |
| FDR q-value | 0.018919686 |
| FWER p-Value | 0.262 |
Table: GSEA Results Summary

  

Fig 1: Enrichment plot: KEGG\_BASE\_EXCISION\_REPAIR      
 Profile of the Running ES Score & Positions of GeneSet Members on the Rank Ordered List

  

| PROBE | GENE SYMBOL | GENE\_TITLE | RANK IN GENE LIST | RANK METRIC SCORE | RUNNING ES | CORE ENRICHMENT || 1 | SMUG1 |  |  | 61 | 14230.000 | 0.0282 | Yes |
| 2 | LIG3 |  |  | 85 | 6969.000 | 0.0583 | Yes |
| 3 | POLD2 |  |  | 267 | 687.000 | 0.0804 | Yes |
| 4 | APEX2 |  |  | 773 | 86.380 | 0.0861 | Yes |
| 5 | POLE2 |  |  | 914 | 62.780 | 0.1103 | Yes |
| 6 | FEN1 |  |  | 1643 | 23.820 | 0.1048 | Yes |
| 7 | XRCC1 |  |  | 1898 | 19.000 | 0.1232 | Yes |
| 8 | APEX1 |  |  | 2070 | 16.830 | 0.1458 | Yes |
| 9 | POLD1 |  |  | 2192 | 15.340 | 0.1709 | Yes |
| 10 | POLE |  |  | 2249 | 14.810 | 0.1993 | Yes |
| 11 | LIG1 |  |  | 2303 | 14.320 | 0.2279 | Yes |
| 12 | PARP2 |  |  | 2345 | 13.830 | 0.2571 | Yes |
| 13 | POLD3 |  |  | 3045 | 9.243 | 0.2530 | Yes |
| 14 | NTHL1 |  |  | 3515 | 7.295 | 0.2606 | Yes |
| 15 | POLE4 |  |  | 3842 | 6.309 | 0.2754 | Yes |
| 16 | NEIL3 |  |  | 4785 | 4.507 | 0.2590 | Yes |
| 17 | POLE3 |  |  | 4864 | 4.371 | 0.2863 | Yes |
| 18 | TDG |  |  | 5123 | 4.054 | 0.3046 | Yes |
| 19 | UNG |  |  | 5601 | 3.471 | 0.3117 | Yes |
| 20 | PARP1 |  |  | 6229 | 2.915 | 0.3113 | Yes |
| 21 | HMGB1 |  |  | 6296 | 2.861 | 0.3392 | Yes |
| 22 | POLL |  |  | 8688 | 1.656 | 0.2497 | No |
| 23 | OGG1 |  |  | 9276 | 1.470 | 0.2513 | No |
| 24 | MPG |  |  | 9489 | 1.416 | 0.2718 | No |
| 25 | NEIL2 |  |  | 9726 | 1.353 | 0.2911 | No |
| 26 | MBD4 |  |  | 11085 | 1.086 | 0.2538 | No |
| 27 | POLB |  |  | 11544 | 1.006 | 0.2619 | No |
| 28 | MUTYH |  |  | 12133 | -1.094 | 0.2634 | No |
| 29 | POLD4 |  |  | 12325 | -1.133 | 0.2850 | No |
| 30 | PARP3 |  |  | 13391 | -1.412 | 0.2625 | No |
| 31 | NEIL1 |  |  | 15200 | -2.678 | 0.2024 | No |
| 32 | PARP4 |  |  | 15643 | -3.370 | 0.2113 | No |
Table: GSEA details [plain text format]

  

Fig 2: KEGG\_BASE\_EXCISION\_REPAIR: Random ES distribution      
 Gene set null distribution of ES for **KEGG\_BASE\_EXCISION\_REPAIR**

  
