# Supplementary material for: DNA methylation-based classifier and gene expression signatures detect BRCAness in osteosarcoma
Source: PLoS Comput Biol. 2021 Nov 11;17(11):e1009562. doi: 10.1371/journal.pcbi.1009562 (PMC8584788; doi:10.1371/journal.pcbi.1009562)
Supplement: S2 File — (ZIP) [file pcbi.1009562.s002.zip › S2_File/my_analysis_Kegg.GseaPreranked.1581692187239/KEGG_CELL_ADHESION_MOLECULES_CAMS.html]

Details for gene set KEGG\_CELL\_ADHESION\_MOLECULES\_CAMS[GSEA]

|  || Dataset | DEG3\_two3dTopBottom |
| Phenotype | NoPhenotypeAvailable |
| Upregulated in class | na\_neg |
| GeneSet | KEGG\_CELL\_ADHESION\_MOLECULES\_CAMS |
| Enrichment Score (ES) | -0.4682564 |
| Normalized Enrichment Score (NES) | -0.4682564 |
| Nominal p-value | 0.0 |
| FDR q-value | 6.902561E-4 |
| FWER p-Value | 0.0053333333 |
Table: GSEA Results Summary

  

Fig 1: Enrichment plot: KEGG\_CELL\_ADHESION\_MOLECULES\_CAMS      
 Profile of the Running ES Score & Positions of GeneSet Members on the Rank Ordered List

  

| PROBE | GENE SYMBOL | GENE\_TITLE | RANK IN GENE LIST | RANK METRIC SCORE | RUNNING ES | CORE ENRICHMENT || 1 | NRXN2 |  |  | 593 | 145.200 | -0.0219 | No |
| 2 | GLG1 |  |  | 671 | 112.700 | -0.0176 | No |
| 3 | CDH2 |  |  | 1328 | 34.400 | -0.0427 | No |
| 4 | SDC2 |  |  | 1736 | 22.000 | -0.0552 | No |
| 5 | NCAM1 |  |  | 1920 | 18.620 | -0.0563 | No |
| 6 | CDH15 |  |  | 2521 | 12.390 | -0.0785 | No |
| 7 | NCAM2 |  |  | 4770 | 4.527 | -0.1844 | No |
| 8 | NEGR1 |  |  | 6063 | 3.040 | -0.2418 | No |
| 9 | CADM1 |  |  | 6419 | 2.763 | -0.2516 | No |
| 10 | NLGN3 |  |  | 6732 | 2.575 | -0.2592 | No |
| 11 | NRXN1 |  |  | 6737 | 2.573 | -0.2512 | No |
| 12 | ITGB8 |  |  | 6817 | 2.527 | -0.2470 | No |
| 13 | CLDN20 |  |  | 7147 | 2.316 | -0.2555 | No |
| 14 | CLDN19 |  |  | 7216 | 2.283 | -0.2508 | No |
| 15 | PVR |  |  | 7439 | 2.180 | -0.2539 | No |
| 16 | VCAN |  |  | 7782 | 2.023 | -0.2630 | No |
| 17 | CD276 |  |  | 8983 | 1.557 | -0.3157 | No |
| 18 | NEO1 |  |  | 9097 | 1.523 | -0.3133 | No |
| 19 | ITGA4 |  |  | 9100 | 1.522 | -0.3052 | No |
| 20 | NLGN4X |  |  | 9171 | 1.505 | -0.3005 | No |
| 21 | ICOSLG |  |  | 9649 | 1.374 | -0.3165 | No |
| 22 | CLDN15 |  |  | 9993 | 1.291 | -0.3257 | No |
| 23 | CD99 |  |  | 10170 | 1.252 | -0.3265 | No |
| 24 | HLA-C |  |  | 10192 | 1.248 | -0.3193 | No |
| 25 | ITGB1 |  |  | 10236 | 1.237 | -0.3133 | No |
| 26 | L1CAM |  |  | 10317 | 1.220 | -0.3092 | No |
| 27 | NLGN2 |  |  | 10626 | 1.166 | -0.3166 | No |
| 28 | MPZL1 |  |  | 11252 | 1.055 | -0.3401 | No |
| 29 | ITGA6 |  |  | 11264 | 1.053 | -0.3325 | No |
| 30 | JAM3 |  |  | 13070 | -1.311 | -0.4159 | No |
| 31 | HLA-A |  |  | 13593 | -1.494 | -0.4342 | No |
| 32 | MPZ |  |  | 14265 | -1.846 | -0.4601 | Yes |
| 33 | CNTN2 |  |  | 14317 | -1.878 | -0.4545 | Yes |
| 34 | MADCAM1 |  |  | 14340 | -1.893 | -0.4474 | Yes |
| 35 | CD86 |  |  | 14450 | -1.967 | -0.4447 | Yes |
| 36 | SDC3 |  |  | 14615 | -2.081 | -0.4448 | Yes |
| 37 | CD58 |  |  | 14665 | -2.123 | -0.4391 | Yes |
| 38 | NLGN1 |  |  | 14678 | -2.131 | -0.4315 | Yes |
| 39 | ITGB7 |  |  | 14706 | -2.153 | -0.4247 | Yes |
| 40 | NRXN3 |  |  | 14853 | -2.266 | -0.4239 | Yes |
| 41 | HLA-B |  |  | 14983 | -2.404 | -0.4223 | Yes |
| 42 | JAM2 |  |  | 15072 | -2.521 | -0.4185 | Yes |
| 43 | MAG |  |  | 15469 | -3.063 | -0.4304 | Yes |
| 44 | PTPRF |  |  | 15580 | -3.246 | -0.4278 | Yes |
| 45 | ICAM2 |  |  | 15583 | -3.249 | -0.4197 | Yes |
| 46 | CADM3 |  |  | 15599 | -3.266 | -0.4123 | Yes |
| 47 | CD34 |  |  | 15806 | -3.664 | -0.4146 | Yes |
| 48 | CLDN1 |  |  | 15882 | -3.797 | -0.4102 | Yes |
| 49 | CLDN14 |  |  | 15909 | -3.860 | -0.4033 | Yes |
| 50 | HLA-F |  |  | 16065 | -4.220 | -0.4030 | Yes |
| 51 | ALCAM |  |  | 16181 | -4.612 | -0.4006 | Yes |
| 52 | ESAM |  |  | 16508 | -5.771 | -0.4089 | Yes |
| 53 | CD28 |  |  | 16514 | -5.798 | -0.4010 | Yes |
| 54 | ITGB2 |  |  | 16563 | -5.997 | -0.3952 | Yes |
| 55 | CNTN1 |  |  | 16697 | -6.691 | -0.3938 | Yes |
| 56 | HLA-G |  |  | 16786 | -7.343 | -0.3901 | Yes |
| 57 | PTPRM |  |  | 16844 | -7.787 | -0.3848 | Yes |
| 58 | SIGLEC1 |  |  | 16883 | -8.103 | -0.3785 | Yes |
| 59 | VCAM1 |  |  | 16932 | -8.548 | -0.3727 | Yes |
| 60 | ITGAV |  |  | 16933 | -8.552 | -0.3645 | Yes |
| 61 | SDC4 |  |  | 16937 | -8.600 | -0.3565 | Yes |
| 62 | CLDN11 |  |  | 16965 | -8.814 | -0.3497 | Yes |
| 63 | CD40 |  |  | 17248 | -11.890 | -0.3558 | Yes |
| 64 | CLDN16 |  |  | 17370 | -14.000 | -0.3537 | Yes |
| 65 | ITGA9 |  |  | 17389 | -14.300 | -0.3464 | Yes |
| 66 | ITGAM |  |  | 17547 | -17.420 | -0.3462 | Yes |
| 67 | CLDN6 |  |  | 17696 | -22.040 | -0.3455 | Yes |
| 68 | SELP |  |  | 17753 | -24.600 | -0.3402 | Yes |
| 69 | NRCAM |  |  | 17791 | -26.080 | -0.3338 | Yes |
| 70 | CLDN3 |  |  | 17896 | -31.040 | -0.3309 | Yes |
| 71 | HLA-DQA2 |  |  | 17900 | -31.160 | -0.3229 | Yes |
| 72 | ITGA8 |  |  | 17960 | -34.640 | -0.3177 | Yes |
| 73 | CLDN5 |  |  | 18050 | -41.260 | -0.3140 | Yes |
| 74 | ICAM3 |  |  | 18073 | -42.630 | -0.3069 | Yes |
| 75 | CDH3 |  |  | 18133 | -48.270 | -0.3017 | Yes |
| 76 | SELL |  |  | 18235 | -59.550 | -0.2986 | Yes |
| 77 | HLA-DPB1 |  |  | 18263 | -63.760 | -0.2918 | Yes |
| 78 | CDH4 |  |  | 18347 | -76.630 | -0.2878 | Yes |
| 79 | HLA-DPA1 |  |  | 18376 | -82.260 | -0.2811 | Yes |
| 80 | HLA-DMA |  |  | 18388 | -85.320 | -0.2734 | Yes |
| 81 | HLA-DRB1 |  |  | 18392 | -86.860 | -0.2654 | Yes |
| 82 | CDH5 |  |  | 18416 | -94.490 | -0.2583 | Yes |
| 83 | HLA-DOA |  |  | 18426 | -97.930 | -0.2506 | Yes |
| 84 | SDC1 |  |  | 18499 | -120.800 | -0.2461 | Yes |
| 85 | CLDN10 |  |  | 18502 | -122.100 | -0.2380 | Yes |
| 86 | HLA-DMB |  |  | 18587 | -159.700 | -0.2340 | Yes |
| 87 | OCLN |  |  | 18597 | -163.600 | -0.2263 | Yes |
| 88 | HLA-E |  |  | 18671 | -203.600 | -0.2218 | Yes |
| 89 | HLA-DRA |  |  | 18720 | -232.200 | -0.2160 | Yes |
| 90 | HLA-DRB5 |  |  | 18733 | -243.500 | -0.2085 | Yes |
| 91 | PECAM1 |  |  | 18745 | -250.000 | -0.2008 | Yes |
| 92 | HLA-DOB |  |  | 18766 | -268.900 | -0.1936 | Yes |
| 93 | CD4 |  |  | 18780 | -283.000 | -0.1861 | Yes |
| 94 | CD8B |  |  | 18793 | -289.900 | -0.1785 | Yes |
| 95 | CTLA4 |  |  | 18796 | -291.400 | -0.1704 | Yes |
| 96 | CD80 |  |  | 18879 | -411.400 | -0.1664 | Yes |
| 97 | CLDN9 |  |  | 18929 | -522.200 | -0.1607 | Yes |
| 98 | HLA-DQA1 |  |  | 18983 | -673.000 | -0.1552 | Yes |
| 99 | NFASC |  |  | 19025 | -839.700 | -0.1490 | Yes |
| 100 | CLDN18 |  |  | 19082 | -1198.000 | -0.1437 | Yes |
| 101 | HLA-DQB1 |  |  | 19125 | -1584.000 | -0.1376 | Yes |
| 102 | CLDN2 |  |  | 19133 | -1645.000 | -0.1298 | Yes |
| 103 | PDCD1 |  |  | 19134 | -1645.000 | -0.1216 | Yes |
| 104 | SELPLG |  |  | 19171 | -2043.000 | -0.1152 | Yes |
| 105 | CD22 |  |  | 19215 | -2706.000 | -0.1092 | Yes |
| 106 | ICOS |  |  | 19287 | -4615.000 | -0.1046 | Yes |
| 107 | CLDN23 |  |  | 19336 | -7196.000 | -0.0988 | Yes |
| 108 | CDH1 |  |  | 19353 | -8645.000 | -0.0915 | Yes |
| 109 | CLDN4 |  |  | 19375 | -10540.000 | -0.0843 | Yes |
| 110 | CD226 |  |  | 19458 | -28510.000 | -0.0803 | Yes |
| 111 | PTPRC |  |  | 19477 | -39510.000 | -0.0730 | Yes |
| 112 | PDCD1LG2 |  |  | 19491 | -49830.000 | -0.0655 | Yes |
| 113 | CD40LG |  |  | 19510 | -66050.000 | -0.0582 | Yes |
| 114 | CD8A |  |  | 19544 | -106100.000 | -0.0517 | Yes |
| 115 | CD2 |  |  | 19585 | -188800.000 | -0.0455 | Yes |
| 116 | ITGAL |  |  | 19648 | -514800.000 | -0.0405 | Yes |
| 117 | SPN |  |  | 19732 | -7959000.000 | -0.0365 | Yes |
| 118 | F11R |  |  | 19743 | -10770000.000 | -0.0288 | Yes |
| 119 | ICAM1 |  |  | 19781 | -101300000.000 | -0.0225 | Yes |
| 120 | CLDN7 |  |  | 19790 | -204100000.000 | -0.0147 | Yes |
| 121 | CD6 |  |  | 19791 | -224600000.000 | -0.0065 | Yes |
| 122 | CD274 |  |  | 19817 | -202099998720.000 | 0.0005 | Yes |
Table: GSEA details [plain text format]

  

Fig 2: KEGG\_CELL\_ADHESION\_MOLECULES\_CAMS: Random ES distribution      
 Gene set null distribution of ES for **KEGG\_CELL\_ADHESION\_MOLECULES\_CAMS**

  
