# Supplementary material for: DNA methylation-based classifier and gene expression signatures detect BRCAness in osteosarcoma
Source: PLoS Comput Biol. 2021 Nov 11;17(11):e1009562. doi: 10.1371/journal.pcbi.1009562 (PMC8584788; doi:10.1371/journal.pcbi.1009562)
Supplement: S2 File — (ZIP) [file pcbi.1009562.s002.zip › S2_File/my_analysis_Kegg.GseaPreranked.1581692187239/KEGG_CELL_CYCLE.html]

Details for gene set KEGG\_CELL\_CYCLE[GSEA]

|  || Dataset | DEG3\_two3dTopBottom |
| Phenotype | NoPhenotypeAvailable |
| Upregulated in class | na\_pos |
| GeneSet | KEGG\_CELL\_CYCLE |
| Enrichment Score (ES) | 0.290146 |
| Normalized Enrichment Score (NES) | 0.290146 |
| Nominal p-value | 0.0 |
| FDR q-value | 0.04513919 |
| FWER p-Value | 0.6523333 |
Table: GSEA Results Summary

  

Fig 1: Enrichment plot: KEGG\_CELL\_CYCLE      
 Profile of the Running ES Score & Positions of GeneSet Members on the Rank Ordered List

  

| PROBE | GENE SYMBOL | GENE\_TITLE | RANK IN GENE LIST | RANK METRIC SCORE | RUNNING ES | CORE ENRICHMENT || 1 | MAD1L1 |  |  | 121 | 3385.000 | 0.0030 | Yes |
| 2 | CDC6 |  |  | 358 | 378.000 | 0.0001 | Yes |
| 3 | SKP2 |  |  | 368 | 362.700 | 0.0087 | Yes |
| 4 | PKMYT1 |  |  | 401 | 298.700 | 0.0162 | Yes |
| 5 | SMC1B |  |  | 585 | 148.200 | 0.0160 | Yes |
| 6 | CDC25C |  |  | 606 | 141.800 | 0.0241 | Yes |
| 7 | CDC7 |  |  | 611 | 140.700 | 0.0330 | Yes |
| 8 | ESPL1 |  |  | 753 | 91.720 | 0.0349 | Yes |
| 9 | TGFB1 |  |  | 760 | 88.870 | 0.0437 | Yes |
| 10 | BUB1 |  |  | 769 | 87.210 | 0.0524 | Yes |
| 11 | PLK1 |  |  | 783 | 83.980 | 0.0608 | Yes |
| 12 | CCNE1 |  |  | 1018 | 51.770 | 0.0580 | Yes |
| 13 | CDC27 |  |  | 1201 | 40.610 | 0.0579 | Yes |
| 14 | CDK2 |  |  | 1332 | 34.210 | 0.0604 | Yes |
| 15 | CDC45 |  |  | 1369 | 32.670 | 0.0676 | Yes |
| 16 | E2F1 |  |  | 1414 | 30.710 | 0.0745 | Yes |
| 17 | CHEK1 |  |  | 1430 | 30.040 | 0.0828 | Yes |
| 18 | MCM3 |  |  | 1802 | 20.500 | 0.0731 | Yes |
| 19 | CCNB1 |  |  | 1880 | 19.130 | 0.0783 | Yes |
| 20 | RBL1 |  |  | 1994 | 17.790 | 0.0817 | Yes |
| 21 | CCNB2 |  |  | 2042 | 17.190 | 0.0884 | Yes |
| 22 | MAD2L1 |  |  | 2068 | 16.840 | 0.0962 | Yes |
| 23 | TTK |  |  | 2093 | 16.510 | 0.1041 | Yes |
| 24 | ORC1 |  |  | 2188 | 15.360 | 0.1084 | Yes |
| 25 | MCM2 |  |  | 2194 | 15.310 | 0.1172 | Yes |
| 26 | YWHAQ |  |  | 2531 | 12.300 | 0.1093 | Yes |
| 27 | MCM6 |  |  | 2797 | 10.490 | 0.1049 | Yes |
| 28 | SMC1A |  |  | 2894 | 10.030 | 0.1091 | Yes |
| 29 | PTTG1 |  |  | 2998 | 9.494 | 0.1130 | Yes |
| 30 | MAD2L2 |  |  | 3003 | 9.469 | 0.1219 | Yes |
| 31 | PRKDC |  |  | 3102 | 8.948 | 0.1260 | Yes |
| 32 | CDC23 |  |  | 3262 | 8.150 | 0.1270 | Yes |
| 33 | E2F2 |  |  | 3582 | 7.096 | 0.1200 | Yes |
| 34 | CDKN2C |  |  | 3594 | 7.068 | 0.1285 | Yes |
| 35 | BUB1B |  |  | 3697 | 6.750 | 0.1324 | Yes |
| 36 | CHEK2 |  |  | 3779 | 6.517 | 0.1374 | Yes |
| 37 | CDC20 |  |  | 3882 | 6.218 | 0.1413 | Yes |
| 38 | E2F3 |  |  | 3944 | 6.065 | 0.1473 | Yes |
| 39 | MCM4 |  |  | 3959 | 6.035 | 0.1557 | Yes |
| 40 | DBF4 |  |  | 4046 | 5.867 | 0.1604 | Yes |
| 41 | BUB3 |  |  | 4420 | 5.139 | 0.1506 | Yes |
| 42 | STAG2 |  |  | 4459 | 5.054 | 0.1577 | Yes |
| 43 | MCM7 |  |  | 4544 | 4.903 | 0.1626 | Yes |
| 44 | CDC16 |  |  | 4681 | 4.671 | 0.1648 | Yes |
| 45 | ORC6 |  |  | 4700 | 4.635 | 0.1729 | Yes |
| 46 | ATM |  |  | 4749 | 4.569 | 0.1796 | Yes |
| 47 | CCND3 |  |  | 4819 | 4.443 | 0.1852 | Yes |
| 48 | CDC26 |  |  | 5163 | 3.993 | 0.1769 | Yes |
| 49 | FZR1 |  |  | 5516 | 3.565 | 0.1681 | Yes |
| 50 | CDK1 |  |  | 5639 | 3.435 | 0.1710 | Yes |
| 51 | MCM5 |  |  | 5940 | 3.156 | 0.1649 | Yes |
| 52 | GADD45A |  |  | 5991 | 3.100 | 0.1715 | Yes |
| 53 | CCNE2 |  |  | 6260 | 2.889 | 0.1670 | Yes |
| 54 | ORC4 |  |  | 6384 | 2.796 | 0.1698 | Yes |
| 55 | SMAD4 |  |  | 6671 | 2.614 | 0.1644 | Yes |
| 56 | HDAC2 |  |  | 7042 | 2.384 | 0.1547 | Yes |
| 57 | ORC5 |  |  | 7089 | 2.353 | 0.1615 | Yes |
| 58 | TFDP1 |  |  | 7140 | 2.325 | 0.1680 | Yes |
| 59 | YWHAZ |  |  | 7239 | 2.272 | 0.1722 | Yes |
| 60 | MYC |  |  | 7624 | 2.092 | 0.1618 | Yes |
| 61 | SMAD2 |  |  | 7757 | 2.032 | 0.1642 | Yes |
| 62 | CCNH |  |  | 7810 | 2.012 | 0.1706 | Yes |
| 63 | CREBBP |  |  | 8098 | 1.877 | 0.1652 | Yes |
| 64 | GADD45B |  |  | 8283 | 1.799 | 0.1649 | Yes |
| 65 | ZBTB17 |  |  | 8339 | 1.781 | 0.1712 | Yes |
| 66 | ORC2 |  |  | 8363 | 1.772 | 0.1791 | Yes |
| 67 | YWHAE |  |  | 8568 | 1.701 | 0.1779 | Yes |
| 68 | ATR |  |  | 8691 | 1.653 | 0.1808 | Yes |
| 69 | CDKN2A |  |  | 8708 | 1.648 | 0.1891 | Yes |
| 70 | E2F4 |  |  | 8713 | 1.646 | 0.1980 | Yes |
| 71 | RBX1 |  |  | 8857 | 1.602 | 0.1998 | Yes |
| 72 | E2F5 |  |  | 8887 | 1.591 | 0.2074 | Yes |
| 73 | RB1 |  |  | 9191 | 1.498 | 0.2011 | Yes |
| 74 | TGFB3 |  |  | 9199 | 1.494 | 0.2099 | Yes |
| 75 | TFDP2 |  |  | 9642 | 1.376 | 0.1966 | Yes |
| 76 | CDKN1B |  |  | 9723 | 1.353 | 0.2016 | Yes |
| 77 | ORC3 |  |  | 9770 | 1.344 | 0.2083 | Yes |
| 78 | SMC3 |  |  | 9868 | 1.322 | 0.2125 | Yes |
| 79 | CDC25A |  |  | 9982 | 1.294 | 0.2159 | Yes |
| 80 | HDAC1 |  |  | 10142 | 1.258 | 0.2169 | Yes |
| 81 | CDC25B |  |  | 10206 | 1.244 | 0.2228 | Yes |
| 82 | GSK3B |  |  | 10378 | 1.207 | 0.2232 | Yes |
| 83 | WEE1 |  |  | 10503 | 1.185 | 0.2260 | Yes |
| 84 | ABL1 |  |  | 10517 | 1.182 | 0.2344 | Yes |
| 85 | YWHAH |  |  | 10717 | 1.151 | 0.2334 | Yes |
| 86 | EP300 |  |  | 10745 | 1.145 | 0.2412 | Yes |
| 87 | STAG1 |  |  | 10973 | 1.105 | 0.2387 | Yes |
| 88 | GADD45G |  |  | 11016 | 1.097 | 0.2457 | Yes |
| 89 | PTTG2 |  |  | 11029 | 1.096 | 0.2542 | Yes |
| 90 | CDK6 |  |  | 11140 | 1.075 | 0.2577 | Yes |
| 91 | CCNB3 |  |  | 11285 | 1.048 | 0.2595 | Yes |
| 92 | CDKN2B |  |  | 11325 | 1.041 | 0.2666 | Yes |
| 93 | YWHAB |  |  | 11509 | 1.010 | 0.2664 | Yes |
| 94 | WEE2 |  |  | 11554 | 1.004 | 0.2733 | Yes |
| 95 | CDK7 |  |  | 11645 | -1.011 | 0.2778 | Yes |
| 96 | CUL1 |  |  | 11729 | -1.024 | 0.2827 | Yes |
| 97 | CDC14A |  |  | 11762 | -1.030 | 0.2901 | Yes |
| 98 | CDKN1C |  |  | 13296 | -1.381 | 0.2215 | No |
| 99 | TGFB2 |  |  | 13716 | -1.553 | 0.2093 | No |
| 100 | SKP1 |  |  | 13849 | -1.616 | 0.2117 | No |
| 101 | TP53 |  |  | 13922 | -1.649 | 0.2172 | No |
| 102 | CDC14B |  |  | 14106 | -1.747 | 0.2170 | No |
| 103 | RBL2 |  |  | 14485 | -1.994 | 0.2069 | No |
| 104 | YWHAG |  |  | 14520 | -2.016 | 0.2143 | No |
| 105 | SMAD3 |  |  | 14593 | -2.066 | 0.2197 | No |
| 106 | CCND1 |  |  | 15967 | -3.992 | 0.1592 | No |
| 107 | CDKN1A |  |  | 16216 | -4.730 | 0.1557 | No |
| 108 | CDK4 |  |  | 18807 | -305.100 | 0.0334 | No |
| 109 | CCND2 |  |  | 19511 | -66060.000 | 0.0068 | No |
| 110 | SFN |  |  | 19593 | -200800.000 | 0.0118 | No |
Table: GSEA details [plain text format]

  

Fig 2: KEGG\_CELL\_CYCLE: Random ES distribution      
 Gene set null distribution of ES for **KEGG\_CELL\_CYCLE**

  
