# Supplementary material for: DNA methylation-based classifier and gene expression signatures detect BRCAness in osteosarcoma
Source: PLoS Comput Biol. 2021 Nov 11;17(11):e1009562. doi: 10.1371/journal.pcbi.1009562 (PMC8584788; doi:10.1371/journal.pcbi.1009562)
Supplement: S2 File — (ZIP) [file pcbi.1009562.s002.zip › S2_File/my_analysis_Kegg.GseaPreranked.1581692187239/KEGG_COMPLEMENT_AND_COAGULATION_CASCADES.html]

Details for gene set KEGG\_COMPLEMENT\_AND\_COAGULATION\_CASCADES[GSEA]

|  || Dataset | DEG3\_two3dTopBottom |
| Phenotype | NoPhenotypeAvailable |
| Upregulated in class | na\_neg |
| GeneSet | KEGG\_COMPLEMENT\_AND\_COAGULATION\_CASCADES |
| Enrichment Score (ES) | -0.48676568 |
| Normalized Enrichment Score (NES) | -0.48676568 |
| Nominal p-value | 0.0 |
| FDR q-value | 5.324247E-4 |
| FWER p-Value | 0.0036666666 |
Table: GSEA Results Summary

  

Fig 1: Enrichment plot: KEGG\_COMPLEMENT\_AND\_COAGULATION\_CASCADES      
 Profile of the Running ES Score & Positions of GeneSet Members on the Rank Ordered List

  

| PROBE | GENE SYMBOL | GENE\_TITLE | RANK IN GENE LIST | RANK METRIC SCORE | RUNNING ES | CORE ENRICHMENT || 1 | F2 |  |  | 1041 | 50.070 | -0.0363 | No |
| 2 | MBL2 |  |  | 1540 | 26.720 | -0.0451 | No |
| 3 | KNG1 |  |  | 1809 | 20.350 | -0.0422 | No |
| 4 | SERPINC1 |  |  | 5046 | 4.144 | -0.1896 | No |
| 5 | F7 |  |  | 6960 | 2.437 | -0.2700 | No |
| 6 | C9 |  |  | 7053 | 2.376 | -0.2582 | No |
| 7 | F3 |  |  | 8748 | 1.631 | -0.3275 | No |
| 8 | PROC |  |  | 9239 | 1.482 | -0.3359 | No |
| 9 | C5 |  |  | 10819 | 1.132 | -0.3994 | No |
| 10 | SERPIND1 |  |  | 11303 | 1.045 | -0.4075 | No |
| 11 | F13A1 |  |  | 11748 | -1.027 | -0.4135 | No |
| 12 | PLAUR |  |  | 11787 | -1.033 | -0.3990 | No |
| 13 | C8G |  |  | 11967 | -1.062 | -0.3917 | No |
| 14 | MASP2 |  |  | 12747 | -1.221 | -0.4147 | No |
| 15 | A2M |  |  | 13179 | -1.346 | -0.4201 | No |
| 16 | F2R |  |  | 13572 | -1.488 | -0.4236 | No |
| 17 | CFH |  |  | 13869 | -1.624 | -0.4222 | No |
| 18 | BDKRB1 |  |  | 14020 | -1.700 | -0.4134 | No |
| 19 | F12 |  |  | 15472 | -3.066 | -0.4704 | Yes |
| 20 | THBD |  |  | 15628 | -3.327 | -0.4618 | Yes |
| 21 | C1QC |  |  | 15638 | -3.354 | -0.4459 | Yes |
| 22 | CFD |  |  | 15828 | -3.696 | -0.4391 | Yes |
| 23 | KLKB1 |  |  | 15894 | -3.830 | -0.4259 | Yes |
| 24 | PLAU |  |  | 16036 | -4.161 | -0.4167 | Yes |
| 25 | C1R |  |  | 16350 | -5.204 | -0.4161 | Yes |
| 26 | SERPINF2 |  |  | 16372 | -5.274 | -0.4008 | Yes |
| 27 | PROS1 |  |  | 16487 | -5.683 | -0.3902 | Yes |
| 28 | BDKRB2 |  |  | 16554 | -5.972 | -0.3771 | Yes |
| 29 | C1QA |  |  | 16628 | -6.295 | -0.3644 | Yes |
| 30 | CFI |  |  | 16644 | -6.406 | -0.3488 | Yes |
| 31 | F8 |  |  | 16651 | -6.428 | -0.3327 | Yes |
| 32 | SERPINE1 |  |  | 16717 | -6.832 | -0.3196 | Yes |
| 33 | C6 |  |  | 16793 | -7.395 | -0.3070 | Yes |
| 34 | CD59 |  |  | 16867 | -7.978 | -0.2943 | Yes |
| 35 | C1QB |  |  | 16991 | -9.021 | -0.2841 | Yes |
| 36 | CD46 |  |  | 17160 | -10.840 | -0.2762 | Yes |
| 37 | C3AR1 |  |  | 17192 | -11.100 | -0.2614 | Yes |
| 38 | C1S |  |  | 17293 | -12.770 | -0.2501 | Yes |
| 39 | C4BPA |  |  | 17324 | -13.190 | -0.2352 | Yes |
| 40 | VWF |  |  | 17478 | -15.600 | -0.2265 | Yes |
| 41 | C3 |  |  | 17530 | -16.820 | -0.2127 | Yes |
| 42 | C5AR1 |  |  | 17560 | -17.850 | -0.1978 | Yes |
| 43 | CR1 |  |  | 17742 | -24.130 | -0.1906 | Yes |
| 44 | MASP1 |  |  | 17884 | -30.400 | -0.1813 | Yes |
| 45 | C8B |  |  | 17953 | -34.240 | -0.1683 | Yes |
| 46 | F5 |  |  | 18101 | -44.650 | -0.1594 | Yes |
| 47 | C7 |  |  | 18270 | -64.490 | -0.1515 | Yes |
| 48 | C8A |  |  | 18280 | -65.490 | -0.1356 | Yes |
| 49 | CFB |  |  | 18433 | -100.500 | -0.1269 | Yes |
| 50 | CD55 |  |  | 18544 | -138.300 | -0.1160 | Yes |
| 51 | C2 |  |  | 18556 | -144.700 | -0.1002 | Yes |
| 52 | PLAT |  |  | 18579 | -157.600 | -0.0849 | Yes |
| 53 | F11 |  |  | 18612 | -170.500 | -0.0701 | Yes |
| 54 | SERPING1 |  |  | 18909 | -480.100 | -0.0687 | Yes |
| 55 | TFPI |  |  | 18928 | -514.100 | -0.0532 | Yes |
| 56 | CR2 |  |  | 19147 | -1767.000 | -0.0479 | Yes |
| 57 | F10 |  |  | 19282 | -4456.000 | -0.0383 | Yes |
| 58 | FGG |  |  | 19295 | -4943.000 | -0.0225 | Yes |
| 59 | FGA |  |  | 19428 | -18990.000 | -0.0128 | Yes |
| 60 | C4BPB |  |  | 19519 | -75310.000 | -0.0009 | Yes |
| 61 | CPB2 |  |  | 19823 | -1303999938560.000 | 0.0002 | Yes |
Table: GSEA details [plain text format]

  

Fig 2: KEGG\_COMPLEMENT\_AND\_COAGULATION\_CASCADES: Random ES distribution      
 Gene set null distribution of ES for **KEGG\_COMPLEMENT\_AND\_COAGULATION\_CASCADES**

  
