# Supplementary material for: DNA methylation-based classifier and gene expression signatures detect BRCAness in osteosarcoma
Source: PLoS Comput Biol. 2021 Nov 11;17(11):e1009562. doi: 10.1371/journal.pcbi.1009562 (PMC8584788; doi:10.1371/journal.pcbi.1009562)
Supplement: S2 File — (ZIP) [file pcbi.1009562.s002.zip › S2_File/my_analysis_Kegg.GseaPreranked.1581692187239/KEGG_CYTOKINE_CYTOKINE_RECEPTOR_INTERACTION.html]

Details for gene set KEGG\_CYTOKINE\_CYTOKINE\_RECEPTOR\_INTERACTION[GSEA]

|  || Dataset | DEG3\_two3dTopBottom |
| Phenotype | NoPhenotypeAvailable |
| Upregulated in class | na\_neg |
| GeneSet | KEGG\_CYTOKINE\_CYTOKINE\_RECEPTOR\_INTERACTION |
| Enrichment Score (ES) | -0.39323014 |
| Normalized Enrichment Score (NES) | -0.39323014 |
| Nominal p-value | 0.0 |
| FDR q-value | 0.00603045 |
| FWER p-Value | 0.062333334 |
Table: GSEA Results Summary

  

Fig 1: Enrichment plot: KEGG\_CYTOKINE\_CYTOKINE\_RECEPTOR\_INTERACTION      
 Profile of the Running ES Score & Positions of GeneSet Members on the Rank Ordered List

  

| PROBE | GENE SYMBOL | GENE\_TITLE | RANK IN GENE LIST | RANK METRIC SCORE | RUNNING ES | CORE ENRICHMENT || 1 | INHBB |  |  | 65 | 11970.000 | 0.0011 | No |
| 2 | IL17B |  |  | 80 | 8201.000 | 0.0048 | No |
| 3 | VEGFA |  |  | 86 | 6782.000 | 0.0090 | No |
| 4 | NGFR |  |  | 492 | 212.700 | -0.0072 | No |
| 5 | TGFB1 |  |  | 760 | 88.870 | -0.0164 | No |
| 6 | CCL28 |  |  | 969 | 56.170 | -0.0226 | No |
| 7 | TNFRSF21 |  |  | 1143 | 43.600 | -0.0270 | No |
| 8 | INHBE |  |  | 1350 | 33.290 | -0.0331 | No |
| 9 | INHBC |  |  | 1470 | 28.940 | -0.0348 | No |
| 10 | GHR |  |  | 1513 | 27.470 | -0.0325 | No |
| 11 | VEGFB |  |  | 2277 | 14.530 | -0.0670 | No |
| 12 | TNFRSF13C |  |  | 2609 | 11.680 | -0.0794 | No |
| 13 | PDGFA |  |  | 2697 | 11.030 | -0.0795 | No |
| 14 | IL25 |  |  | 2710 | 10.950 | -0.0756 | No |
| 15 | IL21R |  |  | 2754 | 10.700 | -0.0734 | No |
| 16 | IL22RA1 |  |  | 3496 | 7.338 | -0.1068 | No |
| 17 | CCL26 |  |  | 3834 | 6.329 | -0.1196 | No |
| 18 | TPO |  |  | 4218 | 5.532 | -0.1347 | No |
| 19 | EPO |  |  | 4813 | 4.460 | -0.1606 | No |
| 20 | IL20RB |  |  | 4879 | 4.346 | -0.1595 | No |
| 21 | PF4 |  |  | 5025 | 4.169 | -0.1624 | No |
| 22 | PDGFRA |  |  | 5075 | 4.115 | -0.1605 | No |
| 23 | CD70 |  |  | 5737 | 3.349 | -0.1898 | No |
| 24 | INHBA |  |  | 5875 | 3.206 | -0.1924 | No |
| 25 | FLT1 |  |  | 6816 | 2.527 | -0.2359 | No |
| 26 | CCL25 |  |  | 6865 | 2.497 | -0.2339 | No |
| 27 | TNFSF9 |  |  | 6914 | 2.470 | -0.2319 | No |
| 28 | ACVR2B |  |  | 7090 | 2.351 | -0.2364 | No |
| 29 | CCR10 |  |  | 7368 | 2.210 | -0.2462 | No |
| 30 | AMH |  |  | 8067 | 1.891 | -0.2773 | No |
| 31 | IL10RB |  |  | 8100 | 1.876 | -0.2745 | No |
| 32 | CNTFR |  |  | 8835 | 1.606 | -0.3076 | No |
| 33 | CCL27 |  |  | 8852 | 1.603 | -0.3040 | No |
| 34 | LIFR |  |  | 8858 | 1.601 | -0.2998 | No |
| 35 | FLT3LG |  |  | 8905 | 1.585 | -0.2977 | No |
| 36 | CCL4L2 |  |  | 9065 | 1.533 | -0.3014 | No |
| 37 | CCL3L1 |  |  | 9143 | 1.511 | -0.3009 | No |
| 38 | TGFB3 |  |  | 9199 | 1.494 | -0.2993 | No |
| 39 | TNFRSF12A |  |  | 9388 | 1.441 | -0.3045 | No |
| 40 | IFNW1 |  |  | 9396 | 1.439 | -0.3004 | No |
| 41 | IFNLR1 |  |  | 9734 | 1.351 | -0.3132 | No |
| 42 | ACVR1B |  |  | 9927 | 1.308 | -0.3185 | No |
| 43 | TGFBR1 |  |  | 9980 | 1.295 | -0.3167 | No |
| 44 | CSF1 |  |  | 10023 | 1.284 | -0.3145 | No |
| 45 | IL2RA |  |  | 10115 | 1.263 | -0.3147 | No |
| 46 | EGFR |  |  | 10302 | 1.223 | -0.3197 | No |
| 47 | TNFSF18 |  |  | 10457 | 1.193 | -0.3232 | No |
| 48 | PRL |  |  | 10479 | 1.189 | -0.3198 | No |
| 49 | OSMR |  |  | 10548 | 1.178 | -0.3189 | No |
| 50 | BMPR1B |  |  | 10662 | 1.159 | -0.3202 | No |
| 51 | IL1R2 |  |  | 10747 | 1.145 | -0.3201 | No |
| 52 | BMP2 |  |  | 10855 | 1.126 | -0.3211 | No |
| 53 | EGF |  |  | 10873 | 1.121 | -0.3176 | No |
| 54 | BMP7 |  |  | 10964 | 1.106 | -0.3177 | No |
| 55 | TNFRSF13B |  |  | 10983 | 1.103 | -0.3142 | No |
| 56 | PPBP |  |  | 10987 | 1.102 | -0.3099 | No |
| 57 | TNFRSF1A |  |  | 11195 | 1.067 | -0.3161 | No |
| 58 | IL17RB |  |  | 11413 | 1.025 | -0.3227 | No |
| 59 | PDGFRB |  |  | 11871 | -1.046 | -0.3416 | No |
| 60 | ACVR1 |  |  | 12269 | -1.121 | -0.3574 | No |
| 61 | IFNGR2 |  |  | 12521 | -1.174 | -0.3658 | No |
| 62 | PDGFC |  |  | 12593 | -1.189 | -0.3650 | No |
| 63 | TNFRSF19 |  |  | 12628 | -1.196 | -0.3623 | No |
| 64 | BMPR1A |  |  | 12756 | -1.223 | -0.3644 | No |
| 65 | PF4V1 |  |  | 12854 | -1.247 | -0.3649 | No |
| 66 | CXCL11 |  |  | 12894 | -1.256 | -0.3625 | No |
| 67 | EDA |  |  | 13370 | -1.404 | -0.3823 | No |
| 68 | IL23A |  |  | 13381 | -1.408 | -0.3784 | No |
| 69 | BMPR2 |  |  | 13384 | -1.410 | -0.3740 | No |
| 70 | RELT |  |  | 13424 | -1.424 | -0.3716 | No |
| 71 | IL23R |  |  | 13657 | -1.528 | -0.3790 | No |
| 72 | TGFB2 |  |  | 13716 | -1.553 | -0.3776 | No |
| 73 | VEGFC |  |  | 13988 | -1.683 | -0.3870 | No |
| 74 | TNFRSF10B |  |  | 14112 | -1.750 | -0.3888 | Yes |
| 75 | IL12RB2 |  |  | 14188 | -1.786 | -0.3882 | Yes |
| 76 | CCL7 |  |  | 14225 | -1.812 | -0.3856 | Yes |
| 77 | KDR |  |  | 14271 | -1.849 | -0.3835 | Yes |
| 78 | EPOR |  |  | 14307 | -1.873 | -0.3809 | Yes |
| 79 | TNFSF11 |  |  | 14309 | -1.873 | -0.3765 | Yes |
| 80 | CXCR4 |  |  | 14380 | -1.918 | -0.3756 | Yes |
| 81 | IL1B |  |  | 14481 | -1.989 | -0.3763 | Yes |
| 82 | IL2 |  |  | 14536 | -2.032 | -0.3746 | Yes |
| 83 | TNFSF8 |  |  | 14730 | -2.170 | -0.3801 | Yes |
| 84 | IL6 |  |  | 14833 | -2.246 | -0.3808 | Yes |
| 85 | ACVR2A |  |  | 14849 | -2.263 | -0.3772 | Yes |
| 86 | CSF1R |  |  | 14869 | -2.289 | -0.3737 | Yes |
| 87 | OSM |  |  | 14910 | -2.324 | -0.3713 | Yes |
| 88 | IL13RA1 |  |  | 14921 | -2.337 | -0.3674 | Yes |
| 89 | IL1R1 |  |  | 14951 | -2.374 | -0.3645 | Yes |
| 90 | MPL |  |  | 15005 | -2.420 | -0.3628 | Yes |
| 91 | CX3CR1 |  |  | 15115 | -2.575 | -0.3639 | Yes |
| 92 | CXCR2 |  |  | 15177 | -2.651 | -0.3626 | Yes |
| 93 | LEP |  |  | 15259 | -2.749 | -0.3623 | Yes |
| 94 | IFNB1 |  |  | 15262 | -2.754 | -0.3580 | Yes |
| 95 | TNFSF4 |  |  | 15470 | -3.063 | -0.3641 | Yes |
| 96 | IL12A |  |  | 15711 | -3.497 | -0.3719 | Yes |
| 97 | IL13 |  |  | 15799 | -3.655 | -0.3719 | Yes |
| 98 | TNFRSF4 |  |  | 15852 | -3.724 | -0.3702 | Yes |
| 99 | HGF |  |  | 15923 | -3.887 | -0.3693 | Yes |
| 100 | IL11RA |  |  | 16035 | -4.161 | -0.3705 | Yes |
| 101 | CXCL8 |  |  | 16237 | -4.785 | -0.3764 | Yes |
| 102 | IL4R |  |  | 16297 | -4.970 | -0.3750 | Yes |
| 103 | CXCL13 |  |  | 16323 | -5.117 | -0.3718 | Yes |
| 104 | XCL1 |  |  | 16347 | -5.196 | -0.3686 | Yes |
| 105 | AMHR2 |  |  | 16387 | -5.331 | -0.3661 | Yes |
| 106 | CXCL12 |  |  | 16394 | -5.375 | -0.3620 | Yes |
| 107 | IL26 |  |  | 16402 | -5.397 | -0.3579 | Yes |
| 108 | CSF2RA |  |  | 16406 | -5.420 | -0.3537 | Yes |
| 109 | PDGFB |  |  | 16437 | -5.498 | -0.3508 | Yes |
| 110 | PLEKHO2 |  |  | 16453 | -5.564 | -0.3471 | Yes |
| 111 | CLCF1 |  |  | 16464 | -5.599 | -0.3432 | Yes |
| 112 | IL3RA |  |  | 16521 | -5.826 | -0.3416 | Yes |
| 113 | CXCL5 |  |  | 16522 | -5.827 | -0.3372 | Yes |
| 114 | TNFRSF25 |  |  | 16570 | -6.027 | -0.3352 | Yes |
| 115 | CCR3 |  |  | 16619 | -6.266 | -0.3332 | Yes |
| 116 | IL6ST |  |  | 16638 | -6.361 | -0.3297 | Yes |
| 117 | IFNE |  |  | 16646 | -6.409 | -0.3256 | Yes |
| 118 | CCL24 |  |  | 16738 | -6.966 | -0.3258 | Yes |
| 119 | TNF |  |  | 16858 | -7.878 | -0.3275 | Yes |
| 120 | TNFRSF8 |  |  | 16931 | -8.547 | -0.3267 | Yes |
| 121 | XCR1 |  |  | 16948 | -8.707 | -0.3231 | Yes |
| 122 | IL24 |  |  | 16973 | -8.891 | -0.3199 | Yes |
| 123 | PRLR |  |  | 17044 | -9.589 | -0.3191 | Yes |
| 124 | IL1RAP |  |  | 17155 | -10.820 | -0.3203 | Yes |
| 125 | IL17RA |  |  | 17200 | -11.210 | -0.3181 | Yes |
| 126 | CD40 |  |  | 17248 | -11.890 | -0.3161 | Yes |
| 127 | MET |  |  | 17250 | -11.910 | -0.3117 | Yes |
| 128 | CXCR1 |  |  | 17285 | -12.700 | -0.3090 | Yes |
| 129 | CCL19 |  |  | 17294 | -12.780 | -0.3050 | Yes |
| 130 | TNFRSF9 |  |  | 17311 | -12.980 | -0.3014 | Yes |
| 131 | CXCR5 |  |  | 17313 | -13.020 | -0.2970 | Yes |
| 132 | CCR9 |  |  | 17358 | -13.840 | -0.2948 | Yes |
| 133 | TNFRSF14 |  |  | 17395 | -14.330 | -0.2922 | Yes |
| 134 | IFNGR1 |  |  | 17459 | -15.430 | -0.2910 | Yes |
| 135 | TNFRSF10D |  |  | 17472 | -15.530 | -0.2872 | Yes |
| 136 | IL18R1 |  |  | 17497 | -15.970 | -0.2840 | Yes |
| 137 | FLT3 |  |  | 17523 | -16.690 | -0.2809 | Yes |
| 138 | TNFRSF1B |  |  | 17534 | -17.040 | -0.2769 | Yes |
| 139 | KITLG |  |  | 17550 | -17.470 | -0.2733 | Yes |
| 140 | IL10RA |  |  | 17553 | -17.610 | -0.2690 | Yes |
| 141 | TNFRSF11A |  |  | 17591 | -18.440 | -0.2664 | Yes |
| 142 | IL12B |  |  | 17602 | -18.740 | -0.2625 | Yes |
| 143 | LIF |  |  | 17645 | -19.920 | -0.2602 | Yes |
| 144 | CCR1 |  |  | 17675 | -21.210 | -0.2573 | Yes |
| 145 | LTA |  |  | 17687 | -21.740 | -0.2534 | Yes |
| 146 | CCL8 |  |  | 17789 | -25.920 | -0.2541 | Yes |
| 147 | CCL14 |  |  | 17798 | -26.390 | -0.2501 | Yes |
| 148 | IL17A |  |  | 17893 | -30.880 | -0.2505 | Yes |
| 149 | TSLP |  |  | 17958 | -34.460 | -0.2493 | Yes |
| 150 | CCL16 |  |  | 18034 | -39.660 | -0.2487 | Yes |
| 151 | FLT4 |  |  | 18039 | -39.920 | -0.2445 | Yes |
| 152 | FAS |  |  | 18057 | -41.750 | -0.2410 | Yes |
| 153 | IL12RB1 |  |  | 18084 | -43.530 | -0.2379 | Yes |
| 154 | CRLF2 |  |  | 18114 | -46.000 | -0.2349 | Yes |
| 155 | TNFRSF10C |  |  | 18147 | -49.620 | -0.2321 | Yes |
| 156 | IL11 |  |  | 18177 | -51.740 | -0.2292 | Yes |
| 157 | TNFRSF10A |  |  | 18251 | -61.870 | -0.2285 | Yes |
| 158 | CX3CL1 |  |  | 18277 | -65.270 | -0.2253 | Yes |
| 159 | IL7R |  |  | 18311 | -70.790 | -0.2226 | Yes |
| 160 | CCL3 |  |  | 18344 | -75.620 | -0.2198 | Yes |
| 161 | XCL2 |  |  | 18346 | -76.390 | -0.2154 | Yes |
| 162 | CXCL16 |  |  | 18348 | -76.690 | -0.2110 | Yes |
| 163 | CCL20 |  |  | 18389 | -85.550 | -0.2087 | Yes |
| 164 | LEPR |  |  | 18403 | -89.040 | -0.2049 | Yes |
| 165 | TNFSF12 |  |  | 18411 | -91.350 | -0.2008 | Yes |
| 166 | IL9R |  |  | 18462 | -107.500 | -0.1990 | Yes |
| 167 | IL7 |  |  | 18472 | -111.300 | -0.1950 | Yes |
| 168 | EDAR |  |  | 18478 | -113.000 | -0.1908 | Yes |
| 169 | CSF3R |  |  | 18561 | -146.000 | -0.1906 | Yes |
| 170 | CSF3 |  |  | 18593 | -162.700 | -0.1877 | Yes |
| 171 | CCL21 |  |  | 18624 | -177.200 | -0.1848 | Yes |
| 172 | IL18 |  |  | 18627 | -178.000 | -0.1805 | Yes |
| 173 | CXCL10 |  |  | 18656 | -196.100 | -0.1775 | Yes |
| 174 | TNFSF15 |  |  | 18659 | -197.800 | -0.1732 | Yes |
| 175 | TNFSF13B |  |  | 18668 | -201.700 | -0.1692 | Yes |
| 176 | TNFRSF17 |  |  | 18699 | -219.900 | -0.1663 | Yes |
| 177 | IL10 |  |  | 18725 | -237.700 | -0.1631 | Yes |
| 178 | EDA2R |  |  | 18743 | -249.800 | -0.1596 | Yes |
| 179 | CCL11 |  |  | 18783 | -284.800 | -0.1572 | Yes |
| 180 | TGFBR2 |  |  | 18825 | -325.500 | -0.1548 | Yes |
| 181 | CCL4 |  |  | 18834 | -336.900 | -0.1508 | Yes |
| 182 | IL22RA2 |  |  | 18849 | -358.400 | -0.1471 | Yes |
| 183 | CXCL6 |  |  | 18895 | -447.300 | -0.1450 | Yes |
| 184 | CCR8 |  |  | 18947 | -578.100 | -0.1431 | Yes |
| 185 | CXCL9 |  |  | 19017 | -806.500 | -0.1422 | Yes |
| 186 | IL6R |  |  | 19127 | -1599.000 | -0.1434 | Yes |
| 187 | GDF5 |  |  | 19164 | -1979.000 | -0.1408 | Yes |
| 188 | IL5RA |  |  | 19220 | -2833.000 | -0.1392 | Yes |
| 189 | CCL2 |  |  | 19229 | -3067.000 | -0.1351 | Yes |
| 190 | IL20RA |  |  | 19230 | -3114.000 | -0.1307 | Yes |
| 191 | CCR7 |  |  | 19238 | -3261.000 | -0.1267 | Yes |
| 192 | CSF2RB |  |  | 19242 | -3340.000 | -0.1224 | Yes |
| 193 | CSF2 |  |  | 19263 | -3911.000 | -0.1190 | Yes |
| 194 | CXCL3 |  |  | 19310 | -5648.000 | -0.1169 | Yes |
| 195 | CXCL1 |  |  | 19313 | -5878.000 | -0.1126 | Yes |
| 196 | FASLG |  |  | 19371 | -10130.000 | -0.1111 | Yes |
| 197 | ACVRL1 |  |  | 19374 | -10510.000 | -0.1067 | Yes |
| 198 | KIT |  |  | 19380 | -11090.000 | -0.1026 | Yes |
| 199 | TNFSF13 |  |  | 19398 | -13390.000 | -0.0990 | Yes |
| 200 | IL2RB |  |  | 19399 | -13420.000 | -0.0946 | Yes |
| 201 | CTF1 |  |  | 19405 | -14510.000 | -0.0904 | Yes |
| 202 | CXCL2 |  |  | 19438 | -21480.000 | -0.0876 | Yes |
| 203 | IL1A |  |  | 19468 | -33230.000 | -0.0847 | Yes |
| 204 | CD27 |  |  | 19485 | -46620.000 | -0.0811 | Yes |
| 205 | CD40LG |  |  | 19510 | -66050.000 | -0.0779 | Yes |
| 206 | IL15RA |  |  | 19521 | -76760.000 | -0.0740 | Yes |
| 207 | CCR4 |  |  | 19534 | -94150.000 | -0.0701 | Yes |
| 208 | CCR2 |  |  | 19538 | -99570.000 | -0.0659 | Yes |
| 209 | TNFSF14 |  |  | 19540 | -101100.000 | -0.0615 | Yes |
| 210 | TNFSF10 |  |  | 19548 | -109200.000 | -0.0574 | Yes |
| 211 | IL18RAP |  |  | 19554 | -118600.000 | -0.0533 | Yes |
| 212 | CCR6 |  |  | 19557 | -123200.000 | -0.0489 | Yes |
| 213 | CXCR3 |  |  | 19574 | -151900.000 | -0.0453 | Yes |
| 214 | IL15 |  |  | 19608 | -273800.000 | -0.0426 | Yes |
| 215 | CXCR6 |  |  | 19651 | -547000.000 | -0.0403 | Yes |
| 216 | CCR5 |  |  | 19693 | -1522000.000 | -0.0380 | Yes |
| 217 | IFNG |  |  | 19720 | -4827000.000 | -0.0349 | Yes |
| 218 | CCL17 |  |  | 19722 | -5384000.000 | -0.0305 | Yes |
| 219 | TNFRSF6B |  |  | 19725 | -6473000.000 | -0.0262 | Yes |
| 220 | LTB |  |  | 19755 | -19520000.000 | -0.0232 | Yes |
| 221 | CCL5 |  |  | 19782 | -101400000.000 | -0.0201 | Yes |
| 222 | CCL23 |  |  | 19796 | -488300000.000 | -0.0164 | Yes |
| 223 | CCL13 |  |  | 19810 | -7880999936.000 | -0.0126 | Yes |
| 224 | CCL22 |  |  | 19819 | -271500001280.000 | -0.0086 | Yes |
| 225 | CCL18 |  |  | 19820 | -328000012288.000 | -0.0042 | Yes |
| 226 | IL2RG |  |  | 19824 | -5699000074240.000 | 0.0001 | Yes |
Table: GSEA details [plain text format]

  

Fig 2: KEGG\_CYTOKINE\_CYTOKINE\_RECEPTOR\_INTERACTION: Random ES distribution      
 Gene set null distribution of ES for **KEGG\_CYTOKINE\_CYTOKINE\_RECEPTOR\_INTERACTION**

  
