# Supplementary material for: DNA methylation-based classifier and gene expression signatures detect BRCAness in osteosarcoma
Source: PLoS Comput Biol. 2021 Nov 11;17(11):e1009562. doi: 10.1371/journal.pcbi.1009562 (PMC8584788; doi:10.1371/journal.pcbi.1009562)
Supplement: S2 File — (ZIP) [file pcbi.1009562.s002.zip › S2_File/my_analysis_Kegg.GseaPreranked.1581692187239/KEGG_DNA_REPLICATION.html]

Details for gene set KEGG\_DNA\_REPLICATION[GSEA]

|  || Dataset | DEG3\_two3dTopBottom |
| Phenotype | NoPhenotypeAvailable |
| Upregulated in class | na\_pos |
| GeneSet | KEGG\_DNA\_REPLICATION |
| Enrichment Score (ES) | 0.63909185 |
| Normalized Enrichment Score (NES) | 0.63909185 |
| Nominal p-value | 0.0 |
| FDR q-value | 0.0 |
| FWER p-Value | 0.0 |
Table: GSEA Results Summary

  

Fig 1: Enrichment plot: KEGG\_DNA\_REPLICATION      
 Profile of the Running ES Score & Positions of GeneSet Members on the Rank Ordered List

  

| PROBE | GENE SYMBOL | GENE\_TITLE | RANK IN GENE LIST | RANK METRIC SCORE | RUNNING ES | CORE ENRICHMENT || 1 | RPA3 |  |  | 240 | 880.400 | 0.0212 | Yes |
| 2 | POLD2 |  |  | 267 | 687.000 | 0.0532 | Yes |
| 3 | PRIM1 |  |  | 435 | 269.800 | 0.0781 | Yes |
| 4 | POLE2 |  |  | 914 | 62.780 | 0.0873 | Yes |
| 5 | RFC5 |  |  | 1024 | 51.250 | 0.1151 | Yes |
| 6 | POLA1 |  |  | 1329 | 34.300 | 0.1331 | Yes |
| 7 | RFC2 |  |  | 1582 | 25.130 | 0.1537 | Yes |
| 8 | FEN1 |  |  | 1643 | 23.820 | 0.1840 | Yes |
| 9 | MCM3 |  |  | 1802 | 20.500 | 0.2094 | Yes |
| 10 | RFC3 |  |  | 1843 | 19.720 | 0.2407 | Yes |
| 11 | POLD1 |  |  | 2192 | 15.340 | 0.2564 | Yes |
| 12 | MCM2 |  |  | 2194 | 15.310 | 0.2897 | Yes |
| 13 | POLE |  |  | 2249 | 14.810 | 0.3203 | Yes |
| 14 | LIG1 |  |  | 2303 | 14.320 | 0.3510 | Yes |
| 15 | RFC4 |  |  | 2390 | 13.470 | 0.3800 | Yes |
| 16 | POLA2 |  |  | 2453 | 12.990 | 0.4102 | Yes |
| 17 | MCM6 |  |  | 2797 | 10.490 | 0.4262 | Yes |
| 18 | POLD3 |  |  | 3045 | 9.243 | 0.4470 | Yes |
| 19 | PRIM2 |  |  | 3330 | 7.906 | 0.4660 | Yes |
| 20 | RPA2 |  |  | 3591 | 7.079 | 0.4862 | Yes |
| 21 | POLE4 |  |  | 3842 | 6.309 | 0.5069 | Yes |
| 22 | MCM4 |  |  | 3959 | 6.035 | 0.5344 | Yes |
| 23 | MCM7 |  |  | 4544 | 4.903 | 0.5382 | Yes |
| 24 | POLE3 |  |  | 4864 | 4.371 | 0.5555 | Yes |
| 25 | RFC1 |  |  | 5211 | 3.927 | 0.5713 | Yes |
| 26 | RPA4 |  |  | 5614 | 3.458 | 0.5844 | Yes |
| 27 | MCM5 |  |  | 5940 | 3.156 | 0.6013 | Yes |
| 28 | SSBP1 |  |  | 6396 | 2.785 | 0.6116 | Yes |
| 29 | RPA1 |  |  | 6513 | 2.702 | 0.6391 | Yes |
| 30 | POLD4 |  |  | 12325 | -1.133 | 0.3789 | No |
Table: GSEA details [plain text format]

  

Fig 2: KEGG\_DNA\_REPLICATION: Random ES distribution      
 Gene set null distribution of ES for **KEGG\_DNA\_REPLICATION**

  
