# Supplementary material for: DNA methylation-based classifier and gene expression signatures detect BRCAness in osteosarcoma
Source: PLoS Comput Biol. 2021 Nov 11;17(11):e1009562. doi: 10.1371/journal.pcbi.1009562 (PMC8584788; doi:10.1371/journal.pcbi.1009562)
Supplement: S2 File — (ZIP) [file pcbi.1009562.s002.zip › S2_File/my_analysis_Kegg.GseaPreranked.1581692187239/KEGG_DRUG_METABOLISM_CYTOCHROME_P450.html]

Details for gene set KEGG\_DRUG\_METABOLISM\_CYTOCHROME\_P450[GSEA]

|  || Dataset | DEG3\_two3dTopBottom |
| Phenotype | NoPhenotypeAvailable |
| Upregulated in class | na\_neg |
| GeneSet | KEGG\_DRUG\_METABOLISM\_CYTOCHROME\_P450 |
| Enrichment Score (ES) | -0.3442236 |
| Normalized Enrichment Score (NES) | -0.3442236 |
| Nominal p-value | 0.0 |
| FDR q-value | 0.019701594 |
| FWER p-Value | 0.23566666 |
Table: GSEA Results Summary

  

Fig 1: Enrichment plot: KEGG\_DRUG\_METABOLISM\_CYTOCHROME\_P450      
 Profile of the Running ES Score & Positions of GeneSet Members on the Rank Ordered List

  

| PROBE | GENE SYMBOL | GENE\_TITLE | RANK IN GENE LIST | RANK METRIC SCORE | RUNNING ES | CORE ENRICHMENT || 1 | GSTM3 |  |  | 309 | 522.700 | 0.0029 | No |
| 2 | GSTM4 |  |  | 1128 | 44.390 | -0.0200 | No |
| 3 | GSTA4 |  |  | 2151 | 15.750 | -0.0531 | No |
| 4 | GSTZ1 |  |  | 3021 | 9.363 | -0.0786 | No |
| 5 | GSTT2 |  |  | 3489 | 7.353 | -0.0837 | No |
| 6 | UGT2B10 |  |  | 3736 | 6.619 | -0.0776 | No |
| 7 | GSTA1 |  |  | 3759 | 6.570 | -0.0602 | No |
| 8 | GSTM1 |  |  | 3830 | 6.334 | -0.0452 | No |
| 9 | UGT1A1 |  |  | 5368 | 3.747 | -0.1044 | No |
| 10 | UGT2B11 |  |  | 5806 | 3.279 | -0.1080 | No |
| 11 | GSTM2 |  |  | 6996 | 2.412 | -0.1496 | No |
| 12 | ALDH1A3 |  |  | 8075 | 1.888 | -0.1856 | No |
| 13 | MGST3 |  |  | 8782 | 1.623 | -0.2028 | No |
| 14 | UGT2B15 |  |  | 9893 | 1.317 | -0.2404 | No |
| 15 | FMO4 |  |  | 10639 | 1.164 | -0.2596 | No |
| 16 | CYP3A43 |  |  | 11541 | 1.006 | -0.2866 | No |
| 17 | GSTP1 |  |  | 11880 | -1.047 | -0.2852 | No |
| 18 | CYP2E1 |  |  | 12990 | -1.286 | -0.3228 | No |
| 19 | GSTT1 |  |  | 13136 | -1.332 | -0.3116 | No |
| 20 | UGT2B17 |  |  | 13474 | -1.445 | -0.3101 | No |
| 21 | CYP3A4 |  |  | 13605 | -1.502 | -0.2982 | No |
| 22 | ADH5 |  |  | 14352 | -1.900 | -0.3174 | No |
| 23 | ALDH3B1 |  |  | 14884 | -2.309 | -0.3257 | Yes |
| 24 | CYP2C19 |  |  | 14964 | -2.385 | -0.3112 | Yes |
| 25 | CYP2C9 |  |  | 15214 | -2.691 | -0.3053 | Yes |
| 26 | GSTO2 |  |  | 15839 | -3.714 | -0.3183 | Yes |
| 27 | UGT2A3 |  |  | 16149 | -4.505 | -0.3154 | Yes |
| 28 | CYP2C8 |  |  | 16207 | -4.702 | -0.2998 | Yes |
| 29 | FMO1 |  |  | 16345 | -5.194 | -0.2882 | Yes |
| 30 | GSTO1 |  |  | 16523 | -5.828 | -0.2786 | Yes |
| 31 | MGST2 |  |  | 16662 | -6.482 | -0.2671 | Yes |
| 32 | ALDH3B2 |  |  | 16692 | -6.653 | -0.2500 | Yes |
| 33 | GSTK1 |  |  | 16831 | -7.672 | -0.2385 | Yes |
| 34 | UGT2B7 |  |  | 16841 | -7.754 | -0.2204 | Yes |
| 35 | CYP1A2 |  |  | 16908 | -8.308 | -0.2052 | Yes |
| 36 | MGST1 |  |  | 17165 | -10.890 | -0.1997 | Yes |
| 37 | MAOB |  |  | 17354 | -13.720 | -0.1907 | Yes |
| 38 | GSTM5 |  |  | 17532 | -16.970 | -0.1811 | Yes |
| 39 | MAOA |  |  | 17631 | -19.650 | -0.1675 | Yes |
| 40 | CYP2D6 |  |  | 17829 | -27.500 | -0.1590 | Yes |
| 41 | ADH1C |  |  | 17899 | -31.100 | -0.1439 | Yes |
| 42 | ADH4 |  |  | 17940 | -33.460 | -0.1274 | Yes |
| 43 | GSTA3 |  |  | 18454 | -105.400 | -0.1349 | Yes |
| 44 | FMO5 |  |  | 18889 | -428.700 | -0.1383 | Yes |
| 45 | FMO3 |  |  | 18897 | -448.000 | -0.1201 | Yes |
| 46 | ADH1B |  |  | 19028 | -867.000 | -0.1082 | Yes |
| 47 | UGT1A6 |  |  | 19033 | -889.800 | -0.0899 | Yes |
| 48 | ADH6 |  |  | 19092 | -1246.000 | -0.0743 | Yes |
| 49 | ADH1A |  |  | 19175 | -2102.000 | -0.0599 | Yes |
| 50 | UGT2B4 |  |  | 19278 | -4319.000 | -0.0466 | Yes |
| 51 | FMO2 |  |  | 19404 | -13970.000 | -0.0344 | Yes |
| 52 | CYP3A5 |  |  | 19526 | -85430.000 | -0.0220 | Yes |
| 53 | AOX1 |  |  | 19580 | -170600.000 | -0.0061 | Yes |
| 54 | CYP2C18 |  |  | 19594 | -204700.000 | 0.0117 | Yes |
Table: GSEA details [plain text format]

  

Fig 2: KEGG\_DRUG\_METABOLISM\_CYTOCHROME\_P450: Random ES distribution      
 Gene set null distribution of ES for **KEGG\_DRUG\_METABOLISM\_CYTOCHROME\_P450**

  
