# Supplementary material for: DNA methylation-based classifier and gene expression signatures detect BRCAness in osteosarcoma
Source: PLoS Comput Biol. 2021 Nov 11;17(11):e1009562. doi: 10.1371/journal.pcbi.1009562 (PMC8584788; doi:10.1371/journal.pcbi.1009562)
Supplement: S2 File — (ZIP) [file pcbi.1009562.s002.zip › S2_File/my_analysis_Kegg.GseaPreranked.1581692187239/KEGG_EPITHELIAL_CELL_SIGNALING_IN_HELICOBACTER_PYLORI_INFECTION.html]

Details for gene set KEGG\_EPITHELIAL\_CELL\_SIGNALING\_IN\_HELICOBACTER\_PYLORI\_INFECTION[GSEA]

|  || Dataset | DEG3\_two3dTopBottom |
| Phenotype | NoPhenotypeAvailable |
| Upregulated in class | na\_neg |
| GeneSet | KEGG\_EPITHELIAL\_CELL\_SIGNALING\_IN\_HELICOBACTER\_PYLORI\_INFECTION |
| Enrichment Score (ES) | -0.34389767 |
| Normalized Enrichment Score (NES) | -0.34389767 |
| Nominal p-value | 0.0 |
| FDR q-value | 0.018756885 |
| FWER p-Value | 0.23666666 |
Table: GSEA Results Summary

  

Fig 1: Enrichment plot: KEGG\_EPITHELIAL\_CELL\_SIGNALING\_IN\_HELICOBACTER\_PYLORI\_INFECTION      
 Profile of the Running ES Score & Positions of GeneSet Members on the Rank Ordered List

  

| PROBE | GENE SYMBOL | GENE\_TITLE | RANK IN GENE LIST | RANK METRIC SCORE | RUNNING ES | CORE ENRICHMENT || 1 | IGSF5 |  |  | 781 | 84.760 | -0.0246 | No |
| 2 | RAC1 |  |  | 1907 | 18.810 | -0.0666 | No |
| 3 | GIT1 |  |  | 2153 | 15.730 | -0.0641 | No |
| 4 | MAP2K4 |  |  | 3343 | 7.849 | -0.1093 | No |
| 5 | PAK1 |  |  | 3718 | 6.676 | -0.1133 | No |
| 6 | PLCG1 |  |  | 4095 | 5.756 | -0.1174 | No |
| 7 | RELA |  |  | 5570 | 3.508 | -0.1771 | No |
| 8 | ATP6V1B1 |  |  | 5833 | 3.248 | -0.1754 | No |
| 9 | MAPK14 |  |  | 6175 | 2.949 | -0.1778 | No |
| 10 | IKBKG |  |  | 7246 | 2.269 | -0.2170 | No |
| 11 | ATP6V1G2 |  |  | 8768 | 1.627 | -0.2790 | No |
| 12 | PTPN11 |  |  | 9667 | 1.368 | -0.3096 | No |
| 13 | MAPK9 |  |  | 9931 | 1.308 | -0.3079 | No |
| 14 | EGFR |  |  | 10302 | 1.223 | -0.3117 | No |
| 15 | ADAM17 |  |  | 10361 | 1.211 | -0.2998 | No |
| 16 | MAPK12 |  |  | 10946 | 1.108 | -0.3144 | No |
| 17 | ATP6V1C2 |  |  | 11170 | 1.069 | -0.3107 | No |
| 18 | MAP3K14 |  |  | 11726 | -1.023 | -0.3239 | No |
| 19 | PTPRZ1 |  |  | 11991 | -1.068 | -0.3223 | No |
| 20 | SRC |  |  | 12418 | -1.153 | -0.3290 | Yes |
| 21 | CDC42 |  |  | 12678 | -1.208 | -0.3272 | Yes |
| 22 | ATP6V0A2 |  |  | 12755 | -1.223 | -0.3161 | Yes |
| 23 | CSK |  |  | 12901 | -1.259 | -0.3085 | Yes |
| 24 | JAM3 |  |  | 13070 | -1.311 | -0.3021 | Yes |
| 25 | ADAM10 |  |  | 13208 | -1.356 | -0.2941 | Yes |
| 26 | ATP6V0B |  |  | 13303 | -1.383 | -0.2839 | Yes |
| 27 | ATP6V0A1 |  |  | 13396 | -1.415 | -0.2736 | Yes |
| 28 | ATP6V0E2 |  |  | 13402 | -1.417 | -0.2590 | Yes |
| 29 | CHUK |  |  | 13437 | -1.430 | -0.2458 | Yes |
| 30 | MAPK10 |  |  | 13575 | -1.489 | -0.2378 | Yes |
| 31 | ATP6AP1 |  |  | 13680 | -1.538 | -0.2281 | Yes |
| 32 | CASP3 |  |  | 14090 | -1.736 | -0.2339 | Yes |
| 33 | ATP6V0D1 |  |  | 14105 | -1.747 | -0.2197 | Yes |
| 34 | ATP6V1D |  |  | 14733 | -2.172 | -0.2365 | Yes |
| 35 | ATP6V1E1 |  |  | 14932 | -2.349 | -0.2316 | Yes |
| 36 | JAM2 |  |  | 15072 | -2.521 | -0.2237 | Yes |
| 37 | ATP6V1F |  |  | 15121 | -2.586 | -0.2112 | Yes |
| 38 | NOD1 |  |  | 15175 | -2.650 | -0.1989 | Yes |
| 39 | CXCR2 |  |  | 15177 | -2.651 | -0.1841 | Yes |
| 40 | JUN |  |  | 15436 | -3.002 | -0.1822 | Yes |
| 41 | TJP1 |  |  | 15732 | -3.532 | -0.1822 | Yes |
| 42 | ATP6V1G1 |  |  | 15877 | -3.785 | -0.1746 | Yes |
| 43 | PLCG2 |  |  | 16000 | -4.087 | -0.1658 | Yes |
| 44 | HBEGF |  |  | 16211 | -4.708 | -0.1615 | Yes |
| 45 | CXCL8 |  |  | 16237 | -4.785 | -0.1478 | Yes |
| 46 | MAPK8 |  |  | 16782 | -7.291 | -0.1604 | Yes |
| 47 | LYN |  |  | 16798 | -7.416 | -0.1463 | Yes |
| 48 | ATP6V0C |  |  | 16829 | -7.662 | -0.1329 | Yes |
| 49 | ATP6V0E1 |  |  | 17157 | -10.830 | -0.1345 | Yes |
| 50 | ATP6V1A |  |  | 17186 | -11.040 | -0.1210 | Yes |
| 51 | MET |  |  | 17250 | -11.910 | -0.1093 | Yes |
| 52 | CXCR1 |  |  | 17285 | -12.700 | -0.0960 | Yes |
| 53 | ATP6V0D2 |  |  | 17318 | -13.050 | -0.0827 | Yes |
| 54 | ATP6V1H |  |  | 17327 | -13.200 | -0.0682 | Yes |
| 55 | IKBKB |  |  | 17407 | -14.590 | -0.0573 | Yes |
| 56 | MAPK11 |  |  | 17413 | -14.730 | -0.0426 | Yes |
| 57 | TCIRG1 |  |  | 17490 | -15.830 | -0.0315 | Yes |
| 58 | ATP6V1C1 |  |  | 17793 | -26.260 | -0.0319 | Yes |
| 59 | ATP6V1E2 |  |  | 18064 | -42.190 | -0.0306 | Yes |
| 60 | NFKB1 |  |  | 18187 | -53.080 | -0.0219 | Yes |
| 61 | NFKBIA |  |  | 18315 | -71.000 | -0.0134 | Yes |
| 62 | ATP6V1B2 |  |  | 18381 | -83.510 | -0.0018 | Yes |
| 63 | MAPK13 |  |  | 18639 | -189.500 | 0.0002 | Yes |
| 64 | ATP6V0A4 |  |  | 19272 | -4202.000 | -0.0169 | Yes |
| 65 | CXCL1 |  |  | 19313 | -5878.000 | -0.0040 | Yes |
| 66 | F11R |  |  | 19743 | -10770000.000 | -0.0108 | Yes |
| 67 | CCL5 |  |  | 19782 | -101400000.000 | 0.0022 | Yes |
Table: GSEA details [plain text format]

  

Fig 2: KEGG\_EPITHELIAL\_CELL\_SIGNALING\_IN\_HELICOBACTER\_PYLORI\_INFECTION: Random ES distribution      
 Gene set null distribution of ES for **KEGG\_EPITHELIAL\_CELL\_SIGNALING\_IN\_HELICOBACTER\_PYLORI\_INFECTION**

  
