# Supplementary material for: DNA methylation-based classifier and gene expression signatures detect BRCAness in osteosarcoma
Source: PLoS Comput Biol. 2021 Nov 11;17(11):e1009562. doi: 10.1371/journal.pcbi.1009562 (PMC8584788; doi:10.1371/journal.pcbi.1009562)
Supplement: S2 File — (ZIP) [file pcbi.1009562.s002.zip › S2_File/my_analysis_Kegg.GseaPreranked.1581692187239/KEGG_FRUCTOSE_AND_MANNOSE_METABOLISM.html]

Details for gene set KEGG\_FRUCTOSE\_AND\_MANNOSE\_METABOLISM[GSEA]

|  || Dataset | DEG3\_two3dTopBottom |
| Phenotype | NoPhenotypeAvailable |
| Upregulated in class | na\_pos |
| GeneSet | KEGG\_FRUCTOSE\_AND\_MANNOSE\_METABOLISM |
| Enrichment Score (ES) | 0.26462105 |
| Normalized Enrichment Score (NES) | 0.26462105 |
| Nominal p-value | 0.015696533 |
| FDR q-value | 0.07973772 |
| FWER p-Value | 0.863 |
Table: GSEA Results Summary

  

Fig 1: Enrichment plot: KEGG\_FRUCTOSE\_AND\_MANNOSE\_METABOLISM      
 Profile of the Running ES Score & Positions of GeneSet Members on the Rank Ordered List

  

| PROBE | GENE SYMBOL | GENE\_TITLE | RANK IN GENE LIST | RANK METRIC SCORE | RUNNING ES | CORE ENRICHMENT || 1 | TPI1 |  |  | 523 | 182.400 | 0.0039 | Yes |
| 2 | ALDOA |  |  | 732 | 96.960 | 0.0237 | Yes |
| 3 | MPI |  |  | 835 | 74.850 | 0.0488 | Yes |
| 4 | HK2 |  |  | 1076 | 47.580 | 0.0670 | Yes |
| 5 | PFKFB1 |  |  | 1079 | 47.480 | 0.0972 | Yes |
| 6 | GMPPA |  |  | 1463 | 29.140 | 0.1082 | Yes |
| 7 | PHPT1 |  |  | 1704 | 22.450 | 0.1263 | Yes |
| 8 | PFKL |  |  | 2165 | 15.650 | 0.1334 | Yes |
| 9 | PMM2 |  |  | 2621 | 11.570 | 0.1407 | Yes |
| 10 | ALDOC |  |  | 2759 | 10.680 | 0.1641 | Yes |
| 11 | PFKP |  |  | 3743 | 6.600 | 0.1447 | Yes |
| 12 | PFKFB4 |  |  | 3810 | 6.417 | 0.1717 | Yes |
| 13 | AKR1B10 |  |  | 3911 | 6.152 | 0.1970 | Yes |
| 14 | TSTA3 |  |  | 4152 | 5.653 | 0.2151 | Yes |
| 15 | FPGT |  |  | 4469 | 5.039 | 0.2295 | Yes |
| 16 | GMDS |  |  | 4738 | 4.586 | 0.2462 | Yes |
| 17 | GMPPB |  |  | 4975 | 4.234 | 0.2646 | Yes |
| 18 | SORD |  |  | 6452 | 2.744 | 0.2204 | No |
| 19 | MTMR1 |  |  | 7374 | 2.208 | 0.2041 | No |
| 20 | PFKM |  |  | 7417 | 2.190 | 0.2323 | No |
| 21 | KHK |  |  | 8136 | 1.857 | 0.2263 | No |
| 22 | FBP2 |  |  | 9332 | 1.458 | 0.1963 | No |
| 23 | ALDOB |  |  | 11548 | 1.005 | 0.1147 | No |
| 24 | PFKFB3 |  |  | 11746 | -1.026 | 0.1350 | No |
| 25 | MTMR2 |  |  | 12220 | -1.110 | 0.1414 | No |
| 26 | PMM1 |  |  | 12864 | -1.250 | 0.1392 | No |
| 27 | AKR1B1 |  |  | 13675 | -1.537 | 0.1286 | No |
| 28 | HK1 |  |  | 14303 | -1.871 | 0.1273 | No |
| 29 | MTMR6 |  |  | 15343 | -2.860 | 0.1051 | No |
| 30 | FBP1 |  |  | 17504 | -16.220 | 0.0262 | No |
| 31 | MTMR7 |  |  | 18285 | -66.550 | 0.0171 | No |
| 32 | HK3 |  |  | 18881 | -417.800 | 0.0174 | No |
| 33 | PFKFB2 |  |  | 18948 | -583.800 | 0.0444 | No |
Table: GSEA details [plain text format]

  

Fig 2: KEGG\_FRUCTOSE\_AND\_MANNOSE\_METABOLISM: Random ES distribution      
 Gene set null distribution of ES for **KEGG\_FRUCTOSE\_AND\_MANNOSE\_METABOLISM**

  
