# Supplementary material for: DNA methylation-based classifier and gene expression signatures detect BRCAness in osteosarcoma
Source: PLoS Comput Biol. 2021 Nov 11;17(11):e1009562. doi: 10.1371/journal.pcbi.1009562 (PMC8584788; doi:10.1371/journal.pcbi.1009562)
Supplement: S2 File — (ZIP) [file pcbi.1009562.s002.zip › S2_File/my_analysis_Kegg.GseaPreranked.1581692187239/KEGG_GLYCOSAMINOGLYCAN_BIOSYNTHESIS_CHONDROITIN_SULFATE.html]

Details for gene set KEGG\_GLYCOSAMINOGLYCAN\_BIOSYNTHESIS\_CHONDROITIN\_SULFATE[GSEA]

|  || Dataset | DEG3\_two3dTopBottom |
| Phenotype | NoPhenotypeAvailable |
| Upregulated in class | na\_pos |
| GeneSet | KEGG\_GLYCOSAMINOGLYCAN\_BIOSYNTHESIS\_CHONDROITIN\_SULFATE |
| Enrichment Score (ES) | 0.2522341 |
| Normalized Enrichment Score (NES) | 0.2522341 |
| Nominal p-value | 0.12141883 |
| FDR q-value | 0.095115975 |
| FWER p-Value | 0.929 |
Table: GSEA Results Summary

  

Fig 1: Enrichment plot: KEGG\_GLYCOSAMINOGLYCAN\_BIOSYNTHESIS\_CHONDROITIN\_SULFATE      
 Profile of the Running ES Score & Positions of GeneSet Members on the Rank Ordered List

  

| PROBE | GENE SYMBOL | GENE\_TITLE | RANK IN GENE LIST | RANK METRIC SCORE | RUNNING ES | CORE ENRICHMENT || 1 | XYLT2 |  |  | 479 | 226.700 | 0.0258 | Yes |
| 2 | CHST3 |  |  | 689 | 107.900 | 0.0653 | Yes |
| 3 | CHST12 |  |  | 1469 | 28.960 | 0.0759 | Yes |
| 4 | CHPF |  |  | 1713 | 22.330 | 0.1137 | Yes |
| 5 | B4GALT7 |  |  | 2354 | 13.720 | 0.1314 | Yes |
| 6 | CHSY1 |  |  | 4373 | 5.226 | 0.0795 | Yes |
| 7 | B3GAT3 |  |  | 5199 | 3.945 | 0.0878 | Yes |
| 8 | XYLT1 |  |  | 7492 | 2.153 | 0.0221 | Yes |
| 9 | CHST13 |  |  | 7570 | 2.114 | 0.0682 | Yes |
| 10 | CHSY3 |  |  | 7731 | 2.042 | 0.1101 | Yes |
| 11 | CHST14 |  |  | 8018 | 1.919 | 0.1457 | Yes |
| 12 | CHST15 |  |  | 9033 | 1.543 | 0.1445 | Yes |
| 13 | CHPF2 |  |  | 10060 | 1.276 | 0.1427 | Yes |
| 14 | B3GAT2 |  |  | 10498 | 1.185 | 0.1706 | Yes |
| 15 | CHST11 |  |  | 11262 | 1.053 | 0.1821 | Yes |
| 16 | UST |  |  | 11284 | 1.049 | 0.2311 | Yes |
| 17 | B3GALT6 |  |  | 12708 | -1.214 | 0.2092 | Yes |
| 18 | CHST7 |  |  | 13851 | -1.618 | 0.2016 | Yes |
| 19 | DSE |  |  | 14589 | -2.065 | 0.2144 | Yes |
| 20 | B3GAT1 |  |  | 14830 | -2.245 | 0.2522 | Yes |
Table: GSEA details [plain text format]

  

Fig 2: KEGG\_GLYCOSAMINOGLYCAN\_BIOSYNTHESIS\_CHONDROITIN\_SULFATE: Random ES distribution      
 Gene set null distribution of ES for **KEGG\_GLYCOSAMINOGLYCAN\_BIOSYNTHESIS\_CHONDROITIN\_SULFATE**

  
