# Supplementary material for: DNA methylation-based classifier and gene expression signatures detect BRCAness in osteosarcoma
Source: PLoS Comput Biol. 2021 Nov 11;17(11):e1009562. doi: 10.1371/journal.pcbi.1009562 (PMC8584788; doi:10.1371/journal.pcbi.1009562)
Supplement: S2 File — (ZIP) [file pcbi.1009562.s002.zip › S2_File/my_analysis_Kegg.GseaPreranked.1581692187239/KEGG_GLYCOSPHINGOLIPID_BIOSYNTHESIS_LACTO_AND_NEOLACTO_SERIES.html]

Details for gene set KEGG\_GLYCOSPHINGOLIPID\_BIOSYNTHESIS\_LACTO\_AND\_NEOLACTO\_SERIES[GSEA]

|  || Dataset | DEG3\_two3dTopBottom |
| Phenotype | NoPhenotypeAvailable |
| Upregulated in class | na\_neg |
| GeneSet | KEGG\_GLYCOSPHINGOLIPID\_BIOSYNTHESIS\_LACTO\_AND\_NEOLACTO\_SERIES |
| Enrichment Score (ES) | -0.36665976 |
| Normalized Enrichment Score (NES) | -0.36665976 |
| Nominal p-value | 0.0 |
| FDR q-value | 0.012109757 |
| FWER p-Value | 0.13666667 |
Table: GSEA Results Summary

  

Fig 1: Enrichment plot: KEGG\_GLYCOSPHINGOLIPID\_BIOSYNTHESIS\_LACTO\_AND\_NEOLACTO\_SERIES      
 Profile of the Running ES Score & Positions of GeneSet Members on the Rank Ordered List

  

| PROBE | GENE SYMBOL | GENE\_TITLE | RANK IN GENE LIST | RANK METRIC SCORE | RUNNING ES | CORE ENRICHMENT || 1 | B3GNT4 |  |  | 1518 | 27.290 | -0.0382 | No |
| 2 | B4GALT2 |  |  | 1669 | 23.350 | -0.0073 | No |
| 3 | B4GALT3 |  |  | 2380 | 13.520 | -0.0047 | No |
| 4 | B4GAT1 |  |  | 4246 | 5.465 | -0.0604 | No |
| 5 | B4GALT4 |  |  | 5172 | 3.979 | -0.0687 | No |
| 6 | B3GALT5 |  |  | 8047 | 1.900 | -0.1754 | No |
| 7 | ST3GAL3 |  |  | 8218 | 1.825 | -0.1455 | No |
| 8 | B4GALT1 |  |  | 9948 | 1.303 | -0.1944 | No |
| 9 | ST8SIA1 |  |  | 11110 | 1.081 | -0.2145 | No |
| 10 | ST3GAL4 |  |  | 12598 | -1.190 | -0.2512 | No |
| 11 | FUT6 |  |  | 14886 | -2.310 | -0.3282 | Yes |
| 12 | FUT4 |  |  | 14935 | -2.353 | -0.2922 | Yes |
| 13 | ST3GAL6 |  |  | 15090 | -2.541 | -0.2615 | Yes |
| 14 | FUT2 |  |  | 15600 | -3.266 | -0.2487 | Yes |
| 15 | B3GALT1 |  |  | 15607 | -3.284 | -0.2106 | Yes |
| 16 | FUT5 |  |  | 16298 | -4.981 | -0.2069 | Yes |
| 17 | ABO |  |  | 16452 | -5.559 | -0.1762 | Yes |
| 18 | B3GNT2 |  |  | 17116 | -10.470 | -0.1712 | Yes |
| 19 | B3GALT2 |  |  | 17622 | -19.450 | -0.1583 | Yes |
| 20 | B3GNT3 |  |  | 18099 | -44.490 | -0.1439 | Yes |
| 21 | FUT9 |  |  | 18799 | -295.100 | -0.1407 | Yes |
| 22 | B3GNT5 |  |  | 18913 | -494.100 | -0.1079 | Yes |
| 23 | FUT3 |  |  | 18934 | -535.100 | -0.0705 | Yes |
| 24 | FUT1 |  |  | 19240 | -3296.000 | -0.0474 | Yes |
| 25 | FUT7 |  |  | 19325 | -6296.000 | -0.0132 | Yes |
| 26 | GCNT2 |  |  | 19341 | -7465.000 | 0.0245 | Yes |
Table: GSEA details [plain text format]

  

Fig 2: KEGG\_GLYCOSPHINGOLIPID\_BIOSYNTHESIS\_LACTO\_AND\_NEOLACTO\_SERIES: Random ES distribution      
 Gene set null distribution of ES for **KEGG\_GLYCOSPHINGOLIPID\_BIOSYNTHESIS\_LACTO\_AND\_NEOLACTO\_SERIES**

  
