# Supplementary material for: DNA methylation-based classifier and gene expression signatures detect BRCAness in osteosarcoma
Source: PLoS Comput Biol. 2021 Nov 11;17(11):e1009562. doi: 10.1371/journal.pcbi.1009562 (PMC8584788; doi:10.1371/journal.pcbi.1009562)
Supplement: S2 File — (ZIP) [file pcbi.1009562.s002.zip › S2_File/my_analysis_Kegg.GseaPreranked.1581692187239/KEGG_GLYCOSYLPHOSPHATIDYLINOSITOL_GPI_ANCHOR_BIOSYNTHESIS.html]

Details for gene set KEGG\_GLYCOSYLPHOSPHATIDYLINOSITOL\_GPI\_ANCHOR\_BIOSYNTHESIS[GSEA]

|  || Dataset | DEG3\_two3dTopBottom |
| Phenotype | NoPhenotypeAvailable |
| Upregulated in class | na\_pos |
| GeneSet | KEGG\_GLYCOSYLPHOSPHATIDYLINOSITOL\_GPI\_ANCHOR\_BIOSYNTHESIS |
| Enrichment Score (ES) | 0.37234262 |
| Normalized Enrichment Score (NES) | 0.37234262 |
| Nominal p-value | 0.0013477089 |
| FDR q-value | 0.011674624 |
| FWER p-Value | 0.114 |
Table: GSEA Results Summary

  

Fig 1: Enrichment plot: KEGG\_GLYCOSYLPHOSPHATIDYLINOSITOL\_GPI\_ANCHOR\_BIOSYNTHESIS      
 Profile of the Running ES Score & Positions of GeneSet Members on the Rank Ordered List

  

| PROBE | GENE SYMBOL | GENE\_TITLE | RANK IN GENE LIST | RANK METRIC SCORE | RUNNING ES | CORE ENRICHMENT || 1 | PIGL |  |  | 77 | 8463.000 | 0.0378 | Yes |
| 2 | GPAA1 |  |  | 459 | 245.000 | 0.0602 | Yes |
| 3 | PIGW |  |  | 681 | 109.600 | 0.0907 | Yes |
| 4 | PIGP |  |  | 1097 | 46.300 | 0.1114 | Yes |
| 5 | PIGU |  |  | 1561 | 25.960 | 0.1297 | Yes |
| 6 | PIGM |  |  | 2094 | 16.500 | 0.1445 | Yes |
| 7 | PIGN |  |  | 2472 | 12.800 | 0.1671 | Yes |
| 8 | PIGK |  |  | 2685 | 11.130 | 0.1981 | Yes |
| 9 | PIGF |  |  | 3265 | 8.124 | 0.2105 | Yes |
| 10 | PIGS |  |  | 3386 | 7.667 | 0.2461 | Yes |
| 11 | PIGT |  |  | 3514 | 7.296 | 0.2814 | Yes |
| 12 | DPM2 |  |  | 3744 | 6.599 | 0.3115 | Yes |
| 13 | PIGZ |  |  | 4976 | 4.232 | 0.2910 | Yes |
| 14 | PIGC |  |  | 5719 | 3.364 | 0.2952 | Yes |
| 15 | PIGO |  |  | 5827 | 3.257 | 0.3315 | Yes |
| 16 | PIGX |  |  | 7363 | 2.212 | 0.2956 | Yes |
| 17 | PIGH |  |  | 7849 | 1.990 | 0.3128 | Yes |
| 18 | GPLD1 |  |  | 8257 | 1.808 | 0.3339 | Yes |
| 19 | PIGG |  |  | 8992 | 1.556 | 0.3385 | Yes |
| 20 | PGAP1 |  |  | 9148 | 1.510 | 0.3723 | Yes |
| 21 | PIGV |  |  | 13695 | -1.542 | 0.1844 | No |
| 22 | PIGB |  |  | 14538 | -2.034 | 0.1836 | No |
| 23 | PIGQ |  |  | 15689 | -3.451 | 0.1672 | No |
| 24 | PIGA |  |  | 15997 | -4.078 | 0.1934 | No |
Table: GSEA details [plain text format]

  

Fig 2: KEGG\_GLYCOSYLPHOSPHATIDYLINOSITOL\_GPI\_ANCHOR\_BIOSYNTHESIS: Random ES distribution      
 Gene set null distribution of ES for **KEGG\_GLYCOSYLPHOSPHATIDYLINOSITOL\_GPI\_ANCHOR\_BIOSYNTHESIS**

  
