# Supplementary material for: DNA methylation-based classifier and gene expression signatures detect BRCAness in osteosarcoma
Source: PLoS Comput Biol. 2021 Nov 11;17(11):e1009562. doi: 10.1371/journal.pcbi.1009562 (PMC8584788; doi:10.1371/journal.pcbi.1009562)
Supplement: S2 File — (ZIP) [file pcbi.1009562.s002.zip › S2_File/my_analysis_Kegg.GseaPreranked.1581692187239/KEGG_HEMATOPOIETIC_CELL_LINEAGE.html]

Details for gene set KEGG\_HEMATOPOIETIC\_CELL\_LINEAGE[GSEA]

|  || Dataset | DEG3\_two3dTopBottom |
| Phenotype | NoPhenotypeAvailable |
| Upregulated in class | na\_neg |
| GeneSet | KEGG\_HEMATOPOIETIC\_CELL\_LINEAGE |
| Enrichment Score (ES) | -0.54880613 |
| Normalized Enrichment Score (NES) | -0.54880613 |
| Nominal p-value | 0.0 |
| FDR q-value | 0.0 |
| FWER p-Value | 0.0 |
Table: GSEA Results Summary

  

Fig 1: Enrichment plot: KEGG\_HEMATOPOIETIC\_CELL\_LINEAGE      
 Profile of the Running ES Score & Positions of GeneSet Members on the Rank Ordered List

  

| PROBE | GENE SYMBOL | GENE\_TITLE | RANK IN GENE LIST | RANK METRIC SCORE | RUNNING ES | CORE ENRICHMENT || 1 | TPO |  |  | 4218 | 5.532 | -0.2011 | No |
| 2 | EPO |  |  | 4813 | 4.460 | -0.2187 | No |
| 3 | ITGA2B |  |  | 6851 | 2.507 | -0.3093 | No |
| 4 | FLT3LG |  |  | 8905 | 1.585 | -0.4008 | No |
| 5 | ITGA4 |  |  | 9100 | 1.522 | -0.3981 | No |
| 6 | TFRC |  |  | 9151 | 1.510 | -0.3882 | No |
| 7 | CSF1 |  |  | 10023 | 1.284 | -0.4198 | No |
| 8 | IL2RA |  |  | 10115 | 1.263 | -0.4119 | No |
| 9 | CD9 |  |  | 10141 | 1.258 | -0.4006 | No |
| 10 | CD1A |  |  | 10175 | 1.251 | -0.3898 | No |
| 11 | IL1R2 |  |  | 10747 | 1.145 | -0.4062 | No |
| 12 | ITGA5 |  |  | 10881 | 1.120 | -0.4005 | No |
| 13 | ITGA6 |  |  | 11264 | 1.053 | -0.4073 | No |
| 14 | CD14 |  |  | 11412 | 1.025 | -0.4023 | No |
| 15 | EPOR |  |  | 14307 | -1.873 | -0.5363 | Yes |
| 16 | CD7 |  |  | 14422 | -1.948 | -0.5296 | Yes |
| 17 | IL1B |  |  | 14481 | -1.989 | -0.5200 | Yes |
| 18 | MME |  |  | 14512 | -2.011 | -0.5090 | Yes |
| 19 | IL6 |  |  | 14833 | -2.246 | -0.5127 | Yes |
| 20 | CSF1R |  |  | 14869 | -2.289 | -0.5020 | Yes |
| 21 | IL1R1 |  |  | 14951 | -2.374 | -0.4936 | Yes |
| 22 | THPO |  |  | 15028 | -2.446 | -0.4850 | Yes |
| 23 | ITGB3 |  |  | 15767 | -3.601 | -0.5098 | Yes |
| 24 | GP9 |  |  | 15781 | -3.618 | -0.4980 | Yes |
| 25 | CD34 |  |  | 15806 | -3.664 | -0.4867 | Yes |
| 26 | IL11RA |  |  | 16035 | -4.161 | -0.4858 | Yes |
| 27 | CD36 |  |  | 16224 | -4.755 | -0.4828 | Yes |
| 28 | IL4R |  |  | 16297 | -4.970 | -0.4739 | Yes |
| 29 | CD1D |  |  | 16303 | -5.010 | -0.4617 | Yes |
| 30 | CSF2RA |  |  | 16406 | -5.420 | -0.4543 | Yes |
| 31 | GYPA |  |  | 16412 | -5.449 | -0.4421 | Yes |
| 32 | GP1BA |  |  | 16504 | -5.755 | -0.4342 | Yes |
| 33 | FCGR1A |  |  | 16511 | -5.783 | -0.4220 | Yes |
| 34 | IL3RA |  |  | 16521 | -5.826 | -0.4100 | Yes |
| 35 | TNF |  |  | 16858 | -7.878 | -0.4145 | Yes |
| 36 | CD59 |  |  | 16867 | -7.978 | -0.4024 | Yes |
| 37 | ITGA1 |  |  | 16979 | -8.920 | -0.3955 | Yes |
| 38 | GP5 |  |  | 17048 | -9.646 | -0.3864 | Yes |
| 39 | CD44 |  |  | 17274 | -12.320 | -0.3853 | Yes |
| 40 | CD19 |  |  | 17406 | -14.570 | -0.3795 | Yes |
| 41 | FLT3 |  |  | 17523 | -16.690 | -0.3728 | Yes |
| 42 | ITGAM |  |  | 17547 | -17.420 | -0.3615 | Yes |
| 43 | KITLG |  |  | 17550 | -17.470 | -0.3491 | Yes |
| 44 | ITGA2 |  |  | 17665 | -20.600 | -0.3424 | Yes |
| 45 | CR1 |  |  | 17742 | -24.130 | -0.3337 | Yes |
| 46 | ANPEP |  |  | 17747 | -24.300 | -0.3214 | Yes |
| 47 | CD33 |  |  | 17828 | -27.500 | -0.3130 | Yes |
| 48 | IL11 |  |  | 18177 | -51.740 | -0.3181 | Yes |
| 49 | MS4A1 |  |  | 18272 | -64.660 | -0.3104 | Yes |
| 50 | IL7R |  |  | 18311 | -70.790 | -0.2998 | Yes |
| 51 | HLA-DRB1 |  |  | 18392 | -86.860 | -0.2913 | Yes |
| 52 | IL9R |  |  | 18462 | -107.500 | -0.2823 | Yes |
| 53 | IL7 |  |  | 18472 | -111.300 | -0.2703 | Yes |
| 54 | CD38 |  |  | 18498 | -120.800 | -0.2591 | Yes |
| 55 | CD55 |  |  | 18544 | -138.300 | -0.2488 | Yes |
| 56 | CSF3R |  |  | 18561 | -146.000 | -0.2372 | Yes |
| 57 | CSF3 |  |  | 18593 | -162.700 | -0.2262 | Yes |
| 58 | CD37 |  |  | 18714 | -229.000 | -0.2198 | Yes |
| 59 | HLA-DRA |  |  | 18720 | -232.200 | -0.2076 | Yes |
| 60 | HLA-DRB5 |  |  | 18733 | -243.500 | -0.1957 | Yes |
| 61 | CD4 |  |  | 18780 | -283.000 | -0.1855 | Yes |
| 62 | CD8B |  |  | 18793 | -289.900 | -0.1736 | Yes |
| 63 | FCER2 |  |  | 18848 | -357.100 | -0.1638 | Yes |
| 64 | IL6R |  |  | 19127 | -1599.000 | -0.1654 | Yes |
| 65 | CR2 |  |  | 19147 | -1767.000 | -0.1539 | Yes |
| 66 | CD22 |  |  | 19215 | -2706.000 | -0.1448 | Yes |
| 67 | IL5RA |  |  | 19220 | -2833.000 | -0.1325 | Yes |
| 68 | CSF2 |  |  | 19263 | -3911.000 | -0.1221 | Yes |
| 69 | KIT |  |  | 19380 | -11090.000 | -0.1155 | Yes |
| 70 | ITGA3 |  |  | 19384 | -11660.000 | -0.1031 | Yes |
| 71 | CD3E |  |  | 19417 | -16680.000 | -0.0922 | Yes |
| 72 | CD3D |  |  | 19448 | -25370.000 | -0.0813 | Yes |
| 73 | IL1A |  |  | 19468 | -33230.000 | -0.0697 | Yes |
| 74 | CD1E |  |  | 19487 | -47750.000 | -0.0581 | Yes |
| 75 | CD8A |  |  | 19544 | -106100.000 | -0.0485 | Yes |
| 76 | CD2 |  |  | 19585 | -188800.000 | -0.0380 | Yes |
| 77 | CD3G |  |  | 19586 | -189000.000 | -0.0255 | Yes |
| 78 | CD1C |  |  | 19636 | -426700.000 | -0.0155 | Yes |
| 79 | CD1B |  |  | 19690 | -1456000.000 | -0.0057 | Yes |
| 80 | CD5 |  |  | 19698 | -1631000.000 | 0.0065 | Yes |
Table: GSEA details [plain text format]

  

Fig 2: KEGG\_HEMATOPOIETIC\_CELL\_LINEAGE: Random ES distribution      
 Gene set null distribution of ES for **KEGG\_HEMATOPOIETIC\_CELL\_LINEAGE**

  
