# Supplementary material for: DNA methylation-based classifier and gene expression signatures detect BRCAness in osteosarcoma
Source: PLoS Comput Biol. 2021 Nov 11;17(11):e1009562. doi: 10.1371/journal.pcbi.1009562 (PMC8584788; doi:10.1371/journal.pcbi.1009562)
Supplement: S2 File — (ZIP) [file pcbi.1009562.s002.zip › S2_File/my_analysis_Kegg.GseaPreranked.1581692187239/KEGG_HOMOLOGOUS_RECOMBINATION.html]

Details for gene set KEGG\_HOMOLOGOUS\_RECOMBINATION[GSEA]

|  || Dataset | DEG3\_two3dTopBottom |
| Phenotype | NoPhenotypeAvailable |
| Upregulated in class | na\_pos |
| GeneSet | KEGG\_HOMOLOGOUS\_RECOMBINATION |
| Enrichment Score (ES) | 0.48192987 |
| Normalized Enrichment Score (NES) | 0.48192987 |
| Nominal p-value | 0.0 |
| FDR q-value | 9.932596E-4 |
| FWER p-Value | 0.004333333 |
Table: GSEA Results Summary

  

Fig 1: Enrichment plot: KEGG\_HOMOLOGOUS\_RECOMBINATION      
 Profile of the Running ES Score & Positions of GeneSet Members on the Rank Ordered List

  

| PROBE | GENE SYMBOL | GENE\_TITLE | RANK IN GENE LIST | RANK METRIC SCORE | RUNNING ES | CORE ENRICHMENT || 1 | EME1 |  |  | 13 | 1481000.000 | 0.0378 | Yes |
| 2 | TOP3A |  |  | 54 | 18930.000 | 0.0742 | Yes |
| 3 | RPA3 |  |  | 240 | 880.400 | 0.1034 | Yes |
| 4 | POLD2 |  |  | 267 | 687.000 | 0.1405 | Yes |
| 5 | BLM |  |  | 297 | 560.400 | 0.1775 | Yes |
| 6 | RAD51 |  |  | 1336 | 34.020 | 0.1636 | Yes |
| 7 | RAD54L |  |  | 1798 | 20.550 | 0.1787 | Yes |
| 8 | RAD51D |  |  | 1953 | 18.260 | 0.2094 | Yes |
| 9 | POLD1 |  |  | 2192 | 15.340 | 0.2359 | Yes |
| 10 | RAD54B |  |  | 2729 | 10.840 | 0.2472 | Yes |
| 11 | RAD51B |  |  | 2808 | 10.460 | 0.2818 | Yes |
| 12 | POLD3 |  |  | 3045 | 9.243 | 0.3083 | Yes |
| 13 | RPA2 |  |  | 3591 | 7.079 | 0.3193 | Yes |
| 14 | RAD52 |  |  | 3707 | 6.730 | 0.3519 | Yes |
| 15 | RPA4 |  |  | 5614 | 3.458 | 0.2941 | Yes |
| 16 | XRCC3 |  |  | 5705 | 3.379 | 0.3280 | Yes |
| 17 | BRCA2 |  |  | 5919 | 3.173 | 0.3557 | Yes |
| 18 | XRCC2 |  |  | 6130 | 2.986 | 0.3836 | Yes |
| 19 | SSBP1 |  |  | 6396 | 2.785 | 0.4087 | Yes |
| 20 | RPA1 |  |  | 6513 | 2.702 | 0.4413 | Yes |
| 21 | MUS81 |  |  | 7071 | 2.367 | 0.4516 | Yes |
| 22 | RAD51C |  |  | 7233 | 2.277 | 0.4819 | Yes |
| 23 | NBN |  |  | 8739 | 1.634 | 0.4444 | No |
| 24 | TOP3B |  |  | 10325 | 1.219 | 0.4028 | No |
| 25 | POLD4 |  |  | 12325 | -1.133 | 0.3403 | No |
| 26 | RAD50 |  |  | 13086 | -1.317 | 0.3404 | No |
Table: GSEA details [plain text format]

  

Fig 2: KEGG\_HOMOLOGOUS\_RECOMBINATION: Random ES distribution      
 Gene set null distribution of ES for **KEGG\_HOMOLOGOUS\_RECOMBINATION**

  
