# Supplementary material for: DNA methylation-based classifier and gene expression signatures detect BRCAness in osteosarcoma
Source: PLoS Comput Biol. 2021 Nov 11;17(11):e1009562. doi: 10.1371/journal.pcbi.1009562 (PMC8584788; doi:10.1371/journal.pcbi.1009562)
Supplement: S2 File — (ZIP) [file pcbi.1009562.s002.zip › S2_File/my_analysis_Kegg.GseaPreranked.1581692187239/KEGG_INTESTINAL_IMMUNE_NETWORK_FOR_IGA_PRODUCTION.html]

Details for gene set KEGG\_INTESTINAL\_IMMUNE\_NETWORK\_FOR\_IGA\_PRODUCTION[GSEA]

|  || Dataset | DEG3\_two3dTopBottom |
| Phenotype | NoPhenotypeAvailable |
| Upregulated in class | na\_neg |
| GeneSet | KEGG\_INTESTINAL\_IMMUNE\_NETWORK\_FOR\_IGA\_PRODUCTION |
| Enrichment Score (ES) | -0.49176455 |
| Normalized Enrichment Score (NES) | -0.49176455 |
| Nominal p-value | 0.0 |
| FDR q-value | 5.479025E-4 |
| FWER p-Value | 0.0033333334 |
Table: GSEA Results Summary

  

Fig 1: Enrichment plot: KEGG\_INTESTINAL\_IMMUNE\_NETWORK\_FOR\_IGA\_PRODUCTION      
 Profile of the Running ES Score & Positions of GeneSet Members on the Rank Ordered List

  

| PROBE | GENE SYMBOL | GENE\_TITLE | RANK IN GENE LIST | RANK METRIC SCORE | RUNNING ES | CORE ENRICHMENT || 1 | TGFB1 |  |  | 760 | 88.870 | -0.0152 | No |
| 2 | CCL28 |  |  | 969 | 56.170 | -0.0024 | No |
| 3 | TNFRSF13C |  |  | 2609 | 11.680 | -0.0620 | No |
| 4 | CCL25 |  |  | 6865 | 2.497 | -0.2538 | No |
| 5 | CCR10 |  |  | 7368 | 2.210 | -0.2559 | No |
| 6 | CCL27 |  |  | 8852 | 1.603 | -0.3076 | No |
| 7 | ITGA4 |  |  | 9100 | 1.522 | -0.2969 | No |
| 8 | ICOSLG |  |  | 9649 | 1.374 | -0.3013 | No |
| 9 | TNFRSF13B |  |  | 10983 | 1.103 | -0.3454 | No |
| 10 | MAP3K14 |  |  | 11726 | -1.023 | -0.3597 | No |
| 11 | MADCAM1 |  |  | 14340 | -1.893 | -0.4685 | Yes |
| 12 | CXCR4 |  |  | 14380 | -1.918 | -0.4472 | Yes |
| 13 | CD86 |  |  | 14450 | -1.967 | -0.4275 | Yes |
| 14 | IL2 |  |  | 14536 | -2.032 | -0.4085 | Yes |
| 15 | ITGB7 |  |  | 14706 | -2.153 | -0.3938 | Yes |
| 16 | IL6 |  |  | 14833 | -2.246 | -0.3769 | Yes |
| 17 | CXCL12 |  |  | 16394 | -5.375 | -0.4325 | Yes |
| 18 | CD28 |  |  | 16514 | -5.798 | -0.4153 | Yes |
| 19 | CD40 |  |  | 17248 | -11.890 | -0.4290 | Yes |
| 20 | CCR9 |  |  | 17358 | -13.840 | -0.4113 | Yes |
| 21 | AICDA |  |  | 17683 | -21.600 | -0.4044 | Yes |
| 22 | HLA-DQA2 |  |  | 17900 | -31.160 | -0.3921 | Yes |
| 23 | HLA-DPB1 |  |  | 18263 | -63.760 | -0.3871 | Yes |
| 24 | HLA-DPA1 |  |  | 18376 | -82.260 | -0.3695 | Yes |
| 25 | HLA-DMA |  |  | 18388 | -85.320 | -0.3468 | Yes |
| 26 | HLA-DRB1 |  |  | 18392 | -86.860 | -0.3237 | Yes |
| 27 | HLA-DOA |  |  | 18426 | -97.930 | -0.3021 | Yes |
| 28 | HLA-DMB |  |  | 18587 | -159.700 | -0.2870 | Yes |
| 29 | TNFSF13B |  |  | 18668 | -201.700 | -0.2678 | Yes |
| 30 | TNFRSF17 |  |  | 18699 | -219.900 | -0.2460 | Yes |
| 31 | HLA-DRA |  |  | 18720 | -232.200 | -0.2238 | Yes |
| 32 | IL10 |  |  | 18725 | -237.700 | -0.2007 | Yes |
| 33 | HLA-DRB5 |  |  | 18733 | -243.500 | -0.1778 | Yes |
| 34 | HLA-DOB |  |  | 18766 | -268.900 | -0.1562 | Yes |
| 35 | CD80 |  |  | 18879 | -411.400 | -0.1386 | Yes |
| 36 | HLA-DQA1 |  |  | 18983 | -673.000 | -0.1205 | Yes |
| 37 | HLA-DQB1 |  |  | 19125 | -1584.000 | -0.1044 | Yes |
| 38 | ICOS |  |  | 19287 | -4615.000 | -0.0893 | Yes |
| 39 | PIGR |  |  | 19308 | -5489.000 | -0.0670 | Yes |
| 40 | TNFSF13 |  |  | 19398 | -13390.000 | -0.0483 | Yes |
| 41 | CD40LG |  |  | 19510 | -66050.000 | -0.0306 | Yes |
| 42 | IL15RA |  |  | 19521 | -76760.000 | -0.0079 | Yes |
| 43 | IL15 |  |  | 19608 | -273800.000 | 0.0110 | Yes |
Table: GSEA details [plain text format]

  

Fig 2: KEGG\_INTESTINAL\_IMMUNE\_NETWORK\_FOR\_IGA\_PRODUCTION: Random ES distribution      
 Gene set null distribution of ES for **KEGG\_INTESTINAL\_IMMUNE\_NETWORK\_FOR\_IGA\_PRODUCTION**

  
