# Supplementary material for: DNA methylation-based classifier and gene expression signatures detect BRCAness in osteosarcoma
Source: PLoS Comput Biol. 2021 Nov 11;17(11):e1009562. doi: 10.1371/journal.pcbi.1009562 (PMC8584788; doi:10.1371/journal.pcbi.1009562)
Supplement: S2 File — (ZIP) [file pcbi.1009562.s002.zip › S2_File/my_analysis_Kegg.GseaPreranked.1581692187239/KEGG_LEISHMANIA_INFECTION.html]

Details for gene set KEGG\_LEISHMANIA\_INFECTION[GSEA]

|  || Dataset | DEG3\_two3dTopBottom |
| Phenotype | NoPhenotypeAvailable |
| Upregulated in class | na\_neg |
| GeneSet | KEGG\_LEISHMANIA\_INFECTION |
| Enrichment Score (ES) | -0.42677042 |
| Normalized Enrichment Score (NES) | -0.42677042 |
| Nominal p-value | 0.0 |
| FDR q-value | 0.0023021852 |
| FWER p-Value | 0.022666667 |
Table: GSEA Results Summary

  

Fig 1: Enrichment plot: KEGG\_LEISHMANIA\_INFECTION      
 Profile of the Running ES Score & Positions of GeneSet Members on the Rank Ordered List

  

| PROBE | GENE SYMBOL | GENE\_TITLE | RANK IN GENE LIST | RANK METRIC SCORE | RUNNING ES | CORE ENRICHMENT || 1 | TGFB1 |  |  | 760 | 88.870 | -0.0240 | No |
| 2 | ELK1 |  |  | 1622 | 24.210 | -0.0531 | No |
| 3 | RELA |  |  | 5570 | 3.508 | -0.2383 | No |
| 4 | NFKBIB |  |  | 5807 | 3.278 | -0.2358 | No |
| 5 | MAPK14 |  |  | 6175 | 2.949 | -0.2399 | No |
| 6 | IRAK1 |  |  | 6235 | 2.911 | -0.2284 | No |
| 7 | TAB1 |  |  | 6845 | 2.510 | -0.2447 | No |
| 8 | MARCKSL1 |  |  | 9008 | 1.551 | -0.3396 | No |
| 9 | ITGA4 |  |  | 9100 | 1.522 | -0.3297 | No |
| 10 | TGFB3 |  |  | 9199 | 1.494 | -0.3202 | No |
| 11 | TRAF6 |  |  | 9696 | 1.361 | -0.3308 | No |
| 12 | ITGB1 |  |  | 10236 | 1.237 | -0.3436 | No |
| 13 | MAPK12 |  |  | 10946 | 1.108 | -0.3650 | No |
| 14 | MAPK3 |  |  | 11618 | -1.006 | -0.3845 | No |
| 15 | IRAK4 |  |  | 12455 | -1.162 | -0.4123 | Yes |
| 16 | IFNGR2 |  |  | 12521 | -1.174 | -0.4011 | Yes |
| 17 | TAB2 |  |  | 12688 | -1.209 | -0.3950 | Yes |
| 18 | MAPK1 |  |  | 12827 | -1.240 | -0.3875 | Yes |
| 19 | NOS2 |  |  | 12925 | -1.266 | -0.3779 | Yes |
| 20 | STAT1 |  |  | 13051 | -1.305 | -0.3697 | Yes |
| 21 | FCGR3B |  |  | 13463 | -1.443 | -0.3760 | Yes |
| 22 | TGFB2 |  |  | 13716 | -1.553 | -0.3743 | Yes |
| 23 | FCGR2C |  |  | 14036 | -1.707 | -0.3759 | Yes |
| 24 | JAK1 |  |  | 14168 | -1.777 | -0.3681 | Yes |
| 25 | MAP3K7 |  |  | 14192 | -1.787 | -0.3548 | Yes |
| 26 | IL1B |  |  | 14481 | -1.989 | -0.3548 | Yes |
| 27 | FCGR2A |  |  | 14486 | -1.995 | -0.3406 | Yes |
| 28 | NCF1 |  |  | 15285 | -2.778 | -0.3664 | Yes |
| 29 | JUN |  |  | 15436 | -3.002 | -0.3595 | Yes |
| 30 | IL12A |  |  | 15711 | -3.497 | -0.3589 | Yes |
| 31 | JAK2 |  |  | 15994 | -4.064 | -0.3587 | Yes |
| 32 | MYD88 |  |  | 16255 | -4.850 | -0.3574 | Yes |
| 33 | FCGR1A |  |  | 16511 | -5.783 | -0.3558 | Yes |
| 34 | ITGB2 |  |  | 16563 | -5.997 | -0.3439 | Yes |
| 35 | FCGR3A |  |  | 16643 | -6.405 | -0.3334 | Yes |
| 36 | FOS |  |  | 16712 | -6.810 | -0.3223 | Yes |
| 37 | TNF |  |  | 16858 | -7.878 | -0.3152 | Yes |
| 38 | MAPK11 |  |  | 17413 | -14.730 | -0.3287 | Yes |
| 39 | TLR2 |  |  | 17415 | -14.760 | -0.3143 | Yes |
| 40 | IFNGR1 |  |  | 17459 | -15.430 | -0.3020 | Yes |
| 41 | NCF4 |  |  | 17477 | -15.590 | -0.2883 | Yes |
| 42 | C3 |  |  | 17530 | -16.820 | -0.2765 | Yes |
| 43 | ITGAM |  |  | 17547 | -17.420 | -0.2628 | Yes |
| 44 | IL12B |  |  | 17602 | -18.740 | -0.2510 | Yes |
| 45 | CR1 |  |  | 17742 | -24.130 | -0.2436 | Yes |
| 46 | HLA-DQA2 |  |  | 17900 | -31.160 | -0.2370 | Yes |
| 47 | TLR4 |  |  | 17928 | -32.720 | -0.2239 | Yes |
| 48 | PRKCB |  |  | 18007 | -37.280 | -0.2133 | Yes |
| 49 | NFKB1 |  |  | 18187 | -53.080 | -0.2079 | Yes |
| 50 | PTGS2 |  |  | 18203 | -55.220 | -0.1942 | Yes |
| 51 | HLA-DPB1 |  |  | 18263 | -63.760 | -0.1827 | Yes |
| 52 | NFKBIA |  |  | 18315 | -71.000 | -0.1708 | Yes |
| 53 | HLA-DPA1 |  |  | 18376 | -82.260 | -0.1593 | Yes |
| 54 | HLA-DMA |  |  | 18388 | -85.320 | -0.1454 | Yes |
| 55 | HLA-DRB1 |  |  | 18392 | -86.860 | -0.1310 | Yes |
| 56 | HLA-DOA |  |  | 18426 | -97.930 | -0.1182 | Yes |
| 57 | HLA-DMB |  |  | 18587 | -159.700 | -0.1118 | Yes |
| 58 | MAPK13 |  |  | 18639 | -189.500 | -0.0999 | Yes |
| 59 | CYBA |  |  | 18692 | -216.700 | -0.0880 | Yes |
| 60 | HLA-DRA |  |  | 18720 | -232.200 | -0.0749 | Yes |
| 61 | IL10 |  |  | 18725 | -237.700 | -0.0606 | Yes |
| 62 | HLA-DRB5 |  |  | 18733 | -243.500 | -0.0465 | Yes |
| 63 | HLA-DOB |  |  | 18766 | -268.900 | -0.0336 | Yes |
| 64 | PTPN6 |  |  | 18930 | -522.600 | -0.0274 | Yes |
| 65 | HLA-DQA1 |  |  | 18983 | -673.000 | -0.0155 | Yes |
| 66 | HLA-DQB1 |  |  | 19125 | -1584.000 | -0.0082 | Yes |
| 67 | NCF2 |  |  | 19167 | -2011.000 | 0.0043 | Yes |
| 68 | IL1A |  |  | 19468 | -33230.000 | 0.0036 | Yes |
| 69 | IFNG |  |  | 19720 | -4827000.000 | 0.0054 | Yes |
Table: GSEA details [plain text format]

  

Fig 2: KEGG\_LEISHMANIA\_INFECTION: Random ES distribution      
 Gene set null distribution of ES for **KEGG\_LEISHMANIA\_INFECTION**

  
