# Supplementary material for: DNA methylation-based classifier and gene expression signatures detect BRCAness in osteosarcoma
Source: PLoS Comput Biol. 2021 Nov 11;17(11):e1009562. doi: 10.1371/journal.pcbi.1009562 (PMC8584788; doi:10.1371/journal.pcbi.1009562)
Supplement: S2 File — (ZIP) [file pcbi.1009562.s002.zip › S2_File/my_analysis_Kegg.GseaPreranked.1581692187239/KEGG_LINOLEIC_ACID_METABOLISM.html]

Details for gene set KEGG\_LINOLEIC\_ACID\_METABOLISM[GSEA]

|  || Dataset | DEG3\_two3dTopBottom |
| Phenotype | NoPhenotypeAvailable |
| Upregulated in class | na\_neg |
| GeneSet | KEGG\_LINOLEIC\_ACID\_METABOLISM |
| Enrichment Score (ES) | -0.4283698 |
| Normalized Enrichment Score (NES) | -0.4283698 |
| Nominal p-value | 6.5876154E-4 |
| FDR q-value | 0.0023498773 |
| FWER p-Value | 0.021333333 |
Table: GSEA Results Summary

  

Fig 1: Enrichment plot: KEGG\_LINOLEIC\_ACID\_METABOLISM      
 Profile of the Running ES Score & Positions of GeneSet Members on the Rank Ordered List

  

| PROBE | GENE SYMBOL | GENE\_TITLE | RANK IN GENE LIST | RANK METRIC SCORE | RUNNING ES | CORE ENRICHMENT || 1 | AKR1B10 |  |  | 3911 | 6.152 | -0.1520 | No |
| 2 | PLA2G6 |  |  | 7512 | 2.143 | -0.2883 | No |
| 3 | PLA2G12A |  |  | 8643 | 1.672 | -0.2999 | No |
| 4 | JMJD7-PLA2G4B |  |  | 10490 | 1.187 | -0.3477 | No |
| 5 | CYP3A43 |  |  | 11541 | 1.006 | -0.3553 | No |
| 6 | CYP2E1 |  |  | 12990 | -1.286 | -0.3829 | Yes |
| 7 | PLA2G2A |  |  | 13394 | -1.414 | -0.3578 | Yes |
| 8 | CYP3A4 |  |  | 13605 | -1.502 | -0.3230 | Yes |
| 9 | CYP2C19 |  |  | 14964 | -2.385 | -0.3461 | Yes |
| 10 | CYP2C9 |  |  | 15214 | -2.691 | -0.3132 | Yes |
| 11 | CYP2C8 |  |  | 16207 | -4.702 | -0.3178 | Yes |
| 12 | PLA2G4A |  |  | 16555 | -5.975 | -0.2899 | Yes |
| 13 | CYP1A2 |  |  | 16908 | -8.308 | -0.2622 | Yes |
| 14 | PLA2G10 |  |  | 18811 | -308.600 | -0.3128 | Yes |
| 15 | PLA2G1B |  |  | 18973 | -642.100 | -0.2755 | Yes |
| 16 | CYP2J2 |  |  | 19120 | -1515.000 | -0.2374 | Yes |
| 17 | PLA2G3 |  |  | 19276 | -4280.000 | -0.1998 | Yes |
| 18 | PLA2G5 |  |  | 19297 | -5061.000 | -0.1553 | Yes |
| 19 | PLA2G12B |  |  | 19456 | -27570.000 | -0.1178 | Yes |
| 20 | CYP3A5 |  |  | 19526 | -85430.000 | -0.0759 | Yes |
| 21 | CYP2C18 |  |  | 19594 | -204700.000 | -0.0338 | Yes |
| 22 | PLA2G2D |  |  | 19657 | -595100.000 | 0.0085 | Yes |
Table: GSEA details [plain text format]

  

Fig 2: KEGG\_LINOLEIC\_ACID\_METABOLISM: Random ES distribution      
 Gene set null distribution of ES for **KEGG\_LINOLEIC\_ACID\_METABOLISM**

  
