# Supplementary material for: DNA methylation-based classifier and gene expression signatures detect BRCAness in osteosarcoma
Source: PLoS Comput Biol. 2021 Nov 11;17(11):e1009562. doi: 10.1371/journal.pcbi.1009562 (PMC8584788; doi:10.1371/journal.pcbi.1009562)
Supplement: S2 File — (ZIP) [file pcbi.1009562.s002.zip › S2_File/my_analysis_Kegg.GseaPreranked.1581692187239/KEGG_LYSOSOME.html]

Details for gene set KEGG\_LYSOSOME[GSEA]

|  || Dataset | DEG3\_two3dTopBottom |
| Phenotype | NoPhenotypeAvailable |
| Upregulated in class | na\_neg |
| GeneSet | KEGG\_LYSOSOME |
| Enrichment Score (ES) | -0.34490693 |
| Normalized Enrichment Score (NES) | -0.34490693 |
| Nominal p-value | 0.0 |
| FDR q-value | 0.02043764 |
| FWER p-Value | 0.231 |
Table: GSEA Results Summary

  

Fig 1: Enrichment plot: KEGG\_LYSOSOME      
 Profile of the Running ES Score & Positions of GeneSet Members on the Rank Ordered List

  

| PROBE | GENE SYMBOL | GENE\_TITLE | RANK IN GENE LIST | RANK METRIC SCORE | RUNNING ES | CORE ENRICHMENT || 1 | PPT2 |  |  | 636 | 129.700 | -0.0234 | No |
| 2 | AP4B1 |  |  | 995 | 53.580 | -0.0327 | No |
| 3 | LAPTM4B |  |  | 1361 | 32.960 | -0.0424 | No |
| 4 | ABCB9 |  |  | 1483 | 28.290 | -0.0397 | No |
| 5 | CTSV |  |  | 1528 | 26.920 | -0.0331 | No |
| 6 | CLTCL1 |  |  | 1923 | 18.590 | -0.0442 | No |
| 7 | ABCA2 |  |  | 2019 | 17.470 | -0.0402 | No |
| 8 | AP3S2 |  |  | 2702 | 11.000 | -0.0659 | No |
| 9 | NEU1 |  |  | 4573 | 4.854 | -0.1519 | No |
| 10 | LAMP1 |  |  | 5162 | 3.995 | -0.1729 | No |
| 11 | AP4S1 |  |  | 5289 | 3.837 | -0.1704 | No |
| 12 | AP1M1 |  |  | 5573 | 3.505 | -0.1759 | No |
| 13 | CLTC |  |  | 5739 | 3.348 | -0.1755 | No |
| 14 | AP3S1 |  |  | 5850 | 3.227 | -0.1722 | No |
| 15 | GUSB |  |  | 6391 | 2.790 | -0.1907 | No |
| 16 | CD63 |  |  | 6402 | 2.778 | -0.1824 | No |
| 17 | AP3D1 |  |  | 7188 | 2.295 | -0.2134 | No |
| 18 | CTSF |  |  | 7519 | 2.138 | -0.2212 | No |
| 19 | CLTA |  |  | 7637 | 2.087 | -0.2183 | No |
| 20 | AP3B1 |  |  | 7916 | 1.964 | -0.2236 | No |
| 21 | GAA |  |  | 7955 | 1.945 | -0.2167 | No |
| 22 | AP4E1 |  |  | 8031 | 1.912 | -0.2116 | No |
| 23 | LAPTM4A |  |  | 8071 | 1.890 | -0.2047 | No |
| 24 | HEXA |  |  | 9145 | 1.511 | -0.2503 | No |
| 25 | CLTB |  |  | 10483 | 1.188 | -0.3093 | No |
| 26 | AP3M1 |  |  | 11186 | 1.067 | -0.3361 | Yes |
| 27 | GLA |  |  | 11209 | 1.062 | -0.3283 | Yes |
| 28 | IDS |  |  | 11423 | 1.024 | -0.3303 | Yes |
| 29 | CD164 |  |  | 11434 | 1.022 | -0.3219 | Yes |
| 30 | AP1S1 |  |  | 11530 | 1.007 | -0.3179 | Yes |
| 31 | IDUA |  |  | 11559 | 1.002 | -0.3105 | Yes |
| 32 | SMPD1 |  |  | 11640 | -1.010 | -0.3057 | Yes |
| 33 | AP1G1 |  |  | 11644 | -1.011 | -0.2970 | Yes |
| 34 | AP4M1 |  |  | 11662 | -1.014 | -0.2890 | Yes |
| 35 | IGF2R |  |  | 11666 | -1.014 | -0.2803 | Yes |
| 36 | SLC17A5 |  |  | 11771 | -1.031 | -0.2767 | Yes |
| 37 | CLN3 |  |  | 11776 | -1.032 | -0.2681 | Yes |
| 38 | AP1B1 |  |  | 11904 | -1.050 | -0.2657 | Yes |
| 39 | GALNS |  |  | 12035 | -1.076 | -0.2634 | Yes |
| 40 | NPC1 |  |  | 12152 | -1.097 | -0.2605 | Yes |
| 41 | AP3M2 |  |  | 12270 | -1.121 | -0.2575 | Yes |
| 42 | LAMP2 |  |  | 12331 | -1.134 | -0.2517 | Yes |
| 43 | ARSA |  |  | 12414 | -1.153 | -0.2470 | Yes |
| 44 | SORT1 |  |  | 12430 | -1.157 | -0.2390 | Yes |
| 45 | ATP6V0A2 |  |  | 12755 | -1.223 | -0.2465 | Yes |
| 46 | ARSG |  |  | 12761 | -1.224 | -0.2379 | Yes |
| 47 | SLC11A1 |  |  | 12876 | -1.252 | -0.2349 | Yes |
| 48 | ACP2 |  |  | 12886 | -1.255 | -0.2265 | Yes |
| 49 | GGA2 |  |  | 12939 | -1.272 | -0.2203 | Yes |
| 50 | GNPTG |  |  | 12950 | -1.274 | -0.2119 | Yes |
| 51 | CLN5 |  |  | 13168 | -1.343 | -0.2141 | Yes |
| 52 | ATP6V0B |  |  | 13303 | -1.383 | -0.2120 | Yes |
| 53 | ATP6V0A1 |  |  | 13396 | -1.415 | -0.2079 | Yes |
| 54 | CTSL |  |  | 13440 | -1.432 | -0.2012 | Yes |
| 55 | M6PR |  |  | 13445 | -1.437 | -0.1925 | Yes |
| 56 | MAN2B1 |  |  | 13627 | -1.514 | -0.1929 | Yes |
| 57 | ATP6AP1 |  |  | 13680 | -1.538 | -0.1867 | Yes |
| 58 | GBA |  |  | 13870 | -1.624 | -0.1874 | Yes |
| 59 | GGA1 |  |  | 13879 | -1.628 | -0.1790 | Yes |
| 60 | GGA3 |  |  | 14071 | -1.728 | -0.1798 | Yes |
| 61 | ATP6V0D1 |  |  | 14105 | -1.747 | -0.1726 | Yes |
| 62 | CTSA |  |  | 14142 | -1.767 | -0.1656 | Yes |
| 63 | TPP1 |  |  | 14639 | -2.102 | -0.1819 | Yes |
| 64 | SLC11A2 |  |  | 14818 | -2.238 | -0.1821 | Yes |
| 65 | PLA2G15 |  |  | 15111 | -2.569 | -0.1880 | Yes |
| 66 | AGA |  |  | 15117 | -2.579 | -0.1794 | Yes |
| 67 | ENTPD4 |  |  | 15137 | -2.609 | -0.1716 | Yes |
| 68 | SGSH |  |  | 15141 | -2.614 | -0.1629 | Yes |
| 69 | ACP5 |  |  | 15293 | -2.784 | -0.1617 | Yes |
| 70 | CTSC |  |  | 15368 | -2.912 | -0.1566 | Yes |
| 71 | AP1S2 |  |  | 15498 | -3.112 | -0.1543 | Yes |
| 72 | MANBA |  |  | 15552 | -3.206 | -0.1481 | Yes |
| 73 | PPT1 |  |  | 15778 | -3.616 | -0.1507 | Yes |
| 74 | ARSB |  |  | 16118 | -4.395 | -0.1590 | Yes |
| 75 | CTNS |  |  | 16239 | -4.788 | -0.1563 | Yes |
| 76 | HEXB |  |  | 16243 | -4.800 | -0.1476 | Yes |
| 77 | CTSG |  |  | 16432 | -5.489 | -0.1482 | Yes |
| 78 | GLB1 |  |  | 16580 | -6.076 | -0.1469 | Yes |
| 79 | CTSB |  |  | 16611 | -6.223 | -0.1395 | Yes |
| 80 | MFSD8 |  |  | 16758 | -7.110 | -0.1381 | Yes |
| 81 | ATP6V0C |  |  | 16829 | -7.662 | -0.1328 | Yes |
| 82 | MCOLN1 |  |  | 16866 | -7.973 | -0.1258 | Yes |
| 83 | CTSK |  |  | 16878 | -8.086 | -0.1175 | Yes |
| 84 | GALC |  |  | 16929 | -8.521 | -0.1112 | Yes |
| 85 | LGMN |  |  | 17038 | -9.523 | -0.1078 | Yes |
| 86 | SCARB2 |  |  | 17162 | -10.840 | -0.1052 | Yes |
| 87 | CTSO |  |  | 17270 | -12.290 | -0.1018 | Yes |
| 88 | ATP6V0D2 |  |  | 17318 | -13.050 | -0.0953 | Yes |
| 89 | ATP6V1H |  |  | 17327 | -13.200 | -0.0868 | Yes |
| 90 | LAPTM5 |  |  | 17400 | -14.440 | -0.0816 | Yes |
| 91 | TCIRG1 |  |  | 17490 | -15.830 | -0.0773 | Yes |
| 92 | CTSD |  |  | 17513 | -16.360 | -0.0696 | Yes |
| 93 | GNS |  |  | 17754 | -24.760 | -0.0729 | Yes |
| 94 | PSAPL1 |  |  | 17842 | -28.040 | -0.0685 | Yes |
| 95 | CTSW |  |  | 17845 | -28.120 | -0.0597 | Yes |
| 96 | CD68 |  |  | 18063 | -42.100 | -0.0619 | Yes |
| 97 | PSAP |  |  | 18312 | -70.850 | -0.0656 | Yes |
| 98 | GNPTAB |  |  | 18321 | -71.680 | -0.0572 | Yes |
| 99 | HYAL1 |  |  | 18326 | -73.320 | -0.0485 | Yes |
| 100 | GM2A |  |  | 18405 | -89.780 | -0.0436 | Yes |
| 101 | SUMF1 |  |  | 18537 | -136.400 | -0.0414 | Yes |
| 102 | AP1S3 |  |  | 18541 | -136.700 | -0.0327 | Yes |
| 103 | CTSE |  |  | 18590 | -160.900 | -0.0263 | Yes |
| 104 | ASAH1 |  |  | 18822 | -323.600 | -0.0292 | Yes |
| 105 | CTSZ |  |  | 18827 | -329.100 | -0.0205 | Yes |
| 106 | CTSH |  |  | 19006 | -771.500 | -0.0207 | Yes |
| 107 | NPC2 |  |  | 19077 | -1175.000 | -0.0154 | Yes |
| 108 | ATP6V0A4 |  |  | 19272 | -4202.000 | -0.0164 | Yes |
| 109 | LIPA |  |  | 19301 | -5223.000 | -0.0090 | Yes |
| 110 | AP1M2 |  |  | 19328 | -6388.000 | -0.0014 | Yes |
| 111 | FUCA1 |  |  | 19370 | -10050.000 | 0.0053 | Yes |
| 112 | CTSS |  |  | 19389 | -12090.000 | 0.0133 | Yes |
| 113 | LAMP3 |  |  | 19773 | -72080000.000 | 0.0027 | Yes |
Table: GSEA details [plain text format]

  

Fig 2: KEGG\_LYSOSOME: Random ES distribution      
 Gene set null distribution of ES for **KEGG\_LYSOSOME**

  
