# Supplementary material for: DNA methylation-based classifier and gene expression signatures detect BRCAness in osteosarcoma
Source: PLoS Comput Biol. 2021 Nov 11;17(11):e1009562. doi: 10.1371/journal.pcbi.1009562 (PMC8584788; doi:10.1371/journal.pcbi.1009562)
Supplement: S2 File — (ZIP) [file pcbi.1009562.s002.zip › S2_File/my_analysis_Kegg.GseaPreranked.1581692187239/KEGG_MISMATCH_REPAIR.html]

Details for gene set KEGG\_MISMATCH\_REPAIR[GSEA]

|  || Dataset | DEG3\_two3dTopBottom |
| Phenotype | NoPhenotypeAvailable |
| Upregulated in class | na\_pos |
| GeneSet | KEGG\_MISMATCH\_REPAIR |
| Enrichment Score (ES) | 0.49018386 |
| Normalized Enrichment Score (NES) | 0.49018386 |
| Nominal p-value | 0.0 |
| FDR q-value | 0.0010286451 |
| FWER p-Value | 0.003 |
Table: GSEA Results Summary

  

Fig 1: Enrichment plot: KEGG\_MISMATCH\_REPAIR      
 Profile of the Running ES Score & Positions of GeneSet Members on the Rank Ordered List

  

| PROBE | GENE SYMBOL | GENE\_TITLE | RANK IN GENE LIST | RANK METRIC SCORE | RUNNING ES | CORE ENRICHMENT || 1 | RPA3 |  |  | 240 | 880.400 | 0.0333 | Yes |
| 2 | POLD2 |  |  | 267 | 687.000 | 0.0775 | Yes |
| 3 | RFC5 |  |  | 1024 | 51.250 | 0.0848 | Yes |
| 4 | EXO1 |  |  | 1037 | 50.320 | 0.1296 | Yes |
| 5 | RFC2 |  |  | 1582 | 25.130 | 0.1476 | Yes |
| 6 | PMS2 |  |  | 1673 | 23.250 | 0.1885 | Yes |
| 7 | RFC3 |  |  | 1843 | 19.720 | 0.2254 | Yes |
| 8 | POLD1 |  |  | 2192 | 15.340 | 0.2533 | Yes |
| 9 | MSH6 |  |  | 2276 | 14.530 | 0.2946 | Yes |
| 10 | LIG1 |  |  | 2303 | 14.320 | 0.3387 | Yes |
| 11 | MSH2 |  |  | 2309 | 14.220 | 0.3839 | Yes |
| 12 | RFC4 |  |  | 2390 | 13.470 | 0.4253 | Yes |
| 13 | POLD3 |  |  | 3045 | 9.243 | 0.4378 | Yes |
| 14 | RPA2 |  |  | 3591 | 7.079 | 0.4557 | Yes |
| 15 | RFC1 |  |  | 5211 | 3.927 | 0.4194 | Yes |
| 16 | RPA4 |  |  | 5614 | 3.458 | 0.4446 | Yes |
| 17 | SSBP1 |  |  | 6396 | 2.785 | 0.4506 | Yes |
| 18 | RPA1 |  |  | 6513 | 2.702 | 0.4902 | Yes |
| 19 | MLH1 |  |  | 9790 | 1.340 | 0.3702 | No |
| 20 | MLH3 |  |  | 10580 | 1.172 | 0.3758 | No |
| 21 | POLD4 |  |  | 12325 | -1.133 | 0.3332 | No |
| 22 | MSH3 |  |  | 12786 | -1.232 | 0.3555 | No |
Table: GSEA details [plain text format]

  

Fig 2: KEGG\_MISMATCH\_REPAIR: Random ES distribution      
 Gene set null distribution of ES for **KEGG\_MISMATCH\_REPAIR**

  
