# Supplementary material for: DNA methylation-based classifier and gene expression signatures detect BRCAness in osteosarcoma
Source: PLoS Comput Biol. 2021 Nov 11;17(11):e1009562. doi: 10.1371/journal.pcbi.1009562 (PMC8584788; doi:10.1371/journal.pcbi.1009562)
Supplement: S2 File — (ZIP) [file pcbi.1009562.s002.zip › S2_File/my_analysis_Kegg.GseaPreranked.1581692187239/KEGG_NATURAL_KILLER_CELL_MEDIATED_CYTOTOXICITY.html]

Details for gene set KEGG\_NATURAL\_KILLER\_CELL\_MEDIATED\_CYTOTOXICITY[GSEA]

|  || Dataset | DEG3\_two3dTopBottom |
| Phenotype | NoPhenotypeAvailable |
| Upregulated in class | na\_neg |
| GeneSet | KEGG\_NATURAL\_KILLER\_CELL\_MEDIATED\_CYTOTOXICITY |
| Enrichment Score (ES) | -0.3263716 |
| Normalized Enrichment Score (NES) | -0.3263716 |
| Nominal p-value | 0.0 |
| FDR q-value | 0.028902514 |
| FWER p-Value | 0.36233333 |
Table: GSEA Results Summary

  

Fig 1: Enrichment plot: KEGG\_NATURAL\_KILLER\_CELL\_MEDIATED\_CYTOTOXICITY      
 Profile of the Running ES Score & Positions of GeneSet Members on the Rank Ordered List

  

| PROBE | GENE SYMBOL | GENE\_TITLE | RANK IN GENE LIST | RANK METRIC SCORE | RUNNING ES | CORE ENRICHMENT || 1 | MAP2K2 |  |  | 1619 | 24.320 | -0.0734 | No |
| 2 | HRAS |  |  | 1621 | 24.240 | -0.0646 | No |
| 3 | RAC1 |  |  | 1907 | 18.810 | -0.0703 | No |
| 4 | ARAF |  |  | 2799 | 10.480 | -0.1067 | No |
| 5 | ULBP2 |  |  | 2967 | 9.648 | -0.1064 | No |
| 6 | NRAS |  |  | 3612 | 7.021 | -0.1303 | No |
| 7 | PAK1 |  |  | 3718 | 6.676 | -0.1269 | No |
| 8 | PLCG1 |  |  | 4095 | 5.756 | -0.1372 | No |
| 9 | ULBP1 |  |  | 4247 | 5.463 | -0.1361 | No |
| 10 | NFATC4 |  |  | 4309 | 5.327 | -0.1304 | No |
| 11 | KRAS |  |  | 5214 | 3.924 | -0.1675 | No |
| 12 | KLRC2 |  |  | 5239 | 3.885 | -0.1599 | No |
| 13 | BRAF |  |  | 5255 | 3.869 | -0.1519 | No |
| 14 | RAC3 |  |  | 7184 | 2.298 | -0.2410 | No |
| 15 | RAF1 |  |  | 7586 | 2.107 | -0.2525 | No |
| 16 | MICA |  |  | 7700 | 2.060 | -0.2495 | No |
| 17 | NFAT5 |  |  | 7888 | 1.976 | -0.2502 | No |
| 18 | SOS1 |  |  | 8366 | 1.772 | -0.2656 | No |
| 19 | PIK3CA |  |  | 8407 | 1.757 | -0.2589 | No |
| 20 | MAP2K1 |  |  | 8855 | 1.602 | -0.2728 | No |
| 21 | ULBP3 |  |  | 9439 | 1.428 | -0.2936 | No |
| 22 | PRKCG |  |  | 9658 | 1.371 | -0.2959 | No |
| 23 | PTPN11 |  |  | 9667 | 1.368 | -0.2875 | No |
| 24 | PRKCA |  |  | 9801 | 1.339 | -0.2855 | No |
| 25 | KLRC3 |  |  | 9936 | 1.307 | -0.2835 | No |
| 26 | PIK3R2 |  |  | 10025 | 1.284 | -0.2792 | No |
| 27 | HLA-C |  |  | 10192 | 1.248 | -0.2789 | No |
| 28 | SOS2 |  |  | 10383 | 1.207 | -0.2797 | No |
| 29 | RAET1G |  |  | 10551 | 1.178 | -0.2794 | No |
| 30 | PPP3R1 |  |  | 10840 | 1.129 | -0.2853 | No |
| 31 | PIK3R3 |  |  | 11077 | 1.088 | -0.2885 | No |
| 32 | VAV2 |  |  | 11592 | -1.002 | -0.3058 | No |
| 33 | MAPK3 |  |  | 11618 | -1.006 | -0.2983 | No |
| 34 | MICB |  |  | 12016 | -1.073 | -0.3096 | No |
| 35 | PPP3CA |  |  | 12081 | -1.085 | -0.3041 | No |
| 36 | IFNGR2 |  |  | 12521 | -1.174 | -0.3176 | Yes |
| 37 | NFATC3 |  |  | 12542 | -1.178 | -0.3098 | Yes |
| 38 | BID |  |  | 12696 | -1.211 | -0.3088 | Yes |
| 39 | MAPK1 |  |  | 12827 | -1.240 | -0.3067 | Yes |
| 40 | FYN |  |  | 12970 | -1.280 | -0.3051 | Yes |
| 41 | NFATC2 |  |  | 13166 | -1.342 | -0.3062 | Yes |
| 42 | PIK3R1 |  |  | 13458 | -1.441 | -0.3122 | Yes |
| 43 | FCGR3B |  |  | 13463 | -1.443 | -0.3036 | Yes |
| 44 | HLA-A |  |  | 13593 | -1.494 | -0.3014 | Yes |
| 45 | RAET1L |  |  | 13628 | -1.514 | -0.2944 | Yes |
| 46 | RAET1E |  |  | 13733 | -1.563 | -0.2909 | Yes |
| 47 | CASP3 |  |  | 14090 | -1.736 | -0.3001 | Yes |
| 48 | TNFRSF10B |  |  | 14112 | -1.750 | -0.2924 | Yes |
| 49 | PPP3CB |  |  | 14523 | -2.017 | -0.3045 | Yes |
| 50 | CHP2 |  |  | 14775 | -2.208 | -0.3084 | Yes |
| 51 | HLA-B |  |  | 14983 | -2.404 | -0.3102 | Yes |
| 52 | NCR1 |  |  | 14999 | -2.415 | -0.3021 | Yes |
| 53 | SHC1 |  |  | 15185 | -2.661 | -0.3028 | Yes |
| 54 | IFNB1 |  |  | 15262 | -2.754 | -0.2978 | Yes |
| 55 | VAV3 |  |  | 15578 | -3.241 | -0.3050 | Yes |
| 56 | ICAM2 |  |  | 15583 | -3.249 | -0.2965 | Yes |
| 57 | PPP3R2 |  |  | 15632 | -3.339 | -0.2901 | Yes |
| 58 | FCER1G |  |  | 15683 | -3.439 | -0.2839 | Yes |
| 59 | TYROBP |  |  | 15723 | -3.522 | -0.2771 | Yes |
| 60 | PLCG2 |  |  | 16000 | -4.087 | -0.2823 | Yes |
| 61 | CD244 |  |  | 16229 | -4.768 | -0.2851 | Yes |
| 62 | PPP3CC |  |  | 16519 | -5.820 | -0.2910 | Yes |
| 63 | ITGB2 |  |  | 16563 | -5.997 | -0.2844 | Yes |
| 64 | GRB2 |  |  | 16588 | -6.110 | -0.2769 | Yes |
| 65 | FCGR3A |  |  | 16643 | -6.405 | -0.2708 | Yes |
| 66 | HLA-G |  |  | 16786 | -7.343 | -0.2693 | Yes |
| 67 | TNF |  |  | 16858 | -7.878 | -0.2641 | Yes |
| 68 | SHC3 |  |  | 16943 | -8.616 | -0.2596 | Yes |
| 69 | SHC2 |  |  | 16953 | -8.741 | -0.2513 | Yes |
| 70 | PIK3CB |  |  | 17099 | -10.230 | -0.2499 | Yes |
| 71 | SHC4 |  |  | 17342 | -13.480 | -0.2534 | Yes |
| 72 | IFNGR1 |  |  | 17459 | -15.430 | -0.2505 | Yes |
| 73 | TNFRSF10D |  |  | 17472 | -15.530 | -0.2423 | Yes |
| 74 | PTK2B |  |  | 17554 | -17.630 | -0.2377 | Yes |
| 75 | LCP2 |  |  | 17723 | -23.420 | -0.2374 | Yes |
| 76 | KIR2DL3 |  |  | 17725 | -23.460 | -0.2287 | Yes |
| 77 | GZMB |  |  | 17776 | -25.560 | -0.2224 | Yes |
| 78 | NFATC1 |  |  | 17912 | -31.830 | -0.2205 | Yes |
| 79 | SYK |  |  | 17969 | -35.080 | -0.2146 | Yes |
| 80 | VAV1 |  |  | 17990 | -36.360 | -0.2068 | Yes |
| 81 | PRKCB |  |  | 18007 | -37.280 | -0.1989 | Yes |
| 82 | SH3BP2 |  |  | 18008 | -37.300 | -0.1901 | Yes |
| 83 | FAS |  |  | 18057 | -41.750 | -0.1838 | Yes |
| 84 | PIK3R5 |  |  | 18061 | -41.890 | -0.1751 | Yes |
| 85 | KIR3DL1 |  |  | 18098 | -44.440 | -0.1682 | Yes |
| 86 | HCST |  |  | 18122 | -46.900 | -0.1606 | Yes |
| 87 | TNFRSF10C |  |  | 18147 | -49.620 | -0.1530 | Yes |
| 88 | CHP1 |  |  | 18192 | -53.600 | -0.1465 | Yes |
| 89 | TNFRSF10A |  |  | 18251 | -61.870 | -0.1407 | Yes |
| 90 | PIK3CG |  |  | 18519 | -128.200 | -0.1454 | Yes |
| 91 | LAT |  |  | 18535 | -135.200 | -0.1374 | Yes |
| 92 | HLA-E |  |  | 18671 | -203.600 | -0.1355 | Yes |
| 93 | RAC2 |  |  | 18681 | -210.600 | -0.1272 | Yes |
| 94 | KIR3DL2 |  |  | 18738 | -246.800 | -0.1213 | Yes |
| 95 | KIR2DS4 |  |  | 18884 | -424.000 | -0.1198 | Yes |
| 96 | PTPN6 |  |  | 18930 | -522.600 | -0.1134 | Yes |
| 97 | KLRK1 |  |  | 19197 | -2396.000 | -0.1181 | Yes |
| 98 | PIK3CD |  |  | 19210 | -2582.000 | -0.1099 | Yes |
| 99 | CSF2 |  |  | 19263 | -3911.000 | -0.1038 | Yes |
| 100 | FASLG |  |  | 19371 | -10130.000 | -0.1004 | Yes |
| 101 | KLRC1 |  |  | 19464 | -31380.000 | -0.0963 | Yes |
| 102 | PRF1 |  |  | 19503 | -62340.000 | -0.0895 | Yes |
| 103 | TNFSF10 |  |  | 19548 | -109200.000 | -0.0829 | Yes |
| 104 | ITGAL |  |  | 19648 | -514800.000 | -0.0792 | Yes |
| 105 | SH2D1A |  |  | 19669 | -868100.000 | -0.0714 | Yes |
| 106 | ZAP70 |  |  | 19670 | -878600.000 | -0.0627 | Yes |
| 107 | IFNG |  |  | 19720 | -4827000.000 | -0.0564 | Yes |
| 108 | LCK |  |  | 19735 | -8978000.000 | -0.0483 | Yes |
| 109 | SH2D1B |  |  | 19756 | -21410000.000 | -0.0406 | Yes |
| 110 | NCR3 |  |  | 19757 | -21490000.000 | -0.0318 | Yes |
| 111 | CD48 |  |  | 19758 | -25800000.000 | -0.0230 | Yes |
| 112 | ICAM1 |  |  | 19781 | -101300000.000 | -0.0154 | Yes |
| 113 | KLRD1 |  |  | 19787 | -179400000.000 | -0.0068 | Yes |
| 114 | CD247 |  |  | 19811 | -17050000384.000 | 0.0008 | Yes |
Table: GSEA details [plain text format]

  

Fig 2: KEGG\_NATURAL\_KILLER\_CELL\_MEDIATED\_CYTOTOXICITY: Random ES distribution      
 Gene set null distribution of ES for **KEGG\_NATURAL\_KILLER\_CELL\_MEDIATED\_CYTOTOXICITY**

  
