# Supplementary material for: DNA methylation-based classifier and gene expression signatures detect BRCAness in osteosarcoma
Source: PLoS Comput Biol. 2021 Nov 11;17(11):e1009562. doi: 10.1371/journal.pcbi.1009562 (PMC8584788; doi:10.1371/journal.pcbi.1009562)
Supplement: S2 File — (ZIP) [file pcbi.1009562.s002.zip › S2_File/my_analysis_Kegg.GseaPreranked.1581692187239/KEGG_NUCLEOTIDE_EXCISION_REPAIR.html]

Details for gene set KEGG\_NUCLEOTIDE\_EXCISION\_REPAIR[GSEA]

|  || Dataset | DEG3\_two3dTopBottom |
| Phenotype | NoPhenotypeAvailable |
| Upregulated in class | na\_pos |
| GeneSet | KEGG\_NUCLEOTIDE\_EXCISION\_REPAIR |
| Enrichment Score (ES) | 0.38489836 |
| Normalized Enrichment Score (NES) | 0.38489836 |
| Nominal p-value | 0.0 |
| FDR q-value | 0.009075533 |
| FWER p-Value | 0.07833333 |
Table: GSEA Results Summary

  

Fig 1: Enrichment plot: KEGG\_NUCLEOTIDE\_EXCISION\_REPAIR      
 Profile of the Running ES Score & Positions of GeneSet Members on the Rank Ordered List

  

| PROBE | GENE SYMBOL | GENE\_TITLE | RANK IN GENE LIST | RANK METRIC SCORE | RUNNING ES | CORE ENRICHMENT || 1 | ERCC1 |  |  | 72 | 9850.000 | 0.0202 | Yes |
| 2 | RPA3 |  |  | 240 | 880.400 | 0.0355 | Yes |
| 3 | POLD2 |  |  | 267 | 687.000 | 0.0580 | Yes |
| 4 | ERCC2 |  |  | 708 | 103.100 | 0.0596 | Yes |
| 5 | POLE2 |  |  | 914 | 62.780 | 0.0731 | Yes |
| 6 | RFC5 |  |  | 1024 | 51.250 | 0.0914 | Yes |
| 7 | RFC2 |  |  | 1582 | 25.130 | 0.0870 | Yes |
| 8 | RFC3 |  |  | 1843 | 19.720 | 0.0977 | Yes |
| 9 | POLD1 |  |  | 2192 | 15.340 | 0.1039 | Yes |
| 10 | POLE |  |  | 2249 | 14.810 | 0.1249 | Yes |
| 11 | LIG1 |  |  | 2303 | 14.320 | 0.1460 | Yes |
| 12 | RFC4 |  |  | 2390 | 13.470 | 0.1655 | Yes |
| 13 | POLD3 |  |  | 3045 | 9.243 | 0.1562 | Yes |
| 14 | ERCC4 |  |  | 3177 | 8.608 | 0.1734 | Yes |
| 15 | RPA2 |  |  | 3591 | 7.079 | 0.1763 | Yes |
| 16 | DDB1 |  |  | 3721 | 6.670 | 0.1936 | Yes |
| 17 | CUL4A |  |  | 3760 | 6.569 | 0.2155 | Yes |
| 18 | POLE4 |  |  | 3842 | 6.309 | 0.2352 | Yes |
| 19 | GTF2H3 |  |  | 4447 | 5.072 | 0.2285 | Yes |
| 20 | RAD23A |  |  | 4680 | 4.676 | 0.2406 | Yes |
| 21 | POLE3 |  |  | 4864 | 4.371 | 0.2552 | Yes |
| 22 | CETN2 |  |  | 5074 | 4.115 | 0.2684 | Yes |
| 23 | RFC1 |  |  | 5211 | 3.927 | 0.2853 | Yes |
| 24 | GTF2H4 |  |  | 5247 | 3.878 | 0.3074 | Yes |
| 25 | ERCC3 |  |  | 5486 | 3.594 | 0.3192 | Yes |
| 26 | CUL4B |  |  | 5599 | 3.471 | 0.3373 | Yes |
| 27 | RPA4 |  |  | 5614 | 3.458 | 0.3604 | Yes |
| 28 | RPA1 |  |  | 6513 | 2.702 | 0.3388 | Yes |
| 29 | GTF2H5 |  |  | 6653 | 2.623 | 0.3556 | Yes |
| 30 | ERCC8 |  |  | 6959 | 2.437 | 0.3640 | Yes |
| 31 | GTF2H2 |  |  | 7018 | 2.398 | 0.3849 | Yes |
| 32 | CCNH |  |  | 7810 | 2.012 | 0.3687 | No |
| 33 | GTF2H1 |  |  | 8216 | 1.825 | 0.3721 | No |
| 34 | RBX1 |  |  | 8857 | 1.602 | 0.3635 | No |
| 35 | XPC |  |  | 8979 | 1.559 | 0.3812 | No |
| 36 | ERCC5 |  |  | 10264 | 1.232 | 0.3401 | No |
| 37 | RAD23B |  |  | 11067 | 1.090 | 0.3234 | No |
| 38 | XPA |  |  | 11378 | 1.031 | 0.3315 | No |
| 39 | CDK7 |  |  | 11645 | -1.011 | 0.3419 | No |
| 40 | POLD4 |  |  | 12325 | -1.133 | 0.3314 | No |
| 41 | DDB2 |  |  | 12329 | -1.134 | 0.3551 | No |
| 42 | ERCC6 |  |  | 15496 | -3.109 | 0.2189 | No |
Table: GSEA details [plain text format]

  

Fig 2: KEGG\_NUCLEOTIDE\_EXCISION\_REPAIR: Random ES distribution      
 Gene set null distribution of ES for **KEGG\_NUCLEOTIDE\_EXCISION\_REPAIR**

  
