# Supplementary material for: DNA methylation-based classifier and gene expression signatures detect BRCAness in osteosarcoma
Source: PLoS Comput Biol. 2021 Nov 11;17(11):e1009562. doi: 10.1371/journal.pcbi.1009562 (PMC8584788; doi:10.1371/journal.pcbi.1009562)
Supplement: S2 File — (ZIP) [file pcbi.1009562.s002.zip › S2_File/my_analysis_Kegg.GseaPreranked.1581692187239/KEGG_N_GLYCAN_BIOSYNTHESIS.html]

Details for gene set KEGG\_N\_GLYCAN\_BIOSYNTHESIS[GSEA]

|  || Dataset | DEG3\_two3dTopBottom |
| Phenotype | NoPhenotypeAvailable |
| Upregulated in class | na\_pos |
| GeneSet | KEGG\_N\_GLYCAN\_BIOSYNTHESIS |
| Enrichment Score (ES) | 0.30021232 |
| Normalized Enrichment Score (NES) | 0.30021232 |
| Nominal p-value | 0.0 |
| FDR q-value | 0.03755014 |
| FWER p-Value | 0.56133336 |
Table: GSEA Results Summary

  

Fig 1: Enrichment plot: KEGG\_N\_GLYCAN\_BIOSYNTHESIS      
 Profile of the Running ES Score & Positions of GeneSet Members on the Rank Ordered List

  

| PROBE | GENE SYMBOL | GENE\_TITLE | RANK IN GENE LIST | RANK METRIC SCORE | RUNNING ES | CORE ENRICHMENT || 1 | ALG10 |  |  | 281 | 621.900 | 0.0080 | Yes |
| 2 | RPN2 |  |  | 746 | 94.120 | 0.0068 | Yes |
| 3 | MGAT5B |  |  | 926 | 61.520 | 0.0200 | Yes |
| 4 | ALG14 |  |  | 1214 | 40.000 | 0.0277 | Yes |
| 5 | B4GALT2 |  |  | 1669 | 23.350 | 0.0269 | Yes |
| 6 | ALG10B |  |  | 1812 | 20.310 | 0.0420 | Yes |
| 7 | DAD1 |  |  | 2004 | 17.600 | 0.0546 | Yes |
| 8 | MAN1B1 |  |  | 2200 | 15.250 | 0.0669 | Yes |
| 9 | B4GALT3 |  |  | 2380 | 13.520 | 0.0801 | Yes |
| 10 | ALG8 |  |  | 2572 | 11.880 | 0.0927 | Yes |
| 11 | MOGS |  |  | 2620 | 11.570 | 0.1125 | Yes |
| 12 | STT3A |  |  | 2712 | 10.940 | 0.1301 | Yes |
| 13 | DOLPP1 |  |  | 3282 | 8.079 | 0.1236 | Yes |
| 14 | ALG9 |  |  | 3288 | 8.062 | 0.1456 | Yes |
| 15 | ALG6 |  |  | 3459 | 7.439 | 0.1592 | Yes |
| 16 | DPM2 |  |  | 3744 | 6.599 | 0.1671 | Yes |
| 17 | MAN2A2 |  |  | 4739 | 4.581 | 0.1390 | Yes |
| 18 | ALG12 |  |  | 5179 | 3.968 | 0.1391 | Yes |
| 19 | DPM1 |  |  | 5569 | 3.508 | 0.1416 | Yes |
| 20 | DDOST |  |  | 5659 | 3.418 | 0.1593 | Yes |
| 21 | RFT1 |  |  | 5839 | 3.246 | 0.1725 | Yes |
| 22 | DPM3 |  |  | 6371 | 2.805 | 0.1679 | Yes |
| 23 | MGAT2 |  |  | 6410 | 2.774 | 0.1882 | Yes |
| 24 | MAN1A2 |  |  | 6667 | 2.616 | 0.1975 | Yes |
| 25 | DPAGT1 |  |  | 7007 | 2.407 | 0.2026 | Yes |
| 26 | MGAT5 |  |  | 7691 | 2.065 | 0.1903 | Yes |
| 27 | ALG3 |  |  | 8308 | 1.789 | 0.1813 | Yes |
| 28 | ALG13 |  |  | 8458 | 1.740 | 0.1960 | Yes |
| 29 | ALG11 |  |  | 8614 | 1.682 | 0.2104 | Yes |
| 30 | RPN1 |  |  | 8963 | 1.566 | 0.2150 | Yes |
| 31 | TUSC3 |  |  | 9585 | 1.388 | 0.2059 | Yes |
| 32 | ALG2 |  |  | 9632 | 1.377 | 0.2258 | Yes |
| 33 | ALG5 |  |  | 9901 | 1.314 | 0.2344 | Yes |
| 34 | B4GALT1 |  |  | 9948 | 1.303 | 0.2543 | Yes |
| 35 | ALG1 |  |  | 10555 | 1.176 | 0.2459 | Yes |
| 36 | STT3B |  |  | 10673 | 1.158 | 0.2622 | Yes |
| 37 | FUT8 |  |  | 10802 | 1.135 | 0.2780 | Yes |
| 38 | MGAT1 |  |  | 10803 | 1.135 | 0.3002 | Yes |
| 39 | MAN1A1 |  |  | 14288 | -1.859 | 0.1463 | No |
| 40 | MGAT4A |  |  | 14554 | -2.043 | 0.1551 | No |
| 41 | MAN2A1 |  |  | 14756 | -2.193 | 0.1672 | No |
| 42 | MGAT4B |  |  | 14793 | -2.217 | 0.1876 | No |
| 43 | MAN1C1 |  |  | 15792 | -3.644 | 0.1594 | No |
| 44 | ST6GAL1 |  |  | 18112 | -45.720 | 0.0644 | No |
| 45 | MGAT3 |  |  | 18972 | -641.100 | 0.0432 | No |
Table: GSEA details [plain text format]

  

Fig 2: KEGG\_N\_GLYCAN\_BIOSYNTHESIS: Random ES distribution      
 Gene set null distribution of ES for **KEGG\_N\_GLYCAN\_BIOSYNTHESIS**

  
