# Supplementary material for: DNA methylation-based classifier and gene expression signatures detect BRCAness in osteosarcoma
Source: PLoS Comput Biol. 2021 Nov 11;17(11):e1009562. doi: 10.1371/journal.pcbi.1009562 (PMC8584788; doi:10.1371/journal.pcbi.1009562)
Supplement: S2 File — (ZIP) [file pcbi.1009562.s002.zip › S2_File/my_analysis_Kegg.GseaPreranked.1581692187239/KEGG_ONE_CARBON_POOL_BY_FOLATE.html]

Details for gene set KEGG\_ONE\_CARBON\_POOL\_BY\_FOLATE[GSEA]

|  || Dataset | DEG3\_two3dTopBottom |
| Phenotype | NoPhenotypeAvailable |
| Upregulated in class | na\_pos |
| GeneSet | KEGG\_ONE\_CARBON\_POOL\_BY\_FOLATE |
| Enrichment Score (ES) | 0.4269917 |
| Normalized Enrichment Score (NES) | 0.4269917 |
| Nominal p-value | 6.702413E-4 |
| FDR q-value | 0.0039384644 |
| FWER p-Value | 0.023 |
Table: GSEA Results Summary

  

Fig 1: Enrichment plot: KEGG\_ONE\_CARBON\_POOL\_BY\_FOLATE      
 Profile of the Running ES Score & Positions of GeneSet Members on the Rank Ordered List

  

| PROBE | GENE SYMBOL | GENE\_TITLE | RANK IN GENE LIST | RANK METRIC SCORE | RUNNING ES | CORE ENRICHMENT || 1 | SHMT2 |  |  | 18 | 446500.000 | 0.0579 | Yes |
| 2 | SHMT1 |  |  | 896 | 65.080 | 0.0725 | Yes |
| 3 | DHFR |  |  | 1263 | 37.370 | 0.1128 | Yes |
| 4 | GART |  |  | 1636 | 23.930 | 0.1529 | Yes |
| 5 | TYMS |  |  | 2217 | 15.100 | 0.1824 | Yes |
| 6 | MTHFD2 |  |  | 3055 | 9.186 | 0.1990 | Yes |
| 7 | MTFMT |  |  | 4525 | 4.939 | 0.1836 | Yes |
| 8 | ALDH1L1 |  |  | 5495 | 3.587 | 0.1936 | Yes |
| 9 | ATIC |  |  | 6708 | 2.586 | 0.1912 | Yes |
| 10 | MTHFD1 |  |  | 8256 | 1.810 | 0.1719 | Yes |
| 11 | MTHFS |  |  | 8543 | 1.711 | 0.2163 | Yes |
| 12 | AMT |  |  | 8794 | 1.618 | 0.2625 | Yes |
| 13 | MTHFD1L |  |  | 8935 | 1.575 | 0.3143 | Yes |
| 14 | MTR |  |  | 9909 | 1.313 | 0.3240 | Yes |
| 15 | FTCD |  |  | 10028 | 1.283 | 0.3769 | Yes |
| 16 | MTHFD2L |  |  | 10201 | 1.246 | 0.4270 | Yes |
| 17 | MTHFR |  |  | 18345 | -75.710 | 0.0748 | No |
Table: GSEA details [plain text format]

  

Fig 2: KEGG\_ONE\_CARBON\_POOL\_BY\_FOLATE: Random ES distribution      
 Gene set null distribution of ES for **KEGG\_ONE\_CARBON\_POOL\_BY\_FOLATE**

  
