# Supplementary material for: DNA methylation-based classifier and gene expression signatures detect BRCAness in osteosarcoma
Source: PLoS Comput Biol. 2021 Nov 11;17(11):e1009562. doi: 10.1371/journal.pcbi.1009562 (PMC8584788; doi:10.1371/journal.pcbi.1009562)
Supplement: S2 File — (ZIP) [file pcbi.1009562.s002.zip › S2_File/my_analysis_Kegg.GseaPreranked.1581692187239/KEGG_PRIMARY_IMMUNODEFICIENCY.html]

Details for gene set KEGG\_PRIMARY\_IMMUNODEFICIENCY[GSEA]

|  || Dataset | DEG3\_two3dTopBottom |
| Phenotype | NoPhenotypeAvailable |
| Upregulated in class | na\_neg |
| GeneSet | KEGG\_PRIMARY\_IMMUNODEFICIENCY |
| Enrichment Score (ES) | -0.45344013 |
| Normalized Enrichment Score (NES) | -0.45344013 |
| Nominal p-value | 0.0 |
| FDR q-value | 0.0010281459 |
| FWER p-Value | 0.008666666 |
Table: GSEA Results Summary

  

Fig 1: Enrichment plot: KEGG\_PRIMARY\_IMMUNODEFICIENCY      
 Profile of the Running ES Score & Positions of GeneSet Members on the Rank Ordered List

  

| PROBE | GENE SYMBOL | GENE\_TITLE | RANK IN GENE LIST | RANK METRIC SCORE | RUNNING ES | CORE ENRICHMENT || 1 | TNFRSF13C |  |  | 2609 | 11.680 | -0.1024 | No |
| 2 | RAG2 |  |  | 5016 | 4.181 | -0.1945 | No |
| 3 | RFXANK |  |  | 5150 | 4.021 | -0.1719 | No |
| 4 | UNG |  |  | 5601 | 3.471 | -0.1652 | No |
| 5 | RAG1 |  |  | 7210 | 2.286 | -0.2170 | No |
| 6 | IKBKG |  |  | 7246 | 2.269 | -0.1894 | No |
| 7 | RFXAP |  |  | 7562 | 2.118 | -0.1759 | No |
| 8 | ADA |  |  | 10604 | 1.168 | -0.3001 | No |
| 9 | TNFRSF13B |  |  | 10983 | 1.103 | -0.2898 | No |
| 10 | DCLRE1C |  |  | 11239 | 1.057 | -0.2733 | No |
| 11 | RFX5 |  |  | 13260 | -1.366 | -0.3459 | No |
| 12 | AIRE |  |  | 14482 | -1.991 | -0.3782 | No |
| 13 | TAP1 |  |  | 14789 | -2.214 | -0.3642 | No |
| 14 | TAP2 |  |  | 15612 | -3.293 | -0.3763 | No |
| 15 | BLNK |  |  | 17139 | -10.700 | -0.4240 | Yes |
| 16 | CD40 |  |  | 17248 | -11.890 | -0.4001 | Yes |
| 17 | CD19 |  |  | 17406 | -14.570 | -0.3786 | Yes |
| 18 | AICDA |  |  | 17683 | -21.600 | -0.3631 | Yes |
| 19 | CIITA |  |  | 18136 | -48.460 | -0.3566 | Yes |
| 20 | IL7R |  |  | 18311 | -70.790 | -0.3359 | Yes |
| 21 | JAK3 |  |  | 18706 | -222.000 | -0.3264 | Yes |
| 22 | CD4 |  |  | 18780 | -283.000 | -0.3007 | Yes |
| 23 | CD8B |  |  | 18793 | -289.900 | -0.2719 | Yes |
| 24 | CD79A |  |  | 18862 | -378.400 | -0.2459 | Yes |
| 25 | BTK |  |  | 18958 | -601.300 | -0.2213 | Yes |
| 26 | ICOS |  |  | 19287 | -4615.000 | -0.2085 | Yes |
| 27 | CD3E |  |  | 19417 | -16680.000 | -0.1856 | Yes |
| 28 | CD3D |  |  | 19448 | -25370.000 | -0.1577 | Yes |
| 29 | PTPRC |  |  | 19477 | -39510.000 | -0.1297 | Yes |
| 30 | CD40LG |  |  | 19510 | -66050.000 | -0.1019 | Yes |
| 31 | CD8A |  |  | 19544 | -106100.000 | -0.0741 | Yes |
| 32 | ZAP70 |  |  | 19670 | -878600.000 | -0.0510 | Yes |
| 33 | LCK |  |  | 19735 | -8978000.000 | -0.0249 | Yes |
| 34 | IL2RG |  |  | 19824 | -5699000074240.000 | 0.0001 | Yes |
Table: GSEA details [plain text format]

  

Fig 2: KEGG\_PRIMARY\_IMMUNODEFICIENCY: Random ES distribution      
 Gene set null distribution of ES for **KEGG\_PRIMARY\_IMMUNODEFICIENCY**

  
