# Supplementary material for: DNA methylation-based classifier and gene expression signatures detect BRCAness in osteosarcoma
Source: PLoS Comput Biol. 2021 Nov 11;17(11):e1009562. doi: 10.1371/journal.pcbi.1009562 (PMC8584788; doi:10.1371/journal.pcbi.1009562)
Supplement: S2 File — (ZIP) [file pcbi.1009562.s002.zip › S2_File/my_analysis_Kegg.GseaPreranked.1581692187239/KEGG_PROTEASOME.html]

Details for gene set KEGG\_PROTEASOME[GSEA]

|  || Dataset | DEG3\_two3dTopBottom |
| Phenotype | NoPhenotypeAvailable |
| Upregulated in class | na\_pos |
| GeneSet | KEGG\_PROTEASOME |
| Enrichment Score (ES) | 0.35864076 |
| Normalized Enrichment Score (NES) | 0.35864076 |
| Nominal p-value | 0.0 |
| FDR q-value | 0.014917949 |
| FWER p-Value | 0.162 |
Table: GSEA Results Summary

  

Fig 1: Enrichment plot: KEGG\_PROTEASOME      
 Profile of the Running ES Score & Positions of GeneSet Members on the Rank Ordered List

  

| PROBE | GENE SYMBOL | GENE\_TITLE | RANK IN GENE LIST | RANK METRIC SCORE | RUNNING ES | CORE ENRICHMENT || 1 | PSMD11 |  |  | 315 | 501.900 | 0.0073 | Yes |
| 2 | PSMA2 |  |  | 456 | 246.900 | 0.0235 | Yes |
| 3 | PSMB5 |  |  | 630 | 131.600 | 0.0380 | Yes |
| 4 | PSMC4 |  |  | 880 | 66.130 | 0.0487 | Yes |
| 5 | PSMA5 |  |  | 900 | 64.530 | 0.0710 | Yes |
| 6 | PSMC3 |  |  | 928 | 60.740 | 0.0929 | Yes |
| 7 | PSMA6 |  |  | 1458 | 29.240 | 0.0894 | Yes |
| 8 | PSMD3 |  |  | 1592 | 24.910 | 0.1059 | Yes |
| 9 | PSMB3 |  |  | 1729 | 22.090 | 0.1223 | Yes |
| 10 | PSMD13 |  |  | 2441 | 13.080 | 0.1096 | Yes |
| 11 | PSME3 |  |  | 2457 | 12.960 | 0.1321 | Yes |
| 12 | PSMD12 |  |  | 3143 | 8.767 | 0.1208 | Yes |
| 13 | PSMA7 |  |  | 3328 | 7.916 | 0.1347 | Yes |
| 14 | PSME4 |  |  | 3674 | 6.812 | 0.1405 | Yes |
| 15 | PSMC1 |  |  | 3866 | 6.247 | 0.1541 | Yes |
| 16 | PSMC5 |  |  | 3892 | 6.193 | 0.1761 | Yes |
| 17 | PSMD2 |  |  | 4978 | 4.231 | 0.1445 | Yes |
| 18 | PSME2 |  |  | 5078 | 4.111 | 0.1628 | Yes |
| 19 | PSMC2 |  |  | 5081 | 4.100 | 0.1859 | Yes |
| 20 | PSMF1 |  |  | 5217 | 3.923 | 0.2024 | Yes |
| 21 | PSMD7 |  |  | 5237 | 3.886 | 0.2247 | Yes |
| 22 | PSMD14 |  |  | 5442 | 3.641 | 0.2376 | Yes |
| 23 | PSMA4 |  |  | 5488 | 3.594 | 0.2586 | Yes |
| 24 | PSMD8 |  |  | 5808 | 3.278 | 0.2657 | Yes |
| 25 | PSMB1 |  |  | 5872 | 3.209 | 0.2858 | Yes |
| 26 | PSMC6 |  |  | 6048 | 3.054 | 0.3002 | Yes |
| 27 | PSMA1 |  |  | 6618 | 2.645 | 0.2947 | Yes |
| 28 | PSMD4 |  |  | 6660 | 2.620 | 0.3159 | Yes |
| 29 | POMP |  |  | 8073 | 1.890 | 0.2678 | Yes |
| 30 | PSMD1 |  |  | 8246 | 1.814 | 0.2823 | Yes |
| 31 | PSMB7 |  |  | 8621 | 1.680 | 0.2867 | Yes |
| 32 | PSMB4 |  |  | 8646 | 1.671 | 0.3087 | Yes |
| 33 | PSMB11 |  |  | 8806 | 1.614 | 0.3240 | Yes |
| 34 | PSME1 |  |  | 8874 | 1.596 | 0.3438 | Yes |
| 35 | PSMB6 |  |  | 9098 | 1.523 | 0.3558 | Yes |
| 36 | PSMA3 |  |  | 9503 | 1.411 | 0.3586 | Yes |
| 37 | PSMB2 |  |  | 10815 | 1.133 | 0.3156 | No |
| 38 | PSMB8 |  |  | 10922 | 1.112 | 0.3335 | No |
| 39 | PSMD6 |  |  | 13114 | -1.325 | 0.2460 | No |
| 40 | PSMB9 |  |  | 15139 | -2.613 | 0.1670 | No |
| 41 | PSMA8 |  |  | 16742 | -7.005 | 0.1093 | No |
| 42 | PSMB10 |  |  | 16966 | -8.818 | 0.1213 | No |
| 43 | IFNG |  |  | 19720 | -4827000.000 | 0.0054 | No |
Table: GSEA details [plain text format]

  

Fig 2: KEGG\_PROTEASOME: Random ES distribution      
 Gene set null distribution of ES for **KEGG\_PROTEASOME**

  
