# Supplementary material for: DNA methylation-based classifier and gene expression signatures detect BRCAness in osteosarcoma
Source: PLoS Comput Biol. 2021 Nov 11;17(11):e1009562. doi: 10.1371/journal.pcbi.1009562 (PMC8584788; doi:10.1371/journal.pcbi.1009562)
Supplement: S2 File — (ZIP) [file pcbi.1009562.s002.zip › S2_File/my_analysis_Kegg.GseaPreranked.1581692187239/KEGG_PROTEIN_EXPORT.html]

Details for gene set KEGG\_PROTEIN\_EXPORT[GSEA]

|  || Dataset | DEG3\_two3dTopBottom |
| Phenotype | NoPhenotypeAvailable |
| Upregulated in class | na\_pos |
| GeneSet | KEGG\_PROTEIN\_EXPORT |
| Enrichment Score (ES) | 0.33808497 |
| Normalized Enrichment Score (NES) | 0.33808497 |
| Nominal p-value | 0.008130081 |
| FDR q-value | 0.0179484 |
| FWER p-Value | 0.269 |
Table: GSEA Results Summary

  

Fig 1: Enrichment plot: KEGG\_PROTEIN\_EXPORT      
 Profile of the Running ES Score & Positions of GeneSet Members on the Rank Ordered List

  

| PROBE | GENE SYMBOL | GENE\_TITLE | RANK IN GENE LIST | RANK METRIC SCORE | RUNNING ES | CORE ENRICHMENT || 1 | SRP54 |  |  | 235 | 923.900 | 0.0336 | Yes |
| 2 | SEC61G |  |  | 344 | 412.600 | 0.0736 | Yes |
| 3 | SRP72 |  |  | 377 | 348.200 | 0.1174 | Yes |
| 4 | IMMP1L |  |  | 830 | 75.320 | 0.1401 | Yes |
| 5 | OXA1L |  |  | 2212 | 15.140 | 0.1158 | Yes |
| 6 | SEC61A1 |  |  | 3068 | 9.097 | 0.1181 | Yes |
| 7 | SEC11A |  |  | 3668 | 6.831 | 0.1333 | Yes |
| 8 | SRPRB |  |  | 3841 | 6.311 | 0.1700 | Yes |
| 9 | SEC61A2 |  |  | 4334 | 5.281 | 0.1907 | Yes |
| 10 | SPCS2 |  |  | 5070 | 4.119 | 0.1990 | Yes |
| 11 | SRP68 |  |  | 5265 | 3.861 | 0.2347 | Yes |
| 12 | HSPA5 |  |  | 5389 | 3.722 | 0.2739 | Yes |
| 13 | SRP19 |  |  | 8137 | 1.857 | 0.1807 | Yes |
| 14 | SEC61B |  |  | 8460 | 1.740 | 0.2099 | Yes |
| 15 | SEC63 |  |  | 9539 | 1.401 | 0.2009 | Yes |
| 16 | SEC62 |  |  | 9664 | 1.369 | 0.2401 | Yes |
| 17 | SEC11C |  |  | 9972 | 1.297 | 0.2700 | Yes |
| 18 | SRP9 |  |  | 10645 | 1.163 | 0.2815 | Yes |
| 19 | IMMP2L |  |  | 11946 | -1.058 | 0.2614 | Yes |
| 20 | SRP14 |  |  | 12101 | -1.089 | 0.2990 | Yes |
| 21 | SPCS1 |  |  | 12229 | -1.112 | 0.3381 | Yes |
| 22 | SPCS3 |  |  | 15363 | -2.903 | 0.2253 | No |
Table: GSEA details [plain text format]

  

Fig 2: KEGG\_PROTEIN\_EXPORT: Random ES distribution      
 Gene set null distribution of ES for **KEGG\_PROTEIN\_EXPORT**

  
