# Supplementary material for: DNA methylation-based classifier and gene expression signatures detect BRCAness in osteosarcoma
Source: PLoS Comput Biol. 2021 Nov 11;17(11):e1009562. doi: 10.1371/journal.pcbi.1009562 (PMC8584788; doi:10.1371/journal.pcbi.1009562)
Supplement: S2 File — (ZIP) [file pcbi.1009562.s002.zip › S2_File/my_analysis_Kegg.GseaPreranked.1581692187239/KEGG_PYRIMIDINE_METABOLISM.html]

Details for gene set KEGG\_PYRIMIDINE\_METABOLISM[GSEA]

|  || Dataset | DEG3\_two3dTopBottom |
| Phenotype | NoPhenotypeAvailable |
| Upregulated in class | na\_pos |
| GeneSet | KEGG\_PYRIMIDINE\_METABOLISM |
| Enrichment Score (ES) | 0.26106143 |
| Normalized Enrichment Score (NES) | 0.26106143 |
| Nominal p-value | 0.0 |
| FDR q-value | 0.08188289 |
| FWER p-Value | 0.884 |
Table: GSEA Results Summary

  

Fig 1: Enrichment plot: KEGG\_PYRIMIDINE\_METABOLISM      
 Profile of the Running ES Score & Positions of GeneSet Members on the Rank Ordered List

  

| PROBE | GENE SYMBOL | GENE\_TITLE | RANK IN GENE LIST | RANK METRIC SCORE | RUNNING ES | CORE ENRICHMENT || 1 | NT5M |  |  | 92 | 6049.000 | 0.0056 | Yes |
| 2 | POLD2 |  |  | 267 | 687.000 | 0.0071 | Yes |
| 3 | POLR1C |  |  | 324 | 479.100 | 0.0146 | Yes |
| 4 | PRIM1 |  |  | 435 | 269.800 | 0.0193 | Yes |
| 5 | CAD |  |  | 764 | 87.990 | 0.0130 | Yes |
| 6 | POLE2 |  |  | 914 | 62.780 | 0.0158 | Yes |
| 7 | TK1 |  |  | 948 | 58.030 | 0.0244 | Yes |
| 8 | POLR2G |  |  | 1002 | 53.020 | 0.0320 | Yes |
| 9 | POLA1 |  |  | 1329 | 34.300 | 0.0258 | Yes |
| 10 | RRM1 |  |  | 1338 | 33.770 | 0.0357 | Yes |
| 11 | NT5C1A |  |  | 1342 | 33.640 | 0.0459 | Yes |
| 12 | ZNRD1 |  |  | 1521 | 27.170 | 0.0472 | Yes |
| 13 | POLR2D |  |  | 1814 | 20.310 | 0.0427 | Yes |
| 14 | UCK2 |  |  | 2030 | 17.360 | 0.0421 | Yes |
| 15 | POLR3F |  |  | 2096 | 16.490 | 0.0491 | Yes |
| 16 | UCKL1 |  |  | 2110 | 16.350 | 0.0588 | Yes |
| 17 | POLD1 |  |  | 2192 | 15.340 | 0.0650 | Yes |
| 18 | TYMS |  |  | 2217 | 15.100 | 0.0741 | Yes |
| 19 | POLE |  |  | 2249 | 14.810 | 0.0828 | Yes |
| 20 | NME4 |  |  | 2335 | 13.970 | 0.0888 | Yes |
| 21 | POLR3B |  |  | 2447 | 13.050 | 0.0935 | Yes |
| 22 | POLA2 |  |  | 2453 | 12.990 | 0.1035 | Yes |
| 23 | POLR2C |  |  | 2877 | 10.110 | 0.0924 | Yes |
| 24 | POLD3 |  |  | 3045 | 9.243 | 0.0943 | Yes |
| 25 | RRM2 |  |  | 3073 | 9.072 | 0.1032 | Yes |
| 26 | NME1-NME2 |  |  | 3312 | 7.988 | 0.1014 | Yes |
| 27 | PRIM2 |  |  | 3330 | 7.906 | 0.1109 | Yes |
| 28 | ITPA |  |  | 3350 | 7.816 | 0.1202 | Yes |
| 29 | POLR3K |  |  | 3476 | 7.396 | 0.1242 | Yes |
| 30 | POLE4 |  |  | 3842 | 6.309 | 0.1160 | Yes |
| 31 | PNPT1 |  |  | 3916 | 6.140 | 0.1226 | Yes |
| 32 | ENTPD6 |  |  | 4027 | 5.909 | 0.1274 | Yes |
| 33 | DTYMK |  |  | 4052 | 5.851 | 0.1365 | Yes |
| 34 | POLR1A |  |  | 4202 | 5.554 | 0.1392 | Yes |
| 35 | UPP1 |  |  | 4224 | 5.522 | 0.1485 | Yes |
| 36 | UCK1 |  |  | 4338 | 5.274 | 0.1530 | Yes |
| 37 | NT5C |  |  | 4376 | 5.221 | 0.1615 | Yes |
| 38 | POLR2B |  |  | 4426 | 5.113 | 0.1693 | Yes |
| 39 | NME1 |  |  | 4466 | 5.044 | 0.1776 | Yes |
| 40 | POLR3D |  |  | 4572 | 4.855 | 0.1826 | Yes |
| 41 | POLR2J |  |  | 4589 | 4.836 | 0.1921 | Yes |
| 42 | CANT1 |  |  | 4690 | 4.654 | 0.1974 | Yes |
| 43 | UPRT |  |  | 4780 | 4.518 | 0.2032 | Yes |
| 44 | POLE3 |  |  | 4864 | 4.371 | 0.2093 | Yes |
| 45 | NME7 |  |  | 5029 | 4.164 | 0.2113 | Yes |
| 46 | POLR2F |  |  | 5116 | 4.066 | 0.2172 | Yes |
| 47 | UMPS |  |  | 5230 | 3.902 | 0.2218 | Yes |
| 48 | POLR3G |  |  | 5329 | 3.791 | 0.2271 | Yes |
| 49 | POLR3H |  |  | 5362 | 3.755 | 0.2358 | Yes |
| 50 | POLR2I |  |  | 5470 | 3.612 | 0.2407 | Yes |
| 51 | CTPS2 |  |  | 5634 | 3.441 | 0.2428 | Yes |
| 52 | POLR2L |  |  | 5727 | 3.358 | 0.2484 | Yes |
| 53 | POLR1B |  |  | 6492 | 2.713 | 0.2200 | Yes |
| 54 | NT5C3A |  |  | 6600 | 2.654 | 0.2249 | Yes |
| 55 | POLR2J3 |  |  | 6611 | 2.650 | 0.2347 | Yes |
| 56 | POLR2K |  |  | 6668 | 2.616 | 0.2421 | Yes |
| 57 | DHODH |  |  | 6846 | 2.509 | 0.2435 | Yes |
| 58 | POLR2H |  |  | 7338 | 2.224 | 0.2289 | Yes |
| 59 | CTPS1 |  |  | 7430 | 2.185 | 0.2346 | Yes |
| 60 | UPP2 |  |  | 7443 | 2.179 | 0.2443 | Yes |
| 61 | POLR1E |  |  | 7500 | 2.148 | 0.2518 | Yes |
| 62 | NME2 |  |  | 7643 | 2.085 | 0.2549 | Yes |
| 63 | POLR2A |  |  | 7850 | 1.990 | 0.2548 | Yes |
| 64 | NUDT2 |  |  | 8199 | 1.831 | 0.2474 | Yes |
| 65 | DUT |  |  | 8215 | 1.825 | 0.2570 | Yes |
| 66 | CMPK1 |  |  | 8602 | 1.690 | 0.2477 | Yes |
| 67 | ENTPD8 |  |  | 8759 | 1.629 | 0.2501 | Yes |
| 68 | POLR1D |  |  | 8882 | 1.593 | 0.2542 | Yes |
| 69 | TXNRD2 |  |  | 8952 | 1.569 | 0.2611 | Yes |
| 70 | POLR2E |  |  | 9418 | 1.434 | 0.2478 | No |
| 71 | NT5C1B |  |  | 9653 | 1.372 | 0.2463 | No |
| 72 | CDA |  |  | 10136 | 1.259 | 0.2321 | No |
| 73 | POLR3A |  |  | 10569 | 1.175 | 0.2205 | No |
| 74 | CMPK2 |  |  | 11036 | 1.095 | 0.2072 | No |
| 75 | NME3 |  |  | 11678 | -1.016 | 0.1851 | No |
| 76 | POLD4 |  |  | 12325 | -1.133 | 0.1626 | No |
| 77 | DCTD |  |  | 12383 | -1.145 | 0.1700 | No |
| 78 | NME6 |  |  | 12791 | -1.233 | 0.1597 | No |
| 79 | PNP |  |  | 13308 | -1.385 | 0.1439 | No |
| 80 | ENTPD5 |  |  | 13467 | -1.444 | 0.1462 | No |
| 81 | DCK |  |  | 13732 | -1.563 | 0.1431 | No |
| 82 | AK3 |  |  | 13816 | -1.600 | 0.1492 | No |
| 83 | POLR2J2 |  |  | 13956 | -1.665 | 0.1525 | No |
| 84 | NT5E |  |  | 14040 | -1.708 | 0.1586 | No |
| 85 | NT5C2 |  |  | 14066 | -1.724 | 0.1676 | No |
| 86 | POLR3GL |  |  | 14221 | -1.809 | 0.1701 | No |
| 87 | TXNRD1 |  |  | 14832 | -2.245 | 0.1495 | No |
| 88 | ENTPD3 |  |  | 15060 | -2.502 | 0.1483 | No |
| 89 | ENTPD4 |  |  | 15137 | -2.609 | 0.1548 | No |
| 90 | TK2 |  |  | 15896 | -3.831 | 0.1267 | No |
| 91 | RRM2B |  |  | 16377 | -5.293 | 0.1127 | No |
| 92 | UPB1 |  |  | 16899 | -8.202 | 0.0966 | No |
| 93 | TYMP |  |  | 17243 | -11.800 | 0.0895 | No |
| 94 | NME5 |  |  | 17245 | -11.830 | 0.0997 | No |
| 95 | ENTPD1 |  |  | 17443 | -15.190 | 0.1001 | No |
| 96 | DPYD |  |  | 17650 | -20.260 | 0.0999 | No |
| 97 | DPYS |  |  | 19572 | -147200.000 | 0.0129 | No |
Table: GSEA details [plain text format]

  

Fig 2: KEGG\_PYRIMIDINE\_METABOLISM: Random ES distribution      
 Gene set null distribution of ES for **KEGG\_PYRIMIDINE\_METABOLISM**

  
