# Supplementary material for: DNA methylation-based classifier and gene expression signatures detect BRCAness in osteosarcoma
Source: PLoS Comput Biol. 2021 Nov 11;17(11):e1009562. doi: 10.1371/journal.pcbi.1009562 (PMC8584788; doi:10.1371/journal.pcbi.1009562)
Supplement: S2 File — (ZIP) [file pcbi.1009562.s002.zip › S2_File/my_analysis_Kegg.GseaPreranked.1581692187239/KEGG_RENIN_ANGIOTENSIN_SYSTEM.html]

Details for gene set KEGG\_RENIN\_ANGIOTENSIN\_SYSTEM[GSEA]

|  || Dataset | DEG3\_two3dTopBottom |
| Phenotype | NoPhenotypeAvailable |
| Upregulated in class | na\_neg |
| GeneSet | KEGG\_RENIN\_ANGIOTENSIN\_SYSTEM |
| Enrichment Score (ES) | -0.5471013 |
| Normalized Enrichment Score (NES) | -0.5471013 |
| Nominal p-value | 0.0 |
| FDR q-value | 0.0 |
| FWER p-Value | 0.0 |
Table: GSEA Results Summary

  

Fig 1: Enrichment plot: KEGG\_RENIN\_ANGIOTENSIN\_SYSTEM      
 Profile of the Running ES Score & Positions of GeneSet Members on the Rank Ordered List

  

| PROBE | GENE SYMBOL | GENE\_TITLE | RANK IN GENE LIST | RANK METRIC SCORE | RUNNING ES | CORE ENRICHMENT || 1 | AGT |  |  | 634 | 130.300 | 0.0305 | No |
| 2 | LNPEP |  |  | 8730 | 1.637 | -0.3156 | No |
| 3 | ENPEP |  |  | 13317 | -1.390 | -0.4846 | Yes |
| 4 | REN |  |  | 13903 | -1.641 | -0.4516 | Yes |
| 5 | CTSA |  |  | 14142 | -1.767 | -0.4011 | Yes |
| 6 | MME |  |  | 14512 | -2.011 | -0.3573 | Yes |
| 7 | NLN |  |  | 14681 | -2.135 | -0.3033 | Yes |
| 8 | CMA1 |  |  | 15416 | -2.971 | -0.2778 | Yes |
| 9 | CTSG |  |  | 16432 | -5.489 | -0.2665 | Yes |
| 10 | MAS1 |  |  | 16625 | -6.293 | -0.2137 | Yes |
| 11 | ANPEP |  |  | 17747 | -24.300 | -0.2078 | Yes |
| 12 | ACE2 |  |  | 18526 | -130.900 | -0.1846 | Yes |
| 13 | AGTR1 |  |  | 18723 | -236.100 | -0.1320 | Yes |
| 14 | AGTR2 |  |  | 19015 | -796.900 | -0.0842 | Yes |
| 15 | ACE |  |  | 19043 | -960.900 | -0.0230 | Yes |
| 16 | CPA3 |  |  | 19279 | -4322.000 | 0.0276 | Yes |
Table: GSEA details [plain text format]

  

Fig 2: KEGG\_RENIN\_ANGIOTENSIN\_SYSTEM: Random ES distribution      
 Gene set null distribution of ES for **KEGG\_RENIN\_ANGIOTENSIN\_SYSTEM**

  
