# Supplementary material for: DNA methylation-based classifier and gene expression signatures detect BRCAness in osteosarcoma
Source: PLoS Comput Biol. 2021 Nov 11;17(11):e1009562. doi: 10.1371/journal.pcbi.1009562 (PMC8584788; doi:10.1371/journal.pcbi.1009562)
Supplement: S2 File — (ZIP) [file pcbi.1009562.s002.zip › S2_File/my_analysis_Kegg.GseaPreranked.1581692187239/KEGG_RIBOSOME.html]

Details for gene set KEGG\_RIBOSOME[GSEA]

|  || Dataset | DEG3\_two3dTopBottom |
| Phenotype | NoPhenotypeAvailable |
| Upregulated in class | na\_pos |
| GeneSet | KEGG\_RIBOSOME |
| Enrichment Score (ES) | 0.3556523 |
| Normalized Enrichment Score (NES) | 0.3556523 |
| Nominal p-value | 0.0 |
| FDR q-value | 0.014659696 |
| FWER p-Value | 0.179 |
Table: GSEA Results Summary

  

Fig 1: Enrichment plot: KEGG\_RIBOSOME      
 Profile of the Running ES Score & Positions of GeneSet Members on the Rank Ordered List

  

| PROBE | GENE SYMBOL | GENE\_TITLE | RANK IN GENE LIST | RANK METRIC SCORE | RUNNING ES | CORE ENRICHMENT || 1 | RPL3L |  |  | 688 | 108.000 | -0.0229 | Yes |
| 2 | RPL10L |  |  | 833 | 75.020 | -0.0183 | Yes |
| 3 | RPL19 |  |  | 1576 | 25.370 | -0.0440 | Yes |
| 4 | RPL41 |  |  | 1625 | 24.180 | -0.0345 | Yes |
| 5 | RPL5 |  |  | 2659 | 11.330 | -0.0750 | Yes |
| 6 | RPL8 |  |  | 2678 | 11.200 | -0.0640 | Yes |
| 7 | RPL27 |  |  | 2695 | 11.070 | -0.0529 | Yes |
| 8 | RPL18 |  |  | 3024 | 9.346 | -0.0576 | Yes |
| 9 | RPS11 |  |  | 3033 | 9.311 | -0.0461 | Yes |
| 10 | RPS2 |  |  | 3056 | 9.182 | -0.0353 | Yes |
| 11 | RPL13 |  |  | 3080 | 9.058 | -0.0245 | Yes |
| 12 | RPS19 |  |  | 3347 | 7.836 | -0.0261 | Yes |
| 13 | RPS17 |  |  | 3416 | 7.576 | -0.0177 | Yes |
| 14 | RPS16 |  |  | 3455 | 7.451 | -0.0077 | Yes |
| 15 | RPL30 |  |  | 3831 | 6.332 | -0.0148 | Yes |
| 16 | RPL36A |  |  | 3879 | 6.229 | -0.0052 | Yes |
| 17 | RPL7 |  |  | 4018 | 5.930 | -0.0003 | Yes |
| 18 | RPS15A |  |  | 4067 | 5.818 | 0.0091 | Yes |
| 19 | RPL23 |  |  | 4411 | 5.154 | 0.0037 | Yes |
| 20 | RPL26L1 |  |  | 4461 | 5.053 | 0.0131 | Yes |
| 21 | RPLP2 |  |  | 4471 | 5.037 | 0.0246 | Yes |
| 22 | RPS27 |  |  | 4565 | 4.866 | 0.0317 | Yes |
| 23 | RPS4X |  |  | 4696 | 4.640 | 0.0371 | Yes |
| 24 | RPS5 |  |  | 4727 | 4.608 | 0.0475 | Yes |
| 25 | RPS10 |  |  | 5017 | 4.180 | 0.0447 | Yes |
| 26 | RPL10 |  |  | 5180 | 3.965 | 0.0484 | Yes |
| 27 | RPS21 |  |  | 5281 | 3.846 | 0.0553 | Yes |
| 28 | RPL4 |  |  | 5283 | 3.843 | 0.0671 | Yes |
| 29 | RPL37 |  |  | 5288 | 3.839 | 0.0788 | Yes |
| 30 | RPL28 |  |  | 5562 | 3.515 | 0.0769 | Yes |
| 31 | RPS9 |  |  | 5854 | 3.223 | 0.0741 | Yes |
| 32 | RPS3 |  |  | 5873 | 3.209 | 0.0851 | Yes |
| 33 | RPL18A |  |  | 5938 | 3.158 | 0.0937 | Yes |
| 34 | RPS15 |  |  | 6039 | 3.058 | 0.1006 | Yes |
| 35 | RPS7 |  |  | 6230 | 2.915 | 0.1028 | Yes |
| 36 | RPL31 |  |  | 6269 | 2.882 | 0.1128 | Yes |
| 37 | RPLP1 |  |  | 6380 | 2.799 | 0.1191 | Yes |
| 38 | RPL10A |  |  | 6565 | 2.674 | 0.1217 | Yes |
| 39 | RPL11 |  |  | 6566 | 2.674 | 0.1336 | Yes |
| 40 | RPS29 |  |  | 6760 | 2.562 | 0.1358 | Yes |
| 41 | UBA52 |  |  | 6828 | 2.520 | 0.1443 | Yes |
| 42 | RPS26 |  |  | 6847 | 2.509 | 0.1553 | Yes |
| 43 | RPL9 |  |  | 6968 | 2.435 | 0.1611 | Yes |
| 44 | RPL36AL |  |  | 6972 | 2.434 | 0.1728 | Yes |
| 45 | RPS28 |  |  | 7287 | 2.250 | 0.1689 | Yes |
| 46 | RPS8 |  |  | 7408 | 2.194 | 0.1747 | Yes |
| 47 | RPL32 |  |  | 7498 | 2.149 | 0.1821 | Yes |
| 48 | RPL12 |  |  | 7566 | 2.116 | 0.1906 | Yes |
| 49 | RPL39 |  |  | 7572 | 2.114 | 0.2022 | Yes |
| 50 | RPS13 |  |  | 7672 | 2.075 | 0.2091 | Yes |
| 51 | RPS25 |  |  | 7754 | 2.033 | 0.2169 | Yes |
| 52 | RPS27A |  |  | 7911 | 1.966 | 0.2209 | Yes |
| 53 | RPL36 |  |  | 8101 | 1.876 | 0.2233 | Yes |
| 54 | RPL7A |  |  | 8127 | 1.865 | 0.2339 | Yes |
| 55 | FAU |  |  | 8135 | 1.857 | 0.2455 | Yes |
| 56 | RPL37A |  |  | 8172 | 1.840 | 0.2555 | Yes |
| 57 | RPS23 |  |  | 8224 | 1.822 | 0.2649 | Yes |
| 58 | RPS18 |  |  | 8442 | 1.746 | 0.2658 | Yes |
| 59 | RPS20 |  |  | 8618 | 1.681 | 0.2688 | Yes |
| 60 | RPL35 |  |  | 8843 | 1.605 | 0.2694 | Yes |
| 61 | RPL34 |  |  | 8981 | 1.558 | 0.2743 | Yes |
| 62 | RPL35A |  |  | 9181 | 1.502 | 0.2762 | Yes |
| 63 | RPL27A |  |  | 9208 | 1.491 | 0.2867 | Yes |
| 64 | RPL26 |  |  | 9659 | 1.370 | 0.2759 | Yes |
| 65 | RPL22 |  |  | 9792 | 1.340 | 0.2811 | Yes |
| 66 | RPL29 |  |  | 10043 | 1.280 | 0.2803 | Yes |
| 67 | RPL3 |  |  | 10067 | 1.275 | 0.2911 | Yes |
| 68 | RPL21 |  |  | 10211 | 1.243 | 0.2957 | Yes |
| 69 | RSL24D1 |  |  | 10224 | 1.242 | 0.3070 | Yes |
| 70 | RPLP0 |  |  | 10267 | 1.232 | 0.3168 | Yes |
| 71 | RPSA |  |  | 10339 | 1.217 | 0.3251 | Yes |
| 72 | RPL15 |  |  | 10348 | 1.215 | 0.3366 | Yes |
| 73 | RPL14 |  |  | 10375 | 1.208 | 0.3472 | Yes |
| 74 | RPS6 |  |  | 10444 | 1.195 | 0.3557 | Yes |
| 75 | RPL38 |  |  | 10857 | 1.126 | 0.3467 | No |
| 76 | RPL24 |  |  | 11760 | -1.029 | 0.3129 | No |
| 77 | RPL13A |  |  | 12112 | -1.092 | 0.3070 | No |
| 78 | RPS4Y1 |  |  | 12134 | -1.094 | 0.3179 | No |
| 79 | RPS27L |  |  | 12947 | -1.273 | 0.2887 | No |
| 80 | RPS24 |  |  | 13244 | -1.362 | 0.2856 | No |
| 81 | RPL22L1 |  |  | 13556 | -1.482 | 0.2817 | No |
| 82 | RPL17 |  |  | 14166 | -1.775 | 0.2628 | No |
| 83 | RPS3A |  |  | 14563 | -2.046 | 0.2546 | No |
| 84 | RPS12 |  |  | 15347 | -2.868 | 0.2269 | No |
Table: GSEA details [plain text format]

  

Fig 2: KEGG\_RIBOSOME: Random ES distribution      
 Gene set null distribution of ES for **KEGG\_RIBOSOME**

  
