# Supplementary material for: DNA methylation-based classifier and gene expression signatures detect BRCAness in osteosarcoma
Source: PLoS Comput Biol. 2021 Nov 11;17(11):e1009562. doi: 10.1371/journal.pcbi.1009562 (PMC8584788; doi:10.1371/journal.pcbi.1009562)
Supplement: S2 File — (ZIP) [file pcbi.1009562.s002.zip › S2_File/my_analysis_Kegg.GseaPreranked.1581692187239/KEGG_RNA_DEGRADATION.html]

Details for gene set KEGG\_RNA\_DEGRADATION[GSEA]

|  || Dataset | DEG3\_two3dTopBottom |
| Phenotype | NoPhenotypeAvailable |
| Upregulated in class | na\_pos |
| GeneSet | KEGG\_RNA\_DEGRADATION |
| Enrichment Score (ES) | 0.23746452 |
| Normalized Enrichment Score (NES) | 0.23746452 |
| Nominal p-value | 0.0033647376 |
| FDR q-value | 0.12709488 |
| FWER p-Value | 0.9773333 |
Table: GSEA Results Summary

  

Fig 1: Enrichment plot: KEGG\_RNA\_DEGRADATION      
 Profile of the Running ES Score & Positions of GeneSet Members on the Rank Ordered List

  

| PROBE | GENE SYMBOL | GENE\_TITLE | RANK IN GENE LIST | RANK METRIC SCORE | RUNNING ES | CORE ENRICHMENT || 1 | CNOT3 |  |  | 11 | 2967000.000 | 0.0183 | Yes |
| 2 | ENO2 |  |  | 46 | 31810.000 | 0.0355 | Yes |
| 3 | SKIV2L |  |  | 276 | 656.500 | 0.0427 | Yes |
| 4 | LSM5 |  |  | 447 | 258.500 | 0.0530 | Yes |
| 5 | EDC3 |  |  | 864 | 69.610 | 0.0508 | Yes |
| 6 | XRN2 |  |  | 1022 | 51.480 | 0.0618 | Yes |
| 7 | ENO1 |  |  | 1251 | 37.860 | 0.0691 | Yes |
| 8 | EXOSC4 |  |  | 2271 | 14.560 | 0.0364 | Yes |
| 9 | EXOSC2 |  |  | 2412 | 13.320 | 0.0482 | Yes |
| 10 | ENO3 |  |  | 3147 | 8.743 | 0.0300 | Yes |
| 11 | LSM3 |  |  | 3170 | 8.637 | 0.0477 | Yes |
| 12 | WDR61 |  |  | 3552 | 7.198 | 0.0473 | Yes |
| 13 | DIS3 |  |  | 3615 | 7.012 | 0.0631 | Yes |
| 14 | PNPT1 |  |  | 3916 | 6.140 | 0.0668 | Yes |
| 15 | EXOSC5 |  |  | 4590 | 4.834 | 0.0516 | Yes |
| 16 | PARN |  |  | 4845 | 4.400 | 0.0576 | Yes |
| 17 | MPHOSPH6 |  |  | 4877 | 4.349 | 0.0749 | Yes |
| 18 | PAPOLG |  |  | 5024 | 4.170 | 0.0864 | Yes |
| 19 | LSM2 |  |  | 5227 | 3.903 | 0.0951 | Yes |
| 20 | EXOSC3 |  |  | 5518 | 3.563 | 0.0993 | Yes |
| 21 | DCPS |  |  | 5620 | 3.451 | 0.1130 | Yes |
| 22 | PAPOLA |  |  | 6299 | 2.858 | 0.0976 | Yes |
| 23 | EXOSC8 |  |  | 6404 | 2.777 | 0.1112 | Yes |
| 24 | LSM4 |  |  | 6456 | 2.743 | 0.1275 | Yes |
| 25 | DCP1B |  |  | 6603 | 2.653 | 0.1390 | Yes |
| 26 | HSPD1 |  |  | 6756 | 2.564 | 0.1502 | Yes |
| 27 | CNOT1 |  |  | 7390 | 2.203 | 0.1370 | Yes |
| 28 | CNOT10 |  |  | 7913 | 1.965 | 0.1295 | Yes |
| 29 | PAPOLB |  |  | 8026 | 1.914 | 0.1427 | Yes |
| 30 | DDX6 |  |  | 8978 | 1.559 | 0.1135 | Yes |
| 31 | CNOT4 |  |  | 9066 | 1.533 | 0.1279 | Yes |
| 32 | EDC4 |  |  | 9214 | 1.489 | 0.1394 | Yes |
| 33 | HSPA9 |  |  | 9656 | 1.371 | 0.1359 | Yes |
| 34 | PATL1 |  |  | 9840 | 1.328 | 0.1456 | Yes |
| 35 | ZCCHC7 |  |  | 9843 | 1.327 | 0.1643 | Yes |
| 36 | EXOSC10 |  |  | 10428 | 1.197 | 0.1537 | Yes |
| 37 | EXOSC9 |  |  | 10647 | 1.162 | 0.1615 | Yes |
| 38 | C1D |  |  | 11171 | 1.069 | 0.1539 | Yes |
| 39 | TTC37 |  |  | 11214 | 1.062 | 0.1707 | Yes |
| 40 | CNOT8 |  |  | 11449 | 1.020 | 0.1777 | Yes |
| 41 | EXOSC1 |  |  | 11664 | -1.014 | 0.1857 | Yes |
| 42 | DCP2 |  |  | 12282 | -1.123 | 0.1734 | Yes |
| 43 | LSM7 |  |  | 12357 | -1.139 | 0.1885 | Yes |
| 44 | EXOSC6 |  |  | 12454 | -1.162 | 0.2025 | Yes |
| 45 | LSM6 |  |  | 12458 | -1.163 | 0.2213 | Yes |
| 46 | EXOSC7 |  |  | 13071 | -1.312 | 0.2092 | Yes |
| 47 | CNOT6L |  |  | 13270 | -1.370 | 0.2180 | Yes |
| 48 | CNOT7 |  |  | 13341 | -1.395 | 0.2334 | Yes |
| 49 | XRN1 |  |  | 13634 | -1.516 | 0.2375 | Yes |
| 50 | LSM8 |  |  | 15008 | -2.422 | 0.1869 | No |
| 51 | LSM1 |  |  | 15080 | -2.530 | 0.2022 | No |
| 52 | DCP1A |  |  | 16241 | -4.795 | 0.1624 | No |
| 53 | CNOT6 |  |  | 16293 | -4.966 | 0.1787 | No |
Table: GSEA details [plain text format]

  

Fig 2: KEGG\_RNA\_DEGRADATION: Random ES distribution      
 Gene set null distribution of ES for **KEGG\_RNA\_DEGRADATION**

  
