# Supplementary material for: DNA methylation-based classifier and gene expression signatures detect BRCAness in osteosarcoma
Source: PLoS Comput Biol. 2021 Nov 11;17(11):e1009562. doi: 10.1371/journal.pcbi.1009562 (PMC8584788; doi:10.1371/journal.pcbi.1009562)
Supplement: S2 File — (ZIP) [file pcbi.1009562.s002.zip › S2_File/my_analysis_Kegg.GseaPreranked.1581692187239/KEGG_RNA_POLYMERASE.html]

Details for gene set KEGG\_RNA\_POLYMERASE[GSEA]

|  || Dataset | DEG3\_two3dTopBottom |
| Phenotype | NoPhenotypeAvailable |
| Upregulated in class | na\_pos |
| GeneSet | KEGG\_RNA\_POLYMERASE |
| Enrichment Score (ES) | 0.4260551 |
| Normalized Enrichment Score (NES) | 0.4260551 |
| Nominal p-value | 0.0 |
| FDR q-value | 0.0031507716 |
| FWER p-Value | 0.023 |
Table: GSEA Results Summary

  

Fig 1: Enrichment plot: KEGG\_RNA\_POLYMERASE      
 Profile of the Running ES Score & Positions of GeneSet Members on the Rank Ordered List

  

| PROBE | GENE SYMBOL | GENE\_TITLE | RANK IN GENE LIST | RANK METRIC SCORE | RUNNING ES | CORE ENRICHMENT || 1 | POLR1C |  |  | 324 | 479.100 | 0.0193 | Yes |
| 2 | POLR2G |  |  | 1002 | 53.020 | 0.0209 | Yes |
| 3 | ZNRD1 |  |  | 1521 | 27.170 | 0.0304 | Yes |
| 4 | POLR2D |  |  | 1814 | 20.310 | 0.0514 | Yes |
| 5 | POLR3F |  |  | 2096 | 16.490 | 0.0729 | Yes |
| 6 | POLR3B |  |  | 2447 | 13.050 | 0.0909 | Yes |
| 7 | POLR2C |  |  | 2877 | 10.110 | 0.1050 | Yes |
| 8 | POLR3K |  |  | 3476 | 7.396 | 0.1105 | Yes |
| 9 | POLR1A |  |  | 4202 | 5.554 | 0.1096 | Yes |
| 10 | POLR2B |  |  | 4426 | 5.113 | 0.1341 | Yes |
| 11 | POLR3D |  |  | 4572 | 4.855 | 0.1624 | Yes |
| 12 | POLR2J |  |  | 4589 | 4.836 | 0.1973 | Yes |
| 13 | POLR2F |  |  | 5116 | 4.066 | 0.2065 | Yes |
| 14 | POLR3G |  |  | 5329 | 3.791 | 0.2315 | Yes |
| 15 | POLR3H |  |  | 5362 | 3.755 | 0.2656 | Yes |
| 16 | POLR2I |  |  | 5470 | 3.612 | 0.2959 | Yes |
| 17 | POLR2L |  |  | 5727 | 3.358 | 0.3187 | Yes |
| 18 | POLR1B |  |  | 6492 | 2.713 | 0.3158 | Yes |
| 19 | POLR2J3 |  |  | 6611 | 2.650 | 0.3456 | Yes |
| 20 | POLR2K |  |  | 6668 | 2.616 | 0.3785 | Yes |
| 21 | POLR2H |  |  | 7338 | 2.224 | 0.3804 | Yes |
| 22 | POLR1E |  |  | 7500 | 2.148 | 0.4080 | Yes |
| 23 | POLR2A |  |  | 7850 | 1.990 | 0.4261 | Yes |
| 24 | POLR1D |  |  | 8882 | 1.593 | 0.4097 | No |
| 25 | POLR2E |  |  | 9418 | 1.434 | 0.4184 | No |
| 26 | POLR3A |  |  | 10569 | 1.175 | 0.3960 | No |
| 27 | POLR2J2 |  |  | 13956 | -1.665 | 0.2607 | No |
| 28 | POLR3GL |  |  | 14221 | -1.809 | 0.2831 | No |
Table: GSEA details [plain text format]

  

Fig 2: KEGG\_RNA\_POLYMERASE: Random ES distribution      
 Gene set null distribution of ES for **KEGG\_RNA\_POLYMERASE**

  
