# Supplementary material for: DNA methylation-based classifier and gene expression signatures detect BRCAness in osteosarcoma
Source: PLoS Comput Biol. 2021 Nov 11;17(11):e1009562. doi: 10.1371/journal.pcbi.1009562 (PMC8584788; doi:10.1371/journal.pcbi.1009562)
Supplement: S2 File — (ZIP) [file pcbi.1009562.s002.zip › S2_File/my_analysis_Kegg.GseaPreranked.1581692187239/KEGG_SPLICEOSOME.html]

Details for gene set KEGG\_SPLICEOSOME[GSEA]

|  || Dataset | DEG3\_two3dTopBottom |
| Phenotype | NoPhenotypeAvailable |
| Upregulated in class | na\_pos |
| GeneSet | KEGG\_SPLICEOSOME |
| Enrichment Score (ES) | 0.31089884 |
| Normalized Enrichment Score (NES) | 0.31089884 |
| Nominal p-value | 0.0 |
| FDR q-value | 0.031110238 |
| FWER p-Value | 0.473 |
Table: GSEA Results Summary

  

Fig 1: Enrichment plot: KEGG\_SPLICEOSOME      
 Profile of the Running ES Score & Positions of GeneSet Members on the Rank Ordered List

  

| PROBE | GENE SYMBOL | GENE\_TITLE | RANK IN GENE LIST | RANK METRIC SCORE | RUNNING ES | CORE ENRICHMENT || 1 | CDC5L |  |  | 74 | 9153.000 | 0.0044 | Yes |
| 2 | PPIL1 |  |  | 217 | 1071.000 | 0.0053 | Yes |
| 3 | PRPF31 |  |  | 381 | 344.500 | 0.0052 | Yes |
| 4 | SART1 |  |  | 406 | 291.000 | 0.0121 | Yes |
| 5 | LSM5 |  |  | 447 | 258.500 | 0.0182 | Yes |
| 6 | CTNNBL1 |  |  | 697 | 105.900 | 0.0137 | Yes |
| 7 | CRNKL1 |  |  | 805 | 79.570 | 0.0164 | Yes |
| 8 | BCAS2 |  |  | 867 | 68.500 | 0.0214 | Yes |
| 9 | MAGOHB |  |  | 891 | 65.360 | 0.0284 | Yes |
| 10 | EFTUD2 |  |  | 934 | 59.860 | 0.0344 | Yes |
| 11 | HNRNPM |  |  | 1192 | 41.110 | 0.0294 | Yes |
| 12 | PUF60 |  |  | 1308 | 35.300 | 0.0317 | Yes |
| 13 | DDX23 |  |  | 1390 | 31.940 | 0.0358 | Yes |
| 14 | HNRNPC |  |  | 1447 | 29.590 | 0.0410 | Yes |
| 15 | SNRPA |  |  | 1457 | 29.250 | 0.0487 | Yes |
| 16 | DHX8 |  |  | 1569 | 25.660 | 0.0512 | Yes |
| 17 | SNRNP70 |  |  | 1613 | 24.460 | 0.0572 | Yes |
| 18 | ACIN1 |  |  | 1615 | 24.380 | 0.0652 | Yes |
| 19 | SNRPB2 |  |  | 1782 | 20.830 | 0.0649 | Yes |
| 20 | SNRPD2 |  |  | 1784 | 20.810 | 0.0730 | Yes |
| 21 | HNRNPU |  |  | 1808 | 20.360 | 0.0800 | Yes |
| 22 | PRPF6 |  |  | 1887 | 19.080 | 0.0842 | Yes |
| 23 | TRA2A |  |  | 1896 | 19.010 | 0.0919 | Yes |
| 24 | U2AF1 |  |  | 1956 | 18.240 | 0.0970 | Yes |
| 25 | PRPF4 |  |  | 1982 | 17.900 | 0.1039 | Yes |
| 26 | SNRPA1 |  |  | 1987 | 17.840 | 0.1118 | Yes |
| 27 | SNRNP27 |  |  | 2053 | 17.100 | 0.1166 | Yes |
| 28 | SRSF1 |  |  | 2166 | 15.650 | 0.1191 | Yes |
| 29 | SRSF3 |  |  | 2290 | 14.460 | 0.1210 | Yes |
| 30 | PRPF38B |  |  | 2316 | 14.130 | 0.1278 | Yes |
| 31 | SRSF6 |  |  | 2387 | 13.500 | 0.1324 | Yes |
| 32 | SNRNP200 |  |  | 2434 | 13.150 | 0.1382 | Yes |
| 33 | PQBP1 |  |  | 2455 | 12.970 | 0.1453 | Yes |
| 34 | SF3A2 |  |  | 2461 | 12.930 | 0.1532 | Yes |
| 35 | SF3B2 |  |  | 2938 | 9.787 | 0.1372 | Yes |
| 36 | CHERP |  |  | 2969 | 9.643 | 0.1438 | Yes |
| 37 | PRPF19 |  |  | 2974 | 9.629 | 0.1517 | Yes |
| 38 | LSM3 |  |  | 3170 | 8.637 | 0.1499 | Yes |
| 39 | U2AF2 |  |  | 3219 | 8.386 | 0.1556 | Yes |
| 40 | SNRPD1 |  |  | 3689 | 6.771 | 0.1400 | Yes |
| 41 | SRSF10 |  |  | 3708 | 6.728 | 0.1472 | Yes |
| 42 | PCBP1 |  |  | 3820 | 6.367 | 0.1497 | Yes |
| 43 | TRA2B |  |  | 3850 | 6.298 | 0.1563 | Yes |
| 44 | SRSF7 |  |  | 3907 | 6.158 | 0.1616 | Yes |
| 45 | SNRPC |  |  | 3930 | 6.096 | 0.1686 | Yes |
| 46 | DHX16 |  |  | 4024 | 5.919 | 0.1720 | Yes |
| 47 | WBP11 |  |  | 4119 | 5.707 | 0.1754 | Yes |
| 48 | SNRPB |  |  | 4160 | 5.635 | 0.1815 | Yes |
| 49 | RBMX |  |  | 4296 | 5.359 | 0.1828 | Yes |
| 50 | THOC2 |  |  | 4436 | 5.084 | 0.1839 | Yes |
| 51 | PRPF38A |  |  | 4652 | 4.721 | 0.1811 | Yes |
| 52 | RBM22 |  |  | 4693 | 4.647 | 0.1872 | Yes |
| 53 | HNRNPA1 |  |  | 4781 | 4.514 | 0.1909 | Yes |
| 54 | THOC1 |  |  | 5136 | 4.039 | 0.1811 | Yes |
| 55 | HNRNPK |  |  | 5138 | 4.038 | 0.1891 | Yes |
| 56 | NCBP1 |  |  | 5142 | 4.036 | 0.1971 | Yes |
| 57 | PPIE |  |  | 5155 | 4.005 | 0.2046 | Yes |
| 58 | SF3B4 |  |  | 5191 | 3.957 | 0.2110 | Yes |
| 59 | LSM2 |  |  | 5227 | 3.903 | 0.2173 | Yes |
| 60 | SF3B5 |  |  | 5246 | 3.879 | 0.2246 | Yes |
| 61 | XAB2 |  |  | 5326 | 3.793 | 0.2287 | Yes |
| 62 | DDX42 |  |  | 5581 | 3.493 | 0.2239 | Yes |
| 63 | ALYREF |  |  | 5736 | 3.349 | 0.2242 | Yes |
| 64 | ISY1 |  |  | 5900 | 3.188 | 0.2241 | Yes |
| 65 | U2SURP |  |  | 5929 | 3.164 | 0.2308 | Yes |
| 66 | SF3B6 |  |  | 5983 | 3.109 | 0.2362 | Yes |
| 67 | SNRPG |  |  | 6026 | 3.066 | 0.2422 | Yes |
| 68 | TXNL4A |  |  | 6257 | 2.892 | 0.2387 | Yes |
| 69 | THOC3 |  |  | 6339 | 2.826 | 0.2427 | Yes |
| 70 | SLU7 |  |  | 6358 | 2.813 | 0.2499 | Yes |
| 71 | LSM4 |  |  | 6456 | 2.743 | 0.2531 | Yes |
| 72 | MAGOH |  |  | 6500 | 2.709 | 0.2591 | Yes |
| 73 | DHX15 |  |  | 6640 | 2.631 | 0.2602 | Yes |
| 74 | SNW1 |  |  | 6647 | 2.627 | 0.2680 | Yes |
| 75 | SYF2 |  |  | 6694 | 2.597 | 0.2738 | Yes |
| 76 | PPIH |  |  | 6754 | 2.565 | 0.2789 | Yes |
| 77 | ZMAT2 |  |  | 6781 | 2.546 | 0.2857 | Yes |
| 78 | SNRPE |  |  | 6893 | 2.481 | 0.2882 | Yes |
| 79 | PHF5A |  |  | 6906 | 2.474 | 0.2957 | Yes |
| 80 | SF3B3 |  |  | 6952 | 2.449 | 0.3016 | Yes |
| 81 | SNRNP40 |  |  | 7326 | 2.231 | 0.2908 | Yes |
| 82 | BUD31 |  |  | 7463 | 2.168 | 0.2920 | Yes |
| 83 | USP39 |  |  | 7855 | 1.989 | 0.2803 | Yes |
| 84 | PRPF18 |  |  | 7870 | 1.983 | 0.2877 | Yes |
| 85 | DDX5 |  |  | 7890 | 1.975 | 0.2949 | Yes |
| 86 | HNRNPA3 |  |  | 8039 | 1.906 | 0.2955 | Yes |
| 87 | CWC15 |  |  | 8202 | 1.829 | 0.2954 | Yes |
| 88 | SF3B1 |  |  | 8259 | 1.808 | 0.3007 | Yes |
| 89 | SF3A3 |  |  | 8426 | 1.750 | 0.3004 | Yes |
| 90 | SRSF9 |  |  | 8651 | 1.669 | 0.2972 | Yes |
| 91 | DHX38 |  |  | 8696 | 1.652 | 0.3031 | Yes |
| 92 | SNRPD3 |  |  | 8703 | 1.650 | 0.3109 | Yes |
| 93 | SF3A1 |  |  | 9241 | 1.482 | 0.2918 | No |
| 94 | TCERG1 |  |  | 9695 | 1.361 | 0.2769 | No |
| 95 | RBM25 |  |  | 10006 | 1.288 | 0.2693 | No |
| 96 | CDC40 |  |  | 10144 | 1.257 | 0.2705 | No |
| 97 | HSPA8 |  |  | 10247 | 1.235 | 0.2734 | No |
| 98 | PRPF3 |  |  | 10515 | 1.183 | 0.2680 | No |
| 99 | PRPF40A |  |  | 10644 | 1.163 | 0.2697 | No |
| 100 | HNRNPA1L2 |  |  | 10684 | 1.155 | 0.2758 | No |
| 101 | SRSF4 |  |  | 10841 | 1.129 | 0.2760 | No |
| 102 | SRSF2 |  |  | 11126 | 1.078 | 0.2697 | No |
| 103 | NCBP2 |  |  | 11695 | -1.018 | 0.2490 | No |
| 104 | PLRG1 |  |  | 11781 | -1.032 | 0.2529 | No |
| 105 | DDX46 |  |  | 12029 | -1.075 | 0.2485 | No |
| 106 | CCDC12 |  |  | 12160 | -1.098 | 0.2500 | No |
| 107 | LSM7 |  |  | 12357 | -1.139 | 0.2482 | No |
| 108 | LSM6 |  |  | 12458 | -1.163 | 0.2512 | No |
| 109 | PRPF8 |  |  | 12661 | -1.204 | 0.2491 | No |
| 110 | AQR |  |  | 13025 | -1.296 | 0.2388 | No |
| 111 | SRSF5 |  |  | 13534 | -1.470 | 0.2212 | No |
| 112 | DDX39B |  |  | 13594 | -1.495 | 0.2263 | No |
| 113 | RBM17 |  |  | 13753 | -1.572 | 0.2264 | No |
| 114 | SMNDC1 |  |  | 13910 | -1.643 | 0.2266 | No |
| 115 | EIF4A3 |  |  | 13961 | -1.668 | 0.2322 | No |
| 116 | SRSF8 |  |  | 14230 | -1.815 | 0.2267 | No |
| 117 | LSM8 |  |  | 15008 | -2.422 | 0.1954 | No |
| 118 | SNRPF |  |  | 15688 | -3.443 | 0.1691 | No |
| 119 | PRPF40B |  |  | 15898 | -3.834 | 0.1666 | No |
| 120 | HSPA6 |  |  | 17604 | -18.840 | 0.0882 | No |
| 121 | HSPA1A |  |  | 18005 | -37.190 | 0.0761 | No |
| 122 | HSPA1B |  |  | 18058 | -41.810 | 0.0815 | No |
| 123 | HSPA1L |  |  | 18060 | -41.890 | 0.0896 | No |
Table: GSEA details [plain text format]

  

Fig 2: KEGG\_SPLICEOSOME: Random ES distribution      
 Gene set null distribution of ES for **KEGG\_SPLICEOSOME**

  
