# Supplementary material for: DNA methylation-based classifier and gene expression signatures detect BRCAness in osteosarcoma
Source: PLoS Comput Biol. 2021 Nov 11;17(11):e1009562. doi: 10.1371/journal.pcbi.1009562 (PMC8584788; doi:10.1371/journal.pcbi.1009562)
Supplement: S2 File — (ZIP) [file pcbi.1009562.s002.zip › S2_File/my_analysis_Kegg.GseaPreranked.1581692187239/KEGG_TYPE_I_DIABETES_MELLITUS.html]

Details for gene set KEGG\_TYPE\_I\_DIABETES\_MELLITUS[GSEA]

|  || Dataset | DEG3\_two3dTopBottom |
| Phenotype | NoPhenotypeAvailable |
| Upregulated in class | na\_neg |
| GeneSet | KEGG\_TYPE\_I\_DIABETES\_MELLITUS |
| Enrichment Score (ES) | -0.60178274 |
| Normalized Enrichment Score (NES) | -0.60178274 |
| Nominal p-value | 0.0 |
| FDR q-value | 0.0 |
| FWER p-Value | 0.0 |
Table: GSEA Results Summary

  

Fig 1: Enrichment plot: KEGG\_TYPE\_I\_DIABETES\_MELLITUS      
 Profile of the Running ES Score & Positions of GeneSet Members on the Rank Ordered List

  

| PROBE | GENE SYMBOL | GENE\_TITLE | RANK IN GENE LIST | RANK METRIC SCORE | RUNNING ES | CORE ENRICHMENT || 1 | GAD1 |  |  | 283 | 618.800 | 0.0113 | No |
| 2 | HSPD1 |  |  | 6756 | 2.564 | -0.2901 | No |
| 3 | CPE |  |  | 7224 | 2.279 | -0.2880 | No |
| 4 | HLA-C |  |  | 10192 | 1.248 | -0.4123 | No |
| 5 | HLA-A |  |  | 13593 | -1.494 | -0.5585 | No |
| 6 | CD86 |  |  | 14450 | -1.967 | -0.5761 | Yes |
| 7 | IL1B |  |  | 14481 | -1.989 | -0.5520 | Yes |
| 8 | IL2 |  |  | 14536 | -2.032 | -0.5291 | Yes |
| 9 | HLA-B |  |  | 14983 | -2.404 | -0.5260 | Yes |
| 10 | IL12A |  |  | 15711 | -3.497 | -0.5371 | Yes |
| 11 | PTPRN2 |  |  | 15970 | -3.994 | -0.5245 | Yes |
| 12 | HLA-F |  |  | 16065 | -4.220 | -0.5036 | Yes |
| 13 | GAD2 |  |  | 16206 | -4.688 | -0.4850 | Yes |
| 14 | CD28 |  |  | 16514 | -5.798 | -0.4749 | Yes |
| 15 | HLA-G |  |  | 16786 | -7.343 | -0.4630 | Yes |
| 16 | TNF |  |  | 16858 | -7.878 | -0.4409 | Yes |
| 17 | ICA1 |  |  | 16901 | -8.226 | -0.4174 | Yes |
| 18 | IL12B |  |  | 17602 | -18.740 | -0.4271 | Yes |
| 19 | LTA |  |  | 17687 | -21.740 | -0.4057 | Yes |
| 20 | GZMB |  |  | 17776 | -25.560 | -0.3845 | Yes |
| 21 | HLA-DQA2 |  |  | 17900 | -31.160 | -0.3651 | Yes |
| 22 | FAS |  |  | 18057 | -41.750 | -0.3474 | Yes |
| 23 | HLA-DPB1 |  |  | 18263 | -63.760 | -0.3321 | Yes |
| 24 | HLA-DPA1 |  |  | 18376 | -82.260 | -0.3121 | Yes |
| 25 | HLA-DMA |  |  | 18388 | -85.320 | -0.2870 | Yes |
| 26 | HLA-DRB1 |  |  | 18392 | -86.860 | -0.2615 | Yes |
| 27 | HLA-DOA |  |  | 18426 | -97.930 | -0.2375 | Yes |
| 28 | HLA-DMB |  |  | 18587 | -159.700 | -0.2200 | Yes |
| 29 | HLA-E |  |  | 18671 | -203.600 | -0.1985 | Yes |
| 30 | HLA-DRA |  |  | 18720 | -232.200 | -0.1753 | Yes |
| 31 | HLA-DRB5 |  |  | 18733 | -243.500 | -0.1503 | Yes |
| 32 | HLA-DOB |  |  | 18766 | -268.900 | -0.1263 | Yes |
| 33 | CD80 |  |  | 18879 | -411.400 | -0.1063 | Yes |
| 34 | HLA-DQA1 |  |  | 18983 | -673.000 | -0.0859 | Yes |
| 35 | HLA-DQB1 |  |  | 19125 | -1584.000 | -0.0673 | Yes |
| 36 | FASLG |  |  | 19371 | -10130.000 | -0.0541 | Yes |
| 37 | IL1A |  |  | 19468 | -33230.000 | -0.0333 | Yes |
| 38 | PRF1 |  |  | 19503 | -62340.000 | -0.0094 | Yes |
| 39 | IFNG |  |  | 19720 | -4827000.000 | 0.0054 | Yes |
Table: GSEA details [plain text format]

  

Fig 2: KEGG\_TYPE\_I\_DIABETES\_MELLITUS: Random ES distribution      
 Gene set null distribution of ES for **KEGG\_TYPE\_I\_DIABETES\_MELLITUS**

  
